# Supplementary material for: Proton Detected Solid-State NMR of Membrane Proteins at 28 Tesla (1.2 GHz) and 100 kHz Magic-Angle Spinning
Source: Biomolecules. 2021 May 18;11(5):752. doi: 10.3390/biom11050752 (PMC8157399; doi:10.3390/biom11050752)
Supplement: Supplementary file 1 [file biomolecules-11-00752-s001.zip › biomolecules-1180594-SI.pdf]

Supplementary Information

Proton detected solid-state NMR of membrane proteins at 28 Tesla and 100 kHz magic-angle spinning

Evgeny Nimerovsky[1], Kumar Tekwani Movellan[1], Xizhou Zhang[1], Marcel C. Forster[1], Eszter Najbauer[1], Kai Xue[1], Rıza Dervişoğlu[1], Karin Giller[1], Christian Griesinger[1], Stefan Becker[1], Loren B. Andreas[1]*

[1]NMR-based Structural Biology, Max-Planck-Institute for Biophysical Chemistry, Göttingen, Germany

*Correspondence: land@nmr.mpibpc.mpg.de.com; Tel.: +49 551 201-2214

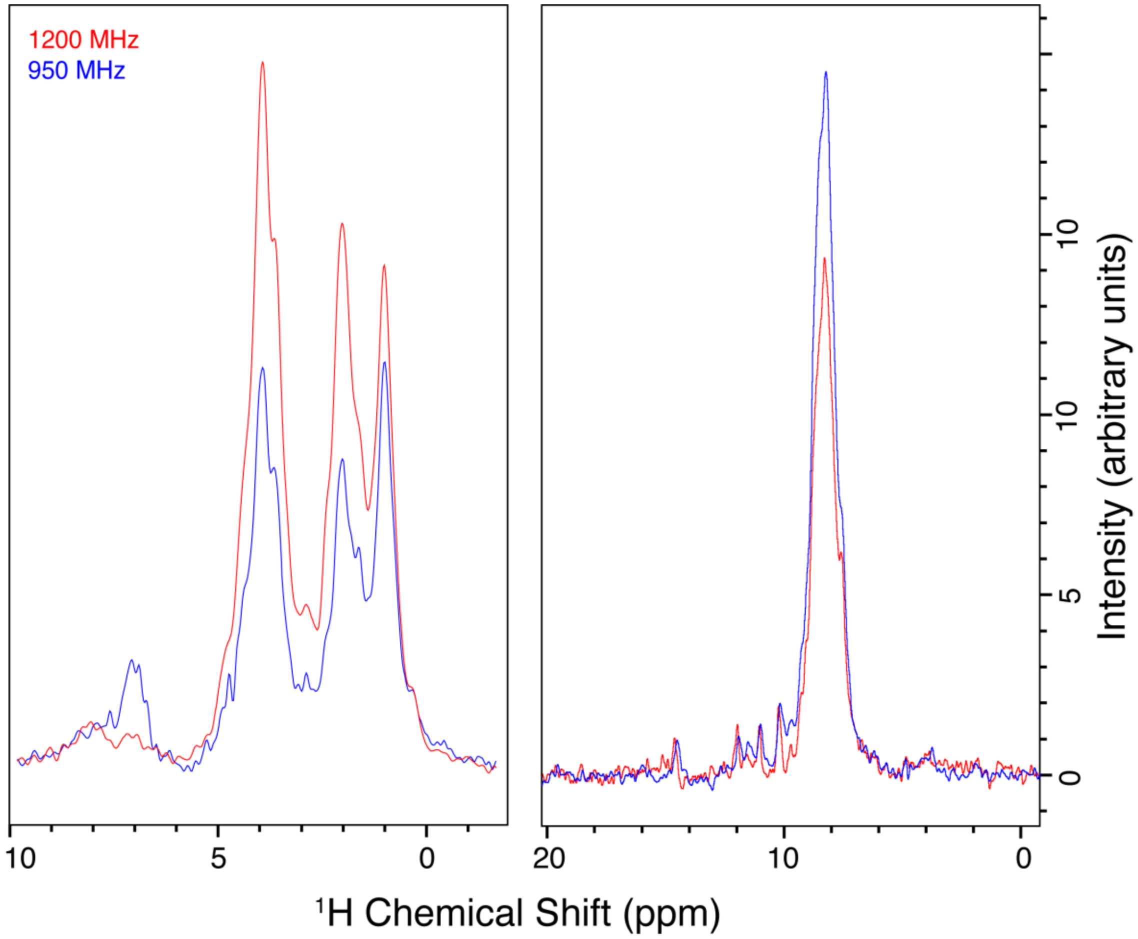

Figure S1. Example 1D spectra from which the sensitivity was estimated. Each spectrum is the average of 4 transients. The (HC)H spectrum is shown on the left, and the (HN)H spectrum is shown on the right. The spectra are displayed by scaling to the same noise level. The time domain signal was multiplied by a 100 Hz decaying exponential function before Fourier transformation. The signal at 7 ppm (left) indicates a better CP condition for aromatics in the 950 MHz spectrum (the optimization was performed for the aliphatic region).

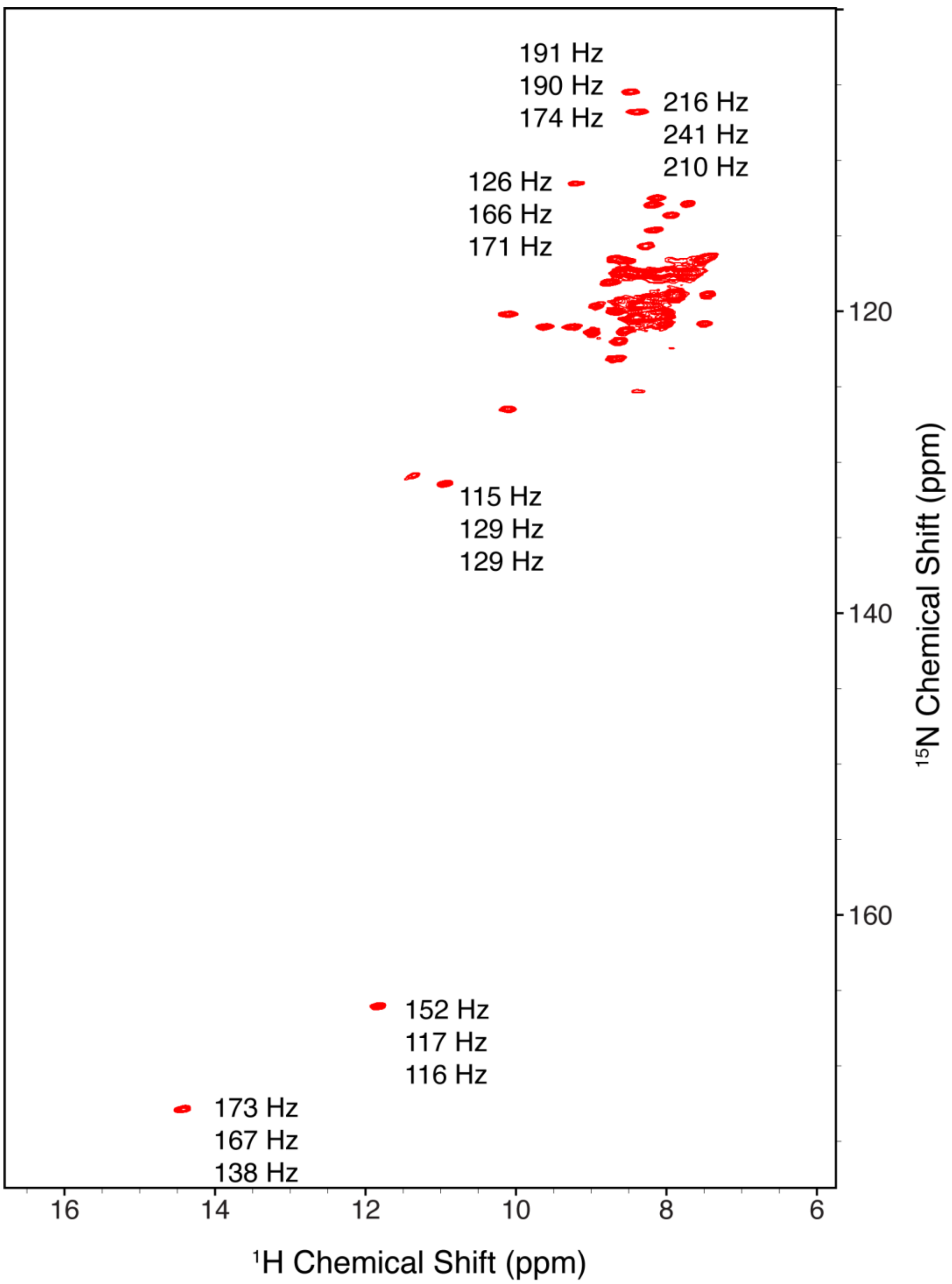

Figure S2. The 1200 MHz (H)NH spectrum of M2 (as in Figure 1) reacquired once the field drift had settled to less than 10 Hz per hour. The spectrum was acquired in 2 hours leading to a lower signal to noise ratio (S/N about 15) and larger variance in linewidth determination as compared with Figure 1 (S/N of about 30). The proton linewidth of three replicates is shown, the one from Figure 1, an additional one where the magic angle was adjusted based on proton T2′, and a third where the angle was adjusted by maximizing the $^{1}J_{NH}$ modulated signal at 10.87 ms. The variance in the linewidth is about 16 Hz, or about 10 percent.

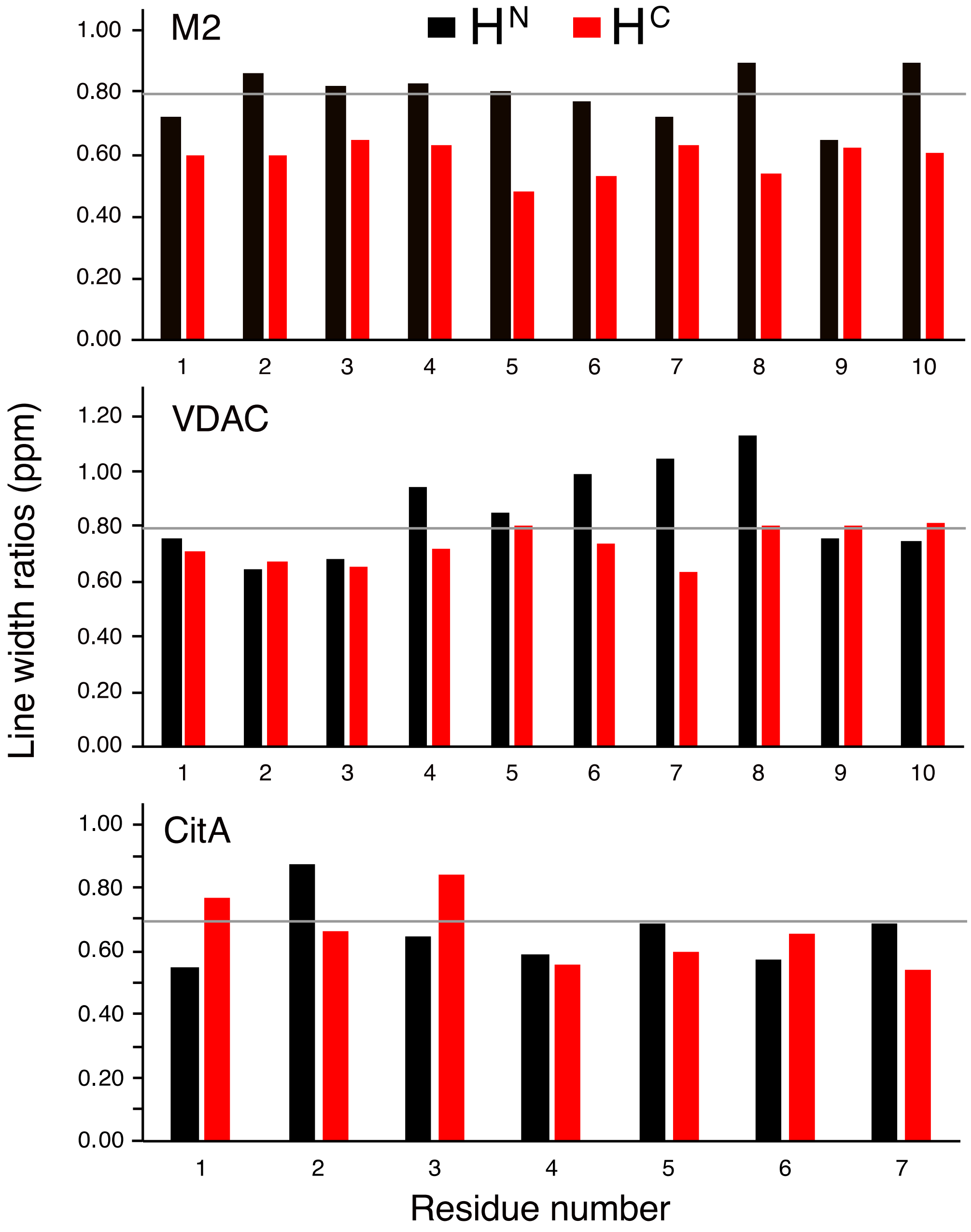

Figure S3. Detailed comparison of linewidth ratios on a peak-by-peak basis. The data is the same as that used for Figure 5. For Opa60, there were few isolated peaks to compare. The grey line indicates the field ratio, and the hypothetical improvement in ppm for the case where the linewidth remains the same as measured in Hz.

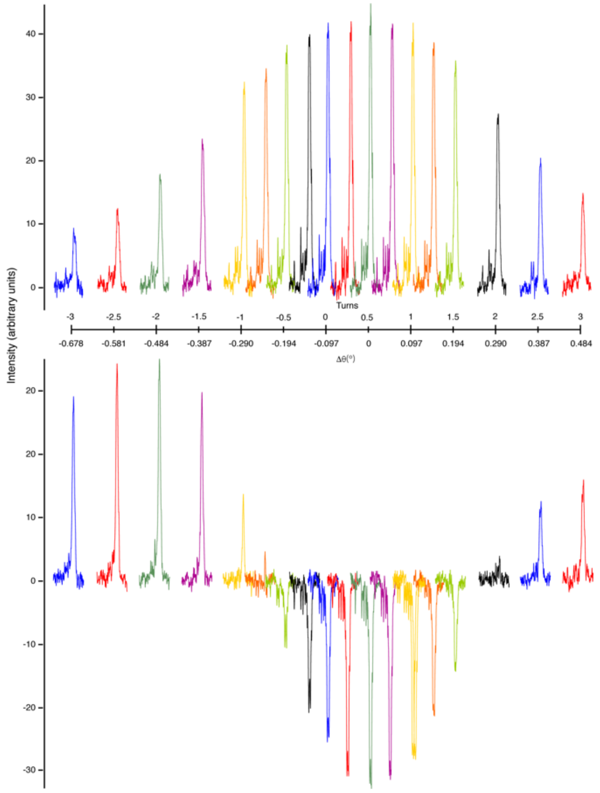

Figure S4. Example magic-angle adjustment using the proton T_2' (top) by maximizing signal in a proton Hahn echo after 3 ms, or by maximizing the $^1J_{HN}$ modulated nitrogen signal (bottom, negative max) recorded in a double Hahn echo at 10.87 ms, according to the procedure of Penzel et al.[1] The horizontal axis shows the number of turns relative to the previous angle setting (upper numbers) as well as the deviation from the magic angle in degrees, based on the dimensions supplied by the manufacturer (0.05 mm per turn, 14.8 mm ‘lever arm’). The sense of the deviation from the magic angle was not determined. 128 scans were recorded at each angle for each method. The proton T_2'-based method resulted in about 25 percent more signal, but was less sensitive to small deviations, suggesting a longer delay could have been used.

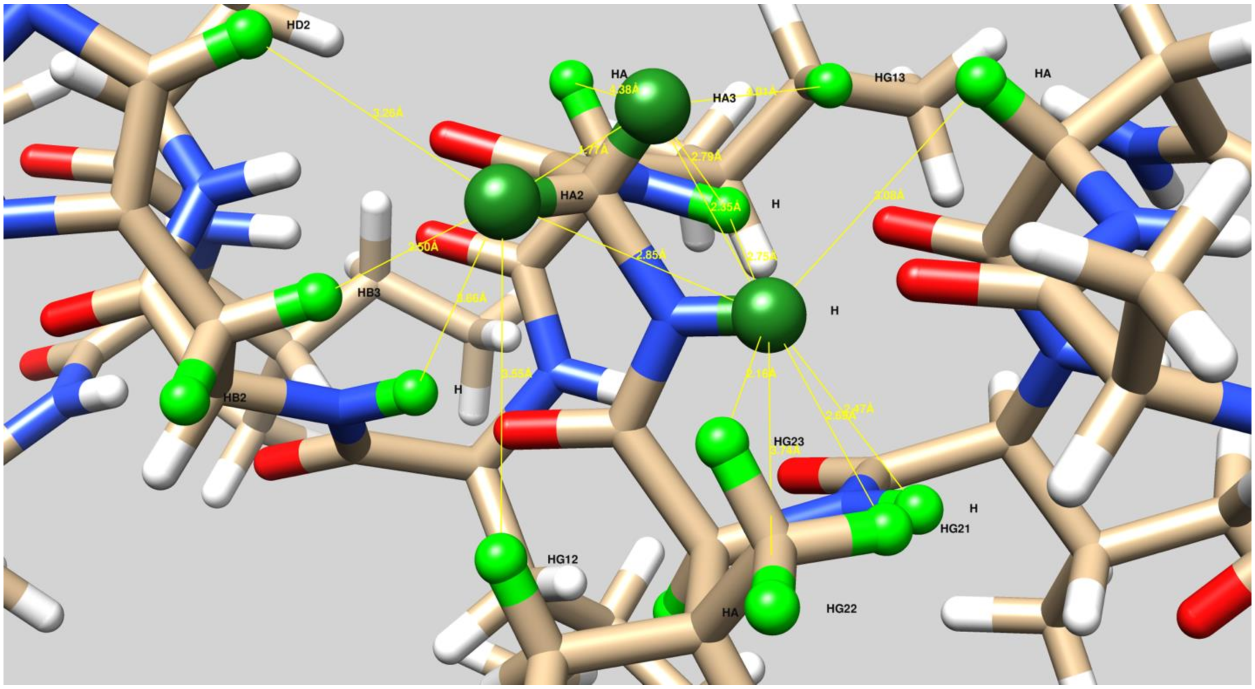

Figure S5. The schematic representation of 16 spins (Table S3), which were taken into linewidth simulations. The seven nearest protons to GLY34 H^{N} are: ILE33 H^{G23} (2.16 Å); ILE34 H^{A3} (2.35 Å); ILE33 H^{N} (2.47 Å); ILE33 H^{G21} (2.69 Å); ILE35 H^{N} (2.75 Å); ILE34 H^{A2} (2.85 Å); ASN31 H^{A} (3.08 Å). The seven nearest protons to GLY34 H^{A2} are: ILE34 H^{A3} (1.77 Å); HIS37 H^{B3} (2.49 Å); GLY34 H^{N} (2.85 Å); HIS37 H^{D2} (3.26 Å); ILE33 H^{G23} (3.44 Å); ILE33 H^{G12} (3.55 Å); ILE35 H^{N} (3.56 Å). The seven nearest protons to GLY34 H^{A3} are: ILE34 H^{A2} (1.77 Å); GLY34 H^{N} (2.35 Å); ILE35 H^{N} (2.79 Å); ASN31 H^{A} (3.22 Å); ILE33 H^{G23} (3.72 Å); ILE35 H^{13} (4.01 Å); HIS H^{B3} (4.03 Å). For Figures 7B-C, two additional protons were placed near the glycine alpha protons. For GLY34 H^{A2} the distances are: 2.6 Å and 2.86 Å; for GLY34 H^{A3} the distances are: 2.66 Å and 2.91 Å. For H^{N} the distances are 3.29 Å and 4.13 Å.

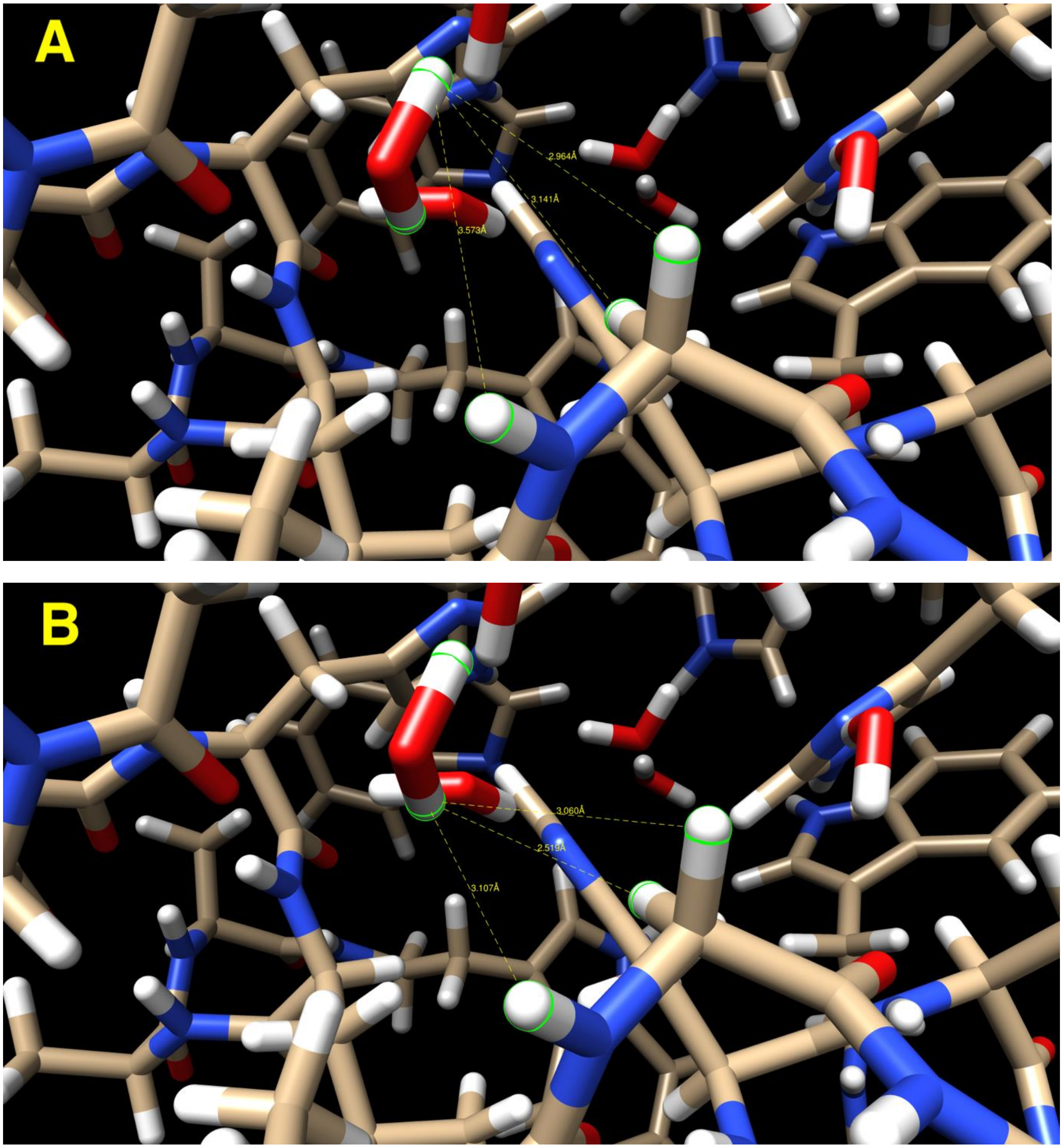

Figure S6. The schematic representation of the DFT calculated H_2O proton distances to the G34 protons, (A) depicting $H_2O:H^1$ and (B) H2O:H^2 distances. Here the neighboring environment of the H_2O in vicinity of G34 can be observed.

Tables

	M2	CitA	Opa60	VDAC
^1H (H-N)	119 to 149 kHz	118 to 147 kHz	113 to 141 kHz	116 to 145 kHz
^{15}N (H-N)	30 kHz	30 kHz	30 kHz	30 kHz

H-N contact	1000 µs	1000 µs	1000 µs	1000 µs
^{1}H (N-H)	137 to 110 kHz	110 to 137 kHz	136 to 109 kHz	110 to 138 kHz
^{15}N (N-H)	30 kHz	30 kHz	30 kHz	30 kHz
N-H contact	500 µs	500 µs	600 µs	600 µs
t1 max	31.4 ms	15.6 ms	44.3 ms	
Interscan delay	1.1 s	1.1 s	0.8 s	1.2 s
^{1}H (H-C)	111 to 139 kHz	115 to 135 kHz	113 to 141 kHz	113 to 133 kHz
^{13}C (H-C)	29 kHz	21 kHz	29 kHz	30 kHz
H-C contact	1200 µs	1200 µs	1200 µs	500 µs
^{1}H (C-H)	127 to 102 kHz	124 to 105 kHz	116 to 136 kHz	133 to 113 kHz
^{13}C (C-H)	29 kHz	21 kHz	31 kHz	30 kHz
C-H contact	350 µs	350 µs	350 µs	250 µs
t1 max	10.7 ms	8.4 ms	10 ms	
Interscan delay	1.1 s	1.1 s	0.8 s	1.2 s

Table S1. CP parameters for acquisition of the (H)CH and (H)NH spectra at 950 MHz spectrometer.

	M2	CitA	Opa60	VDAC
^{1}H (H-N)	140 to 174 kHz	148 to 185 kHz	142 to 178 kHz	146 to 183 kHz
^{15}N (H-N)	65 kHz	62 kHz	66 kHz	60 kHz
H-N contact	1400 µs	1400 µs	1400 µs	1000 µs
^{1}H (N-H)	174 to 140 kHz	180 to 144 kHz	178 to 142 kHz	179 to 143 kHz
^{15}N (N-H)	65 kHz	62 kHz	66 kHz	60 kHz
N-H contact	350 µs	350 µs	350 µs	600 µs
t1 max	63 ms	12.3 ms	24.9 ms	
Interscan delay	1.3 s	1.1 s	0.9 s	1.2 s
^{1}H (H-C)	142 to 178 kHz	154 to 192 kHz	155 to 194 kHz	154 to 193 kHz
^{13}C (H-C)	46 kHz	75 kHz	72 kHz	73 kHz
H-C contact	1200 µs	1200 µs	1200 µs	500 µs
^{1}H (C-H)	178 to 142 kHz	192 to 154 kHz	155 to 194 kHz	187 to 150 kHz
^{13}C (C-H)	46 kHz	75 kHz	74 kHz	73 kHz
C-H contact	250 µs	250 µs	250 µs	250 µs
t1 max	17 ms	8.5 ms	8.5 ms	
Interscan delay	1.3 s	1.1 s	1.0 s	1.2 s

Table S2. CP parameters for acquisition of the (H)CH and (H)NH spectra at 1200 MHz spectrometer.

		**Figure**				
		**7A,D,E,F**	**7A**	**7A**	**7B / 7C**	**7B /7C**
**Atom**	**$\delta_{iso,H}$ (ppm)**	**H^{N}**	**H^{A2}**	**H^{A3}**	**H^{N}**	**H^{A2}+ H^{A3}**
GLY34: H^{N}	8.193	1	3	3	1	3
GLY34: H^{A2}	4.307	2	1	2	2	1
GLY34: H^{A3}	3.551	3	2	1	3	2
ILE35: H^{N}	8.4	4	10	4	9	6

ILE33: H^{N}	8.5	5			10	
ILE33: H^{G21}	0.9	6	6	6	6	
ILE33: H^{G22}	0.9	7	7	7	7	
ILE33: H^{G23}	0.9	8	8	8	8	7
SER31:H^{A}	4.6	9		5	11	9
ILE33: H^{A}	4.3	10			12	
HIS37:H^{B2}	2.96		4			11
HIS37:H^{B3}	2.68		5	9		12
HIS37:H^{N}	8.3		9	10		
HIS37:H^{D2}	7.5		11			8
ILE33:H^{G12}	1.1	11	12			10
ILE33:H^{G13}	1.2	12				
ILE35: H^{A}	4.4			11		
ILE35:H^{G13}	1.2			12		
H_2X-	4.6(7B) / 3.21, 1.95 (7C)				4-5	4-5

Table S3 shows the set of spins that were used in the linewidths simulations for amide proton (H^{N}) and aliphatic protons (H^{A2}, H^{A3}). The number indicates the order in which spins were added. For Figure 7A, the spectrum was simulated as the sum of three separate simulated spectra, taking the closest spins for each of the amide and two alpha protons, as shown in columns 3-5 of the table. For panel Figure 7B and C in the main text, two additional protons were added, replacing other protons, as discussed in the main text. Both aliphatic protons were simulated together with the set of spins indicated in the last column. For 7B the H_2X- protons have the same $\delta_{iso,H}$ of 4.6 ppm, whereas for 7C 3.21 ppm and 1.95 ppm where used.

**Residue: H type**	**$\delta_{iso,H}$ /ppm (Exp.)**	**$\delta_{iso,H}$ / ppm (DFT)**
34: H^{N}	8.193	
34: H^{A2}	4.307	4.48
34: H^{A3}	3.551	4.08
35: H^{N}	8.4	
33: H^{N}	8.5	
33: H^{G21}	0.9	
33: H^{G22}	0.9	
33: H^{G23}	0.9	
31:H^{A}	4.6	
33: H^{A}	4.3	
37:H^{B2}	2.96	3.64
37:H^{B3}	2.68	3.48
37:H^{N}	8.3	8.40
37:H^{D2}	7.5	6.64
33:H^{G12}	1.1	
33:H^{G13}	1.2	
35: H^{A}	4.4	
35:H^{G13}	1.2	

Table S4: Comparison of Experimental DFT-derived chemical shifts. The correlation coefficient (R^2) for the six selected proton shifts of the DFT calculation is 0.97. These six were selected based on their central location in the calculation (e.g. G34 H^N was not included since it lacks the helical hydrogen bond in the simulation).

SCF GIAO Magnetic shielding tensor (ppm):

1 C Isotropic = 143.4529 Anisotropy = 41.2214

XX= 160.0080 YX= -17.4086 ZX= 10.2584

XY= -5.9307 YY= 142.9161 ZY= -5.0613

XZ= 14.7869 YZ= -10.7716 ZZ= 127.4346

Eigenvalues: 122.3139 137.1110 170.9338

2 C Isotropic = 24.2956 Anisotropy = 128.7991

XX= 32.5861 YX= 72.1333 ZX= -19.1418

XY= 71.3094 YY= 28.7887 ZY= -20.3684

XZ= -10.4394 YZ= -28.2718 ZZ= 11.5121

Eigenvalues: -42.1026 4.8278 110.1617

3 O Isotropic = -39.5001 Anisotropy = 529.6394

XX= 65.6967 YX= 201.4639 ZX= -106.7091

XY= 195.5440 YY= 53.8204 ZY= -154.6302

XZ= -90.8466 YZ= -141.6863 ZZ= -238.0173

Eigenvalues: -300.8868 -131.2062 313.5929

4 C Isotropic = 154.3323 Anisotropy = 16.2219

XX= 161.8744 YX= -1.9173 ZX= 6.3559

XY= -4.6322 YY= 154.5696 ZY= 1.3424

XZ= 8.0555 YZ= 4.1536 ZZ= 146.5530

Eigenvalues: 142.5055 155.3445 165.1469

5 C Isotropic = 157.5042 Anisotropy = 29.9821

XX= 166.0657 YX= -9.1341 ZX= 12.9088

XY= -8.5230 YY= 157.4829 ZY= -6.1217

XZ= 11.9125 YZ= -2.8759 ZZ= 148.9641

Eigenvalues: 142.4424 152.5780 177.4923

6 C Isotropic = 170.2829 Anisotropy = 24.2755

XX= 162.1965 YX= -1.1450 ZX= -3.3357

XY= -3.5596 YY= 162.6444 ZY= 1.7748

XZ= -3.1189 YZ= -4.0681 ZZ= 186.0080

Eigenvalues: 159.6735 164.7088 186.4666

7 C Isotropic = 171.7327 Anisotropy = 27.3622

XX= 172.2328 YX= 8.4601 ZX= 5.5827

XY= 14.8427 YY= 178.1671 ZY= 5.6626

XZ= 4.5685 YZ= 7.5283 ZZ= 164.7983

Eigenvalues: 162.0460 163.1779 189.9742

8 H Isotropic = 30.2392 Anisotropy = 10.2262

XX= 33.8627 YX= -3.2647 ZX= 3.4467

XY= -2.7512 YY= 30.7715 ZY= -1.9791

XZ= 3.2883 YZ= -1.8029 ZZ= 26.0835

Eigenvalues: 24.7218 28.9392 37.0567

9 H Isotropic = 29.4948 Anisotropy = 5.3275

XX= 30.9298 YX= 0.5716 ZX= -0.3621

XY= 0.0836 YY= 27.7583 ZY= -4.7118

XZ= -1.3019 YZ= -2.8563 ZZ= 29.7962

Eigenvalues: 24.8483 30.5896 33.0464

10 H Isotropic = 29.8910 Anisotropy = 4.3985

XX= 30.1321 YX= 0.3725 ZX= 2.4131

XY= 1.2278 YY= 31.4798 ZY= 1.4117

XZ= 3.8104 YZ= -0.9929 ZZ= 28.0611

Eigenvalues: 25.8012 31.0485 32.8234

11 H Isotropic = 30.3689 Anisotropy = 3.4154

XX= 30.8134 YX= 0.4473 ZX= 0.7178

XY= 0.3805 YY= 29.1237 ZY= 2.3954

XZ= -0.3133 YZ= 1.9785 ZZ= 31.1696

Eigenvalues: 27.7134 30.7475 32.6458

12 H Isotropic = 30.3875 Anisotropy = 9.1556

XX= 30.1110 YX= 0.7227 ZX= 1.8093

XY= 1.1173 YY= 30.1949 ZY= -6.2812

XZ= 1.6270 YZ= -5.5065 ZZ= 30.8567

Eigenvalues: 24.0553 30.6160 36.4913

13 H Isotropic = 30.8765 Anisotropy = 7.4470

XX= 32.1727 YX= -0.8665 ZX= 1.5707

XY= -0.8891 YY= 31.1697 ZY= -5.0773

XZ= 1.0073 YZ= -4.7402 ZZ= 29.2871

Eigenvalues: 25.2022 31.5861 35.8412

14 H Isotropic = 30.3468 Anisotropy = 7.7608

XX= 31.2835 YX= 4.3597 ZX= 1.0797

XY= 3.1448 YY= 30.8486 ZY= -4.6006

XZ= 1.8210 YZ= -3.7133 ZZ= 28.9084

Eigenvalues: 23.8888 31.6310 35.5207

15 H Isotropic = 30.6712 Anisotropy = 5.1517

XX= 29.8401 YX= -1.4797 ZX= 1.6433

XY= -1.6914 YY= 33.1901 ZY= 2.1256

XZ= 1.9686 YZ= 1.6012 ZZ= 28.9832

Eigenvalues: 26.6423 31.2656 34.1056

16 H Isotropic = 30.4062 Anisotropy = 8.4645

XX= 31.8059 YX= 4.8671 ZX= 2.6694

XY= 4.7022 YY= 30.3525 ZY= -3.9955

XZ= 1.7091 YZ= -3.6006 ZZ= 29.0603

Eigenvalues: 23.0998 32.0697 36.0492

17 H Isotropic = 30.7888 Anisotropy = 9.5862

XX= 28.5610 YX= 4.0756 ZX= 3.2902

XY= 2.9408 YY= 31.9527 ZY= 2.8107

XZ= 2.8544 YZ= 2.7071 ZZ= 31.8528

Eigenvalues: 26.0279 29.1590 37.1796

18 H Isotropic = 29.1793 Anisotropy = 16.8701

XX= 23.7167 YX= -0.8332 ZX= -0.1479

XY= -0.0509 YY= 24.6878 ZY= 4.1328

XZ= -0.2449 YZ= 4.8664 ZZ= 39.1333

Eigenvalues: 23.1539 23.9579 40.4260

19 N Isotropic = 147.2116 Anisotropy = 104.1249

XX= 162.7087 YX= -1.2488 ZX= -38.9448

XY= -23.9612 YY= 203.3356 ZY= 4.1287

XZ= -81.4015 YZ= 23.0333 ZZ= 75.5905

Eigenvalues: 44.6066 180.4000 216.6282

20 C   Isotropic =   144.1844   Anisotropy =   26.0113

XX=   157.5257   YX=   8.0663   ZX=   8.3655

XY=   8.1127   YY=   142.1037   ZY=   -11.1415

XZ=   8.6234   YZ=   -11.8516   ZZ=   132.9237

Eigenvalues: 121.4144   149.6135   161.5253

21 C   Isotropic =   27.6127   Anisotropy =   120.9751

XX=   33.3427   YX=   -64.0104   ZX=   -18.5590

XY=   -67.5625   YY=   11.9608   ZY=   27.9695

XZ=   -18.5577   YZ=   40.4021   ZZ=   37.5347

Eigenvalues: -46.6350   21.2104   108.2628

22 O   Isotropic =   -56.4707   Anisotropy =   573.0476

XX=   5.8984   YX= -150.9373   ZX= -197.9509

XY= -166.2858   YY=   -3.1238   ZY=   174.1447

XZ= -231.9749   YZ=   206.8137   ZZ= -172.1868

Eigenvalues: -338.3207 -156.6524   325.5610

23 H   Isotropic =   26.9373   Anisotropy =   10.0352

XX=   27.5106   YX=   -4.9994   ZX=   -0.0855

XY=   -4.2032   YY=   30.0230   ZY=   1.1041

XZ=   -0.1745   YZ=   1.1353   ZZ=   23.2784

Eigenvalues: 22.8903   24.2942   33.6275

24 H   Isotropic =   25.9238   Anisotropy =   3.9715

XX=   26.8979   YX=   -3.2126   ZX=   1.9318

XY=   -1.8123   YY=   23.6385   ZY=   1.5240

XZ=   0.6976   YZ=   1.2357   ZZ=   27.2350

Eigenvalues: 21.6618   27.5381   28.5715

25 H   Isotropic =   27.1430   Anisotropy =   7.0778

XX=   27.3723   YX=   1.1192   ZX=   -0.6596

XY=   -0.4198   YY=   28.1261   ZY=   -5.5211

XZ=   0.1463   YZ=   -3.8067   ZZ=   25.9304

Eigenvalues: 22.2369   27.3305   31.8615

26 N   Isotropic =   141.0018   Anisotropy =   115.0456

XX=   122.6826   YX=   14.3779   ZX=   -65.9385

XY=   21.9386   YY=   190.4394   ZY=   -2.7156

XZ=   -79.6487   YZ=   -39.8369   ZZ=   109.8835

Eigenvalues: 43.1261   162.1805   217.6989

27 C   Isotropic =   128.3621   Anisotropy =   22.0454

XX=   126.1615   YX=   -3.7915   ZX=   -7.9892

XY=   -1.3306   YY=   126.4007   ZY=   7.9136

XZ=   -9.9900   YZ=   8.7100   ZZ=   132.5241

Eigenvalues: 118.2815   123.7458   143.0590

28 C   Isotropic =   24.9401   Anisotropy =   122.4753

XX=   -5.5883   YX=   49.8107   ZX=   19.1445

XY=   44.4144   YY=   66.3237   ZY=   -52.4811

XZ=   5.4042   YZ=   -44.2472   ZZ=   14.0848

Eigenvalues: -48.9076   17.1375   106.5903

29 O   Isotropic =   -54.3311   Anisotropy =   558.4568

XX= -209.6756   YX=   115.7596   ZX= -123.6139

XY=   142.9160   YY=   198.7283   ZY= -138.8028

XZ= -160.5469   YZ= -180.3732   ZZ= -152.0460

Eigenvalues: -325.8357 -155.1310   317.9734

30 C   Isotropic =   150.5338   Anisotropy =   18.5904

XX=   147.1484   YX=   -4.6752   ZX=   -2.0953

XY=   -5.3318   YY=   143.7875   ZY=   -1.9484

XZ=   -5.3428   YZ=   -10.2729   ZZ=   160.6655

Eigenvalues: 137.9482   150.7258   162.9274

31 C   Isotropic =   162.7096   Anisotropy =   21.9426

XX=   157.1424   YX=   -8.8483   ZX=   -9.4167

XY=   -8.6248   YY=   162.8785   ZY=   3.5431

XZ=   -6.5562   YZ=   5.6528   ZZ=   168.1079

Eigenvalues: 149.8420   160.9488   177.3380

32 C   Isotropic =   175.4063   Anisotropy =   20.4501

XX=   181.5819   YX=   11.5435   ZX=   1.7588

XY=   5.2353   YY=   176.6084   ZY=   6.9674

XZ=   0.5256   YZ=   6.2591   ZZ=   168.0286

Eigenvalues: 163.7910 173.3883 189.0397

33 C Isotropic = 178.1304 Anisotropy = 26.2680

XX= 173.1316 YX= -7.1329 ZX= 0.4599

XY= -5.9011 YY= 169.2634 ZY= -12.0839

XZ= -1.6340 YZ= -7.1166 ZZ= 191.9961

Eigenvalues: 162.1003 176.6484 195.6424

34 H Isotropic = 30.5827 Anisotropy = 10.5574

XX= 26.7789 YX= 2.9000 ZX= -1.1533

XY= 3.3614 YY= 33.2582 ZY= -3.5755

XZ= -1.2565 YZ= -4.6680 ZZ= 31.7109

Eigenvalues: 25.4700 28.6570 37.6210

35 H Isotropic = 26.3312 Anisotropy = 8.4100

XX= 27.8347 YX= 3.4308 ZX= -1.5344

XY= 5.2210 YY= 25.0747 ZY= -3.5659

XZ= 0.6580 YZ= -2.8230 ZZ= 26.0841

Eigenvalues: 20.7921 26.2635 31.9378

36 H Isotropic = 27.5632 Anisotropy = 4.0976

XX= 28.8042 YX= -1.0480 ZX= 0.6562

XY= -2.2941 YY= 27.4380 ZY= -2.4076

XZ= -1.4029 YZ= -2.2732 ZZ= 26.4474

Eigenvalues: 24.1449 28.2498 30.2950

37 H Isotropic = 30.6940 Anisotropy = 3.5552

XX= 32.6396 YX= -0.7018 ZX= -0.8048

XY= -1.1417 YY= 29.0224 ZY= -3.4158

XZ= 0.1342 YZ= -2.4470 ZZ= 30.4200

Eigenvalues: 26.5646 32.4533 33.0642

38 H Isotropic = 30.3885 Anisotropy = 6.8599

XX= 26.8889 YX= 1.5577 ZX= -2.9371

XY= 2.3934 YY= 32.6198 ZY= -1.4687

XZ= -2.3482 YZ= -1.4968 ZZ= 31.6569

Eigenvalues: 25.4911 30.7127 34.9617

39 H Isotropic = 30.8759 Anisotropy = 7.3318

XX=   27.4564   YX=   0.8779   ZX=   -3.2902

XY=   0.4732   YY=   34.6179   ZY=   2.2091

XZ=   -3.4094   YZ=   2.4398   ZZ=   30.5535

Eigenvalues: 24.9590   31.9049   35.7638

40 H   Isotropic =   31.1523   Anisotropy =   8.8640

XX=   33.3088   YX=   0.0516   ZX=   2.6613

XY=   -0.9255   YY=   26.9615   ZY=   -2.9218

XZ=   3.7968   YZ=   -2.9659   ZZ=   33.1866

Eigenvalues: 25.6937   30.7015   37.0616

41 H   Isotropic =   29.6355   Anisotropy =   13.5648

XX=   36.5355   YX=   -4.6388   ZX=   0.5288

XY=   -5.9027   YY=   24.7564   ZY=   -1.9510

XZ=   0.5527   YZ=   -1.8680   ZZ=   27.6146

Eigenvalues: 22.2618   27.9659   38.6787

42 H   Isotropic =   30.6225   Anisotropy =   11.2780

XX=   27.2559   YX=   3.9816   ZX=   -2.9803

XY=   4.1670   YY=   33.5154   ZY=   -3.9294

XZ=   -1.9085   YZ=   -3.2168   ZZ=   31.0961

Eigenvalues: 25.1578   28.5685   38.1411

43 H   Isotropic =   30.6305   Anisotropy =   10.8875

XX=   27.6414   YX=   -1.9968   ZX=   -3.2205

XY=   -1.5542   YY=   27.6264   ZY=   0.2298

XZ=   -3.4282   YZ=   1.3451   ZZ=   36.6237

Eigenvalues: 25.5014   28.5012   37.8888

44 H   Isotropic =   30.7456   Anisotropy =   5.5620

XX=   33.7058   YX=   -0.5876   ZX=   1.8705

XY=   0.9330   YY=   29.0182   ZY=   -1.5474

XZ=   1.8982   YZ=   -1.3610   ZZ=   29.5128

Eigenvalues: 27.4715   30.3117   34.4536

45 N   Isotropic =   136.9352   Anisotropy =   106.1864

XX=   162.5325   YX=   -39.1676   ZX=   -74.2990

XY=   -38.6657   YY=   168.7293   ZY=   -16.5830

XZ= -37.4557 YZ= -43.6406 ZZ= 79.5439

Eigenvalues: 36.2335 166.8460 207.7262

46 C Isotropic = 137.0214 Anisotropy = 23.0977

XX= 120.2301 YX= 3.4519 ZX= -1.5590

XY= 6.1162 YY= 141.1330 ZY= -4.7997

XZ= -1.7141 YZ= -5.2728 ZZ= 149.7012

Eigenvalues: 119.1778 139.4667 152.4199

47 C Isotropic = 24.2063 Anisotropy = 117.6375

XX= 83.9442 YX= -39.1842 ZX= -14.8370

XY= -34.2910 YY= -18.6855 ZY= 36.3671

XZ= -18.3402 YZ= 24.1734 ZZ= 7.3601

Eigenvalues: -42.8329 12.8204 102.6313

48 O Isotropic = -35.7197 Anisotropy = 519.4038

XX= 207.9287 YX= -161.8189 ZX= -160.5619

XY= -154.4541 YY= -112.4952 ZY= 7.4216

XZ= -145.2170 YZ= -26.1512 ZZ= -202.5925

Eigenvalues: -280.2408 -137.4677 310.5495

49 C Isotropic = 174.3706 Anisotropy = 28.4209

XX= 187.0718 YX= -13.6905 ZX= 3.1534

XY= -8.9590 YY= 169.4292 ZY= -1.6258

XZ= 4.9311 YZ= -1.6997 ZZ= 166.6109

Eigenvalues: 163.8579 165.9361 193.3179

50 H Isotropic = 30.2622 Anisotropy = 7.8901

XX= 32.5013 YX= 3.4823 ZX= 2.3369

XY= 1.8780 YY= 32.5427 ZY= 0.6056

XZ= 1.3524 YZ= 0.6851 ZZ= 25.7425

Eigenvalues: 25.2716 29.9926 35.5223

51 H Isotropic = 26.4148 Anisotropy = 4.6726

XX= 28.8424 YX= -0.2109 ZX= -1.1123

XY= 0.4344 YY= 25.3941 ZY= 2.1220

XZ= -1.7802 YZ= 3.2844 ZZ= 25.0079

Eigenvalues: 22.2899 27.4246 29.5299

52 H Isotropic = 27.7192 Anisotropy = 5.6405

XX= 27.2387 YX= 1.9756 ZX= 1.3173

XY= 2.2007 YY= 29.1796 ZY= 3.0875

XZ= 1.3768 YZ= 0.2697 ZZ= 26.7393

Eigenvalues: 25.6169 26.0611 31.4795

53 H Isotropic = 30.6209 Anisotropy = 11.2228

XX= 28.1220 YX= -2.6655 ZX= 1.9571

XY= -2.5168 YY= 34.3502 ZY= -4.7078

XZ= 1.0808 YZ= -4.7251 ZZ= 29.3905

Eigenvalues: 26.5396 27.2204 38.1028

54 H Isotropic = 30.1128 Anisotropy = 8.5593

XX= 29.0249 YX= -3.7516 ZX= -2.1001

XY= -3.2521 YY= 31.4281 ZY= 2.9878

XZ= -1.3888 YZ= 2.7467 ZZ= 29.8853

Eigenvalues: 26.4920 28.0273 35.8190

55 N Isotropic = 136.5372 Anisotropy = 89.2312

XX= 176.1739 YX= 22.1031 ZX= -35.4375

XY= -0.7143 YY= 186.5832 ZY= -23.2553

XZ= -28.8225 YZ= 20.0557 ZZ= 46.8545

Eigenvalues: 39.3063 174.2807 196.0247

56 C Isotropic = 135.8375 Anisotropy = 15.7272

XX= 143.8216 YX= -4.2575 ZX= -0.4081

XY= -4.7988 YY= 124.0787 ZY= -10.2173

XZ= 1.2664 YZ= -7.5372 ZZ= 139.6123

Eigenvalues: 119.4069 141.7834 146.3224

57 C Isotropic = 24.2263 Anisotropy = 121.1716

XX= -41.9071 YX= 15.5437 ZX= -5.9897

XY= 12.8992 YY= 103.4523 ZY= -4.8327

XZ= 8.4833 YZ= -3.5943 ZZ= 11.1337

Eigenvalues: -43.3353 11.0069 105.0074

58 O Isotropic = -39.6833 Anisotropy = 524.5264

XX= -131.6486 YX= 54.2969 ZX= -2.4861

XY= 41.4660 YY= 303.8960 ZY= -43.2132

XZ= 15.7490 YZ= -5.1056 ZZ= -291.2973

Eigenvalues: -292.7426 -136.3083 310.0010

59 C Isotropic = 160.3163 Anisotropy = 22.2128

XX= 155.1459 YX= 9.8441 ZX= 1.3256

XY= 9.9288 YY= 169.5187 ZY= -3.1881

XZ= -0.5056 YZ= -4.5512 ZZ= 156.2844

Eigenvalues: 149.4119 156.4122 175.1249

60 C Isotropic = 58.4407 Anisotropy = 119.2360

XX= 115.9477 YX= -49.2735 ZX= 0.7925

XY= -51.5835 YY= 19.2515 ZY= 29.4104

XZ= 1.6833 YZ= 10.5257 ZZ= 40.1230

Eigenvalues: -9.4721 46.8629 137.9314

61 C Isotropic = 82.0268 Anisotropy = 107.0275

XX= 135.7499 YX= -39.7004 ZX= -1.6335

XY= -52.4213 YY= 33.0248 ZY= -9.0595

XZ= -5.7018 YZ= -8.9924 ZZ= 77.3058

Eigenvalues: 13.9003 78.8017 153.3785

62 N Isotropic = 1.4037 Anisotropy = 324.2602

XX= 151.8244 YX= -145.9032 ZX= -5.0398

XY= -134.8940 YY= -85.8498 ZY= 31.9884

XZ= 13.3948 YZ= 49.6043 ZZ= -61.7635

Eigenvalues: -165.5061 -47.8599 217.5772

63 C Isotropic = 68.3796 Anisotropy = 107.5461

XX= 113.2037 YX= -59.2500 ZX= -9.8105

XY= -49.8254 YY= 29.3482 ZY= -21.6080

XZ= -1.6355 YZ= -5.4342 ZZ= 62.5868

Eigenvalues: -0.9004 65.9621 140.0770

64 N Isotropic = 110.1504 Anisotropy = 126.6940

XX= 174.9161 YX= -37.2718 ZX= 14.3526

XY= -39.1835 YY= 119.3730 ZY= 22.7754

XZ= 22.8637 YZ= 25.1548 ZZ= 36.1619

Eigenvalues: 24.2331 111.6050 194.6130

65 H Isotropic = 23.9886 Anisotropy = 16.2707

XX= 18.5471 YX= -0.6433 ZX= -8.1639

XY= 1.5224 YY= 20.2710 ZY= 0.9675

XZ= -2.1412 YZ= 1.1490 ZZ= 33.1478

Eigenvalues: 16.7547 20.3755 34.8358

66 H Isotropic = 22.6706 Anisotropy = 8.7632

XX= 19.1285 YX= 2.6292 ZX= 5.9541

XY= 3.8018 YY= 22.6591 ZY= 0.5732

XZ= 2.7368 YZ= -1.2594 ZZ= 26.2243

Eigenvalues: 15.7085 23.7906 28.5128

67 H Isotropic = 27.7051 Anisotropy = 5.5335

XX= 26.2144 YX= 1.0407 ZX= -3.4498

XY= 0.8397 YY= 26.5634 ZY= -0.6759

XZ= -0.3348 YZ= -1.2464 ZZ= 30.3375

Eigenvalues: 25.2204 26.5008 31.3941

68 H Isotropic = 28.2536 Anisotropy = 6.3091

XX= 27.9687 YX= 1.1142 ZX= -4.8945

XY= -0.0229 YY= 28.0274 ZY= 1.6033

XZ= -3.2131 YZ= 0.1723 ZZ= 28.7646

Eigenvalues: 24.0416 28.2595 32.4596

69 H Isotropic = 25.7540 Anisotropy = 8.4900

XX= 23.5244 YX= 3.7478 ZX= -2.1126

XY= 3.9866 YY= 24.4595 ZY= -2.8799

XZ= -1.5088 YZ= -1.2452 ZZ= 29.2781

Eigenvalues: 20.0966 25.7515 31.4140

70 H Isotropic = 27.3498 Anisotropy = 12.1268

XX= 23.5690 YX= 5.5048 ZX= 1.4671

XY= 4.5297 YY= 33.2931 ZY= 0.7674

XZ= 1.3517 YZ= -1.0690 ZZ= 25.1873

Eigenvalues: 21.0033 25.6117 35.4343

71 H Isotropic = 28.6978 Anisotropy = 6.5705

XX= 29.6270 YX= 1.8223 ZX= 4.0561

XY= 2.0330 YY= 27.5294 ZY= -3.5827

XZ= 3.2378 YZ= -2.7577 ZZ= 28.9372

Eigenvalues: 22.7904 30.2250 33.0782

72 N Isotropic = 140.5431 Anisotropy = 88.5915

XX= 164.1033 YX= 1.0257 ZX= -39.7945

XY= -10.7540 YY= 179.6472 ZY= 13.3039

XZ= -82.6940 YZ= 5.1263 ZZ= 77.8787

Eigenvalues: 45.8297 176.1955 199.6041

73 C Isotropic = 134.8033 Anisotropy = 18.5064

XX= 132.8118 YX= 1.0918 ZX= 11.0783

XY= 4.7083 YY= 142.5644 ZY= 3.8001

XZ= 8.5069 YZ= 5.7328 ZZ= 129.0337

Eigenvalues: 120.7855 136.4834 147.1409

74 C Isotropic = 26.8690 Anisotropy = 114.7722

XX= 80.5918 YX= 3.7405 ZX= -35.0061

XY= -4.6980 YY= -42.4855 ZY= -15.9065

XZ= -39.1044 YZ= -4.9557 ZZ= 42.5007

Eigenvalues: -43.9692 21.1923 103.3838

75 O Isotropic = -49.2053 Anisotropy = 569.3763

XX= 150.0694 YX= 44.2196 ZX= -266.3504

XY= 33.1718 YY= -159.7652 ZY= 33.9516

XZ= -314.0170 YZ= 40.7835 ZZ= -137.9203

Eigenvalues: -333.5452 -144.4496 330.3789

76 C Isotropic = 154.0524 Anisotropy = 25.0523

XX= 167.0610 YX= -13.8757 ZX= 2.5442

XY= -5.9786 YY= 144.0368 ZY= 0.3373

XZ= -0.7735 YZ= 2.8534 ZZ= 151.0594

Eigenvalues: 140.0521 151.3512 170.7540

77 C Isotropic = 164.2696 Anisotropy = 5.7525

XX= 166.6912 YX= -2.5402 ZX= -2.2710

XY= -3.5247 YY= 160.4938 ZY= -1.3143

XZ= 1.6294 YZ= -3.3244 ZZ= 165.6239

Eigenvalues: 158.5278 166.1764 168.1046

78 C Isotropic = 169.0589 Anisotropy = 30.8154

XX= 158.3807 YX= 0.1325 ZX= -0.7127

XY= 4.3704 YY= 176.3237 ZY= -17.5231

XZ= 1.0830 YZ= -12.4819 ZZ= 172.4721

Eigenvalues: 157.0920 160.4821 189.6025

79 C Isotropic = 162.1657 Anisotropy = 30.4571

XX= 156.6445 YX= -0.8963 ZX= -12.5880

XY= -2.9343 YY= 153.4445 ZY= 0.0006

XZ= -12.0118 YZ= -6.6837 ZZ= 176.4081

Eigenvalues: 148.6066 155.4200 182.4704

80 H Isotropic = 30.6312 Anisotropy = 7.6524

XX= 26.8336 YX= -1.1437 ZX= -1.3894

XY= 0.0523 YY= 32.1193 ZY= 2.8391

XZ= -0.9158 YZ= 3.1713 ZZ= 32.9408

Eigenvalues: 26.6233 29.5375 35.7328

81 H Isotropic = 24.8410 Anisotropy = 5.0120

XX= 25.6787 YX= -1.4449 ZX= -1.0758

XY= 0.0553 YY= 20.6804 ZY= 0.9533

XZ= 1.5014 YZ= -0.7233 ZZ= 28.1638

Eigenvalues: 20.5829 25.7577 28.1823

82 H Isotropic = 28.8994 Anisotropy = 4.7830

XX= 29.1285 YX= 0.6838 ZX= -0.2747

XY= -2.2153 YY= 30.0763 ZY= -3.2474

XZ= -0.8372 YZ= -2.7476 ZZ= 27.4933

Eigenvalues: 25.3145 29.2956 32.0880

83 H Isotropic = 30.0666 Anisotropy = 6.9931

XX= 28.8690 YX= -3.5506 ZX= -1.1632

XY= -3.4147 YY= 29.9888 ZY= -3.1858

XZ= -0.4189 YZ= -3.6730 ZZ= 31.3419

Eigenvalues: 24.6134 30.8576 34.7286

84 H Isotropic = 30.0227 Anisotropy = 7.1482

XX= 29.8321 YX= 0.9617 ZX= -0.8879

XY= 1.9038 YY= 32.2180 ZY= 4.0035

XZ= -1.1707 YZ= 4.1110 ZZ= 28.0179

Eigenvalues: 24.9982 30.2816 34.7882

85 H Isotropic = 31.2053 Anisotropy = 6.9178

XX= 33.2737 YX= -1.3892 ZX= 2.6350

XY= -1.5888 YY= 29.5549 ZY= -1.1927

XZ= 2.6552 YZ= -2.7207 ZZ= 30.7874

Eigenvalues: 28.0757 29.7230 35.8172

86 H Isotropic = 30.5742 Anisotropy = 8.1333

XX= 35.9327 YX= 0.5960 ZX= 0.2896

XY= 0.8562 YY= 27.4340 ZY= -0.2521

XZ= -0.5251 YZ= 0.0855 ZZ= 28.3558

Eigenvalues: 27.3670 28.3592 35.9964

87 H Isotropic = 30.7381 Anisotropy = 4.3614

XX= 30.0657 YX= 0.1580 ZX= -0.8064

XY= -0.2551 YY= 31.2052 ZY= 2.2298

XZ= -0.0523 YZ= 2.8448 ZZ= 30.9434

Eigenvalues: 28.4841 30.0845 33.6457

88 H Isotropic = 32.0935 Anisotropy = 12.3196

XX= 28.9407 YX= 2.3710 ZX= -0.6388

XY= 4.9517 YY= 39.1270 ZY= 0.5644

XZ= -0.4542 YZ= -0.1315 ZZ= 28.2129

Eigenvalues: 27.3586 28.6155 40.3066

89 H Isotropic = 30.8515 Anisotropy = 8.9637

XX= 28.7940 YX= 1.8488 ZX= -2.6525

XY= 1.4231 YY= 29.3826 ZY= -2.6059

XZ= -1.7715 YZ= -3.7285 ZZ= 34.3778

Eigenvalues: 27.3829 28.3442 36.8273

90 H Isotropic = 30.3122 Anisotropy = 8.4737

XX= 27.9970 YX= -2.1138 ZX= -2.2198

XY= -0.0072 YY= 28.2063 ZY= -0.2917

XZ= -3.9724 YZ= -1.3965 ZZ= 34.7332

Eigenvalues: 26.0184 28.9568 35.9613

91 N Isotropic = 135.9926 Anisotropy = 95.1450

XX= 133.8560 YX= -25.9438 ZX= -65.5420

XY= -10.1195 YY= 188.6224 ZY= -12.4341

XZ= -54.4059 YZ= -57.7022 ZZ= 85.4993

Eigenvalues: 35.0224 173.5328 199.4225

92 C Isotropic = 136.4352 Anisotropy = 22.5747

XX= 138.1680 YX= 5.2675 ZX= -7.1461

XY= 7.6093 YY= 126.4482 ZY= 10.8798

XZ= -8.2787 YZ= 10.8692 ZZ= 144.6894

Eigenvalues: 117.3020 140.5187 151.4850

93 C Isotropic = 26.0034 Anisotropy = 116.0317

XX= -16.6807 YX= -26.7653 ZX= 44.9457

XY= -25.8349 YY= 96.8147 ZY= 2.1460

XZ= 30.0235 YZ= -2.8360 ZZ= -2.1237

Eigenvalues: -50.3935 25.0459 103.3579

94 O Isotropic = -56.2318 Anisotropy = 572.3406

XX= -196.6650 YX= -161.5997 ZX= -54.1324

XY= -142.0002 YY= 273.0708 ZY= -61.3425

XZ= -75.4287 YZ= -111.4312 ZZ= -245.1012

Eigenvalues: -333.1970 -160.8270 325.3286

95 C Isotropic = 174.2436 Anisotropy = 25.1523

XX= 163.5728 YX= -5.2873 ZX= 3.6837

XY= -1.5029 YY= 184.9611 ZY= -8.5710

XZ= 3.4701 YZ= -9.7327 ZZ= 174.1968

Eigenvalues: 162.4589 169.2600 191.0118

96 H Isotropic = 30.4156 Anisotropy = 12.3500

XX= 26.0106 YX= 2.8560 ZX= 1.1587

XY= 3.5566 YY= 37.4757 ZY= 2.0231

XZ= 1.0504 YZ= 1.3576 ZZ= 27.7604

Eigenvalues: 25.0082 27.5896 38.6489

97 H Isotropic = 24.6950 Anisotropy = 4.3978

XX= 22.0141 YX= 0.2591 ZX= 2.5073

XY= 0.0513 YY= 27.0665 ZY= -0.2564

XZ= 4.4080 YZ= 1.1117 ZZ= 25.0045

Eigenvalues: 19.7407 26.7175 27.6269

98 H Isotropic = 27.6610 Anisotropy = 6.5760

XX= 29.7904 YX= 2.3537 ZX= 3.1770

XY= 1.8469 YY= 28.6492 ZY= 2.3458

XZ= 0.3853 YZ= 0.2588 ZZ= 24.5433

Eigenvalues: 23.8940 27.0440 32.0450

99 H Isotropic = 29.7706 Anisotropy = 5.1702

XX= 32.0968 YX= 2.0447 ZX= 2.2009

XY= 0.3594 YY= 30.5335 ZY= -1.0531

XZ= 2.4438 YZ= -0.8284 ZZ= 26.6815

Eigenvalues: 25.4819 30.6124 33.2174

100 H Isotropic = 30.7243 Anisotropy = 7.8801

XX= 27.4513 YX= 2.6240 ZX= -2.0881

XY= 2.5509 YY= 31.0736 ZY= -1.9454

XZ= -2.7362 YZ= -1.8003 ZZ= 33.6479

Eigenvalues: 25.8586 30.3365 35.9777

101 N Isotropic = 141.6196 Anisotropy = 92.2951

XX= 193.3722 YX= -3.9563 ZX= -59.1147

XY= -7.3783 YY= 169.0554 ZY= -32.7897

XZ= -14.6690 YZ= -23.6588 ZZ= 62.4310

Eigenvalues: 46.0377 175.6714 203.1496

102 C Isotropic = 137.3469 Anisotropy = 25.4436

XX= 118.2084 YX= -9.8140 ZX= 1.5722

XY= -7.0498 YY= 144.7170 ZY= -6.1170

XZ= -0.6988 YZ= -6.2572 ZZ= 149.1153

Eigenvalues: 115.7005 142.0309 154.3093

103 C Isotropic = 25.6719 Anisotropy = 118.9043

XX=   77.6916   YX=   48.8523   ZX=   -25.4065

XY=   57.1993   YY=   -21.9228   ZY=   11.6384

XZ=   -20.5862   YZ=   -0.1754   ZZ=   21.2468

Eigenvalues: -47.9646   20.0388   104.9414

104 O   Isotropic =   -29.4542   Anisotropy =   547.1340

XX=   244.1518   YX=   148.6719   ZX= -136.2356

XY=   162.3377   YY=   -80.9265   ZY=   -78.7846

XZ=   -74.7114   YZ=   -91.0861   ZZ= -251.5877

Eigenvalues: -290.3287 -133.3356   335.3018

105 C   Isotropic =   171.6047   Anisotropy =   21.8962

XX=   184.0507   YX=   4.8586   ZX=   -2.4572

XY=   8.4672   YY=   165.1936   ZY=   0.2242

XZ=   0.6329   YZ=   -0.0815   ZZ=   165.5697

Eigenvalues: 163.0297   165.5821   186.2022

106 H   Isotropic =   30.7050   Anisotropy =   6.1670

XX=   29.4714   YX=   3.9404   ZX=   -0.5192

XY=   1.3136   YY=   32.6108   ZY=   3.9476

XZ=   -1.4997   YZ=   1.1434   ZZ=   30.0329

Eigenvalues: 26.5221   30.7766   34.8164

107 H   Isotropic =   23.5547   Anisotropy =   8.1611

XX=   22.6069   YX=   3.2612   ZX=   1.7602

XY=   2.9695   YY=   19.8500   ZY=   1.7928

XZ=   -0.4347   YZ=   2.2457   ZZ=   28.2072

Eigenvalues: 17.6476   24.0210   28.9954

108 H   Isotropic =   27.7097   Anisotropy =   7.9658

XX=   24.6753   YX=   1.7648   ZX=   -1.9486

XY=   1.1210   YY=   30.3638   ZY=   5.1385

XZ=   -0.1560   YZ=   2.0217   ZZ=   28.0900

Eigenvalues: 23.3070   26.8018   33.0203

109 H   Isotropic =   30.6544   Anisotropy =   6.1723

XX=   31.3705   YX=   -2.5568   ZX=   1.4987

XY=   -3.0124   YY=   30.9286   ZY=   -0.7432

XZ= 1.2505 YZ= -2.3311 ZZ= 29.6640

Eigenvalues: 28.2944 28.8995 34.7692

110 H Isotropic = 30.0311 Anisotropy = 8.9708

XX= 31.3232 YX= 2.0747 ZX= -4.6582

XY= 1.0380 YY= 27.7391 ZY= 0.3306

XZ= -4.3509 YZ= -1.8399 ZZ= 31.0312

Eigenvalues: 26.3935 27.6882 36.0117

111 N Isotropic = 149.7719 Anisotropy = 99.4763

XX= 173.2786 YX= 7.9465 ZX= -28.0670

XY= -8.5397 YY= 208.7037 ZY= 7.9772

XZ= -41.6381 YZ= 51.1680 ZZ= 67.3336

Eigenvalues: 51.7934 181.4329 216.0895

112 C Isotropic = 146.1554 Anisotropy = 35.1500

XX= 138.1227 YX= -5.0869 ZX= 5.0336

XY= -12.7919 YY= 163.4928 ZY= -17.8558

XZ= -4.2149 YZ= -3.4901 ZZ= 136.8507

Eigenvalues: 131.6114 137.2661 169.5888

113 C Isotropic = 162.0716 Anisotropy = 20.9167

XX= 148.2956 YX= -7.7826 ZX= 8.0101

XY= -7.9597 YY= 173.7533 ZY= -4.1369

XZ= 6.9207 YZ= 7.3297 ZZ= 164.1660

Eigenvalues: 143.2793 166.9194 176.0161

114 C Isotropic = 77.3781 Anisotropy = 126.1790

XX= 30.5728 YX= 17.0343 ZX= -21.5112

XY= 14.1422 YY= 76.4096 ZY= 60.7822

XZ= -18.7085 YZ= 49.2305 ZZ= 125.1518

Eigenvalues: 10.7614 59.8754 161.4974

115 C Isotropic = 79.6435 Anisotropy = 89.8909

XX= 21.1790 YX= 16.6736 ZX= -16.6515

XY= 26.0299 YY= 95.7552 ZY= 29.1555

XZ= -15.3480 YZ= 26.2521 ZZ= 121.9964

Eigenvalues: 10.6715 88.6883 139.5708

116 C Isotropic = 67.8370 Anisotropy = 160.4101

XX= 19.4228 YX= 1.8841 ZX= -14.5797

XY= 4.4706 YY= 56.6377 ZY= 78.1311

XZ= -16.7478 YZ= 69.5003 ZZ= 127.4506

Eigenvalues: 2.5921 26.1419 174.7771

117 C Isotropic = 60.0978 Anisotropy = 141.7272

XX= 6.6717 YX= -28.0296 ZX= 8.4849

XY= -30.7585 YY= 61.5972 ZY= 58.7405

XZ= 1.5386 YZ= 64.8062 ZZ= 112.0245

Eigenvalues: -15.1807 40.8916 154.5826

118 C Isotropic = 76.7659 Anisotropy = 163.4975

XX= 60.0647 YX= -17.6965 ZX= 0.0971

XY= -22.3587 YY= 42.9328 ZY= 91.4233

XZ= -1.8390 YZ= 88.9988 ZZ= 127.3003

Eigenvalues: -17.9587 62.4922 185.7643

119 N Isotropic = 135.7160 Anisotropy = 72.7988

XX= 136.4976 YX= -23.3058 ZX= 15.8478

XY= -13.3712 YY= 118.6729 ZY= 46.0242

XZ= 14.3801 YZ= 45.8070 ZZ= 151.9775

Eigenvalues: 77.0379 145.8616 184.2485

120 C Isotropic = 86.0620 Anisotropy = 143.9134

XX= 71.2379 YX= -0.7089 ZX= -8.6396

XY= 1.2126 YY= 58.0194 ZY= 80.8012

XZ= -11.4575 YZ= 80.0860 ZZ= 128.9289

Eigenvalues: 5.0700 71.1118 182.0043

121 C Isotropic = 73.6699 Anisotropy = 162.6835

XX= 1.5378 YX= -40.3676 ZX= 11.5995

XY= -39.9195 YY= 81.0823 ZY= 64.1344

XZ= 10.6563 YZ= 66.7616 ZZ= 138.3897

Eigenvalues: -24.3771 63.2613 182.1255

122 C Isotropic = 70.1894 Anisotropy = 172.5857

XX= 3.1045 YX= 23.2737 ZX= -29.7772

XY= 25.1425 YY= 74.1591 ZY= 74.9345

XZ= -31.7894 YZ= 75.2592 ZZ= 133.3045

Eigenvalues: -25.9966 51.3182 185.2465

123 H Isotropic = 23.9069 Anisotropy = 3.2522

XX= 24.7527 YX= 1.4893 ZX= 1.1187

XY= 1.4771 YY= 24.3627 ZY= -0.8019

XZ= 0.8116 YZ= -0.6290 ZZ= 22.6053

Eigenvalues: 21.6286 24.0171 26.0750

124 H Isotropic = 24.8772 Anisotropy = 6.6879

XX= 19.2965 YX= -2.3382 ZX= 0.3908

XY= -1.9235 YY= 26.1515 ZY= 1.9626

XZ= -0.9013 YZ= -0.8088 ZZ= 29.1835

Eigenvalues: 18.6874 26.6083 29.3357

125 H Isotropic = 28.4456 Anisotropy = 4.4917

XX= 27.5778 YX= -0.2975 ZX= -1.5337

XY= -1.4587 YY= 29.8805 ZY= -2.6218

XZ= -5.6359 YZ= -0.1372 ZZ= 27.8784

Eigenvalues: 23.7286 30.1681 31.4400

126 H Isotropic = 27.9758 Anisotropy = 6.1431

XX= 30.4592 YX= 0.1759 ZX= 4.8333

XY= -0.4088 YY= 29.4483 ZY= -0.2299

XZ= 2.1574 YZ= 2.9573 ZZ= 24.0198

Eigenvalues: 22.2533 29.6028 32.0712

127 H Isotropic = 28.5361 Anisotropy = 6.5796

XX= 27.5766 YX= -0.4752 ZX= -1.9465

XY= -0.2317 YY= 27.8952 ZY= -0.5000

XZ= -4.3008 YZ= -4.2981 ZZ= 30.1367

Eigenvalues: 24.5669 28.1189 32.9225

128 H Isotropic = 24.3098 Anisotropy = 6.9504

XX= 25.8711 YX= 0.9942 ZX= 1.0119

XY= 3.2316 YY= 26.4098 ZY= -2.6089

XZ= -2.8319 YZ= -2.0291 ZZ= 20.6485

Eigenvalues: 19.8265 24.1595 28.9434

129 H Isotropic = 23.7046 Anisotropy = 8.5947

XX= 28.5885 YX= -0.7017 ZX= 2.9056

XY= -0.4340 YY= 21.9428 ZY= -0.7402

XZ= 2.2819 YZ= -0.8326 ZZ= 20.5824

Eigenvalues: 19.6574 22.0219 29.4343

130 H Isotropic = 23.2776 Anisotropy = 7.0918

XX= 25.7918 YX= 2.5111 ZX= 0.6957

XY= 0.8634 YY= 24.1249 ZY= -5.1034

XZ= -0.0452 YZ= -4.5281 ZZ= 19.9162

Eigenvalues: 16.6070 25.2204 28.0055

131 H Isotropic = 23.8542 Anisotropy = 6.1852

XX= 27.5445 YX= 0.3988 ZX= 1.6463

XY= -0.1529 YY= 22.5315 ZY= -0.6622

XZ= 1.7067 YZ= -0.0911 ZZ= 21.4866

Eigenvalues: 20.9544 22.6306 27.9776

132 H Isotropic = 24.2503 Anisotropy = 5.1404

XX= 25.5884 YX= -0.6802 ZX= 2.9496

XY= -0.5742 YY= 24.8246 ZY= -1.0718

XZ= 2.5653 YZ= -1.5279 ZZ= 22.3377

Eigenvalues: 20.5958 24.4778 27.6772

133 H Isotropic = 29.8445 Anisotropy = 4.4442

XX= 29.8943 YX= 1.4557 ZX= 3.9136

XY= 0.1612 YY= 31.1805 ZY= 0.5156

XZ= 3.0054 YZ= -1.2757 ZZ= 28.4587

Eigenvalues: 25.5282 31.1980 32.8073

134 O Isotropic = 290.3029 Anisotropy = 65.8462

XX= 268.6956 YX= 9.9167 ZX= 15.7011

XY= 7.9219 YY= 278.7024 ZY= 16.1374

XZ= 14.3006 YZ= 19.4663 ZZ= 323.5106

Eigenvalues: 263.0305 273.6778 334.2003

135 H Isotropic = 25.1761 Anisotropy = 26.5981

XX= 17.3521 YX= -1.6827 ZX= -0.0994

XY= -1.4042 YY= 15.6382 ZY= 2.6354

XZ= 0.8923 YZ= 3.7035 ZZ= 42.5379

Eigenvalues: 14.4193 18.2008 42.9082

136 H Isotropic = 29.1396 Anisotropy = 22.1986

XX= 27.8377 YX= 8.6878 ZX= 4.6767

XY= 9.1447 YY= 33.6272 ZY= 7.2869

XZ= 4.4290 YZ= 6.6157 ZZ= 25.9538

Eigenvalues: 21.2099 22.2702 43.9386

137 O Isotropic = 300.6580 Anisotropy = 36.9291

XX= 292.4331 YX= 7.4140 ZX= -12.0128

XY= 7.3652 YY= 295.9855 ZY= -7.2393

XZ= -17.0132 YZ= -10.4530 ZZ= 313.5554

Eigenvalues: 284.2478 292.4488 325.2774

138 H Isotropic = 29.6020 Anisotropy = 19.8515

XX= 33.7018 YX= 7.3183 ZX= -4.8010

XY= 9.0895 YY= 31.6023 ZY= -3.3388

XZ= -5.0452 YZ= -3.8789 ZZ= 23.5017

Eigenvalues: 21.4964 24.4731 42.8363

139 H Isotropic = 25.4169 Anisotropy = 25.5116

XX= 17.2867 YX= -0.8884 ZX= 0.8744

XY= 2.8322 YY= 16.5507 ZY= 0.7155

XZ= -1.4513 YZ= 0.2244 ZZ= 42.4132

Eigenvalues: 15.8680 17.9579 42.4246

140 C Isotropic = 145.0809 Anisotropy = 46.7408

XX= 149.8029 YX= 10.4576 ZX= 3.9844

XY= 20.0979 YY= 162.1754 ZY= 11.8949

XZ= 8.7064 YZ= 13.6045 ZZ= 123.2644

Eigenvalues: 119.3403 139.6610 176.2414

141 C Isotropic = 25.1912 Anisotropy = 132.0692

XX= 30.9056 YX= -69.5150 ZX= 17.3680

XY= -76.0136 YY= 38.8010 ZY= -19.5147

XZ= 20.9846 YZ= -11.1546 ZZ= 5.8670

Eigenvalues: -38.3022 0.6385 113.2373

142 O Isotropic = -33.1262 Anisotropy = 503.5873

XX= 47.2202 YX= -192.9493 ZX= 124.5612

XY= -198.6659 YY= 90.1041 ZY= -85.2976

XZ= 112.3642 YZ= -80.6350 ZZ= -236.7031

Eigenvalues: -280.1497 -121.8276 302.5986

143 C Isotropic = 156.4606 Anisotropy = 21.6062

XX= 152.6818 YX= 10.4111 ZX= -3.7603

XY= 8.7976 YY= 165.2895 ZY= 5.1912

XZ= -6.1667 YZ= 6.0819 ZZ= 151.4105

Eigenvalues: 142.0539 156.4632 170.8647

144 C Isotropic = 159.6697 Anisotropy = 28.3883

XX= 161.5971 YX= 10.0955 ZX= 0.7344

XY= 10.3891 YY= 171.2001 ZY= 6.9251

XZ= 0.5459 YZ= 4.8889 ZZ= 146.2120

Eigenvalues: 144.6878 155.7261 178.5953

145 C Isotropic = 173.5943 Anisotropy = 20.5515

XX= 173.3470 YX= 0.9068 ZX= 8.5954

XY= -0.6120 YY= 168.4061 ZY= -2.0910

XZ= 12.2368 YZ= -4.1960 ZZ= 179.0297

Eigenvalues: 164.3207 169.1669 187.2953

146 C Isotropic = 175.3063 Anisotropy = 23.1130

XX= 169.3277 YX= -5.9324 ZX= -9.7104

XY= 0.2862 YY= 182.9811 ZY= 8.6476

XZ= -8.2807 YZ= 8.7828 ZZ= 173.6100

Eigenvalues: 161.6407 173.5631 190.7149

147 H Isotropic = 30.4093 Anisotropy = 11.6911

XX= 30.9552 YX= 4.1171 ZX= 1.0503

XY= 5.2286 YY= 34.2649 ZY= 2.6015

XZ= 0.9923 YZ= 2.7628 ZZ= 26.0079

Eigenvalues: 25.1782 27.8463 38.2034

148 H   Isotropic =   29.1939   Anisotropy =   5.1955

XX=   28.4398   YX=   -0.5688   ZX=   4.3394

XY=   -0.9562   YY=   30.1308   ZY=   -0.3638

XZ=   2.4623   YZ=   -1.3460   ZZ=   29.0111

Eigenvalues: 25.3126   29.6116   32.6576

149 H   Isotropic =   29.9708   Anisotropy =   5.0700

XX=   31.5704   YX=   -0.7377   ZX=   -1.9551

XY=   -0.3949   YY=   30.6552   ZY=   2.6695

XZ=   0.1872   YZ=   4.0191   ZZ=   27.6869

Eigenvalues: 25.4809   31.0807   33.3508

150 H   Isotropic =   30.3710   Anisotropy =   4.3439

XX=   27.8197   YX=   0.1670   ZX=   -1.2632

XY=   0.1173   YY=   30.3436   ZY=   0.7300

XZ=   -0.9457   YZ=   0.3710   ZZ=   32.9498

Eigenvalues: 27.5690   30.2771   33.2669

151 H   Isotropic =   30.4651   Anisotropy =   8.6472

XX=   34.3496   YX=   -0.4023   ZX=   4.5287

XY=   -0.0602   YY=   30.9298   ZY=   2.3178

XZ=   3.8632   YZ=   2.6923   ZZ=   26.1159

Eigenvalues: 23.5677   31.5978   36.2299

152 H   Isotropic =   30.7957   Anisotropy =   7.7390

XX=   34.3549   YX=   2.3589   ZX=   2.2828

XY=   1.8898   YY=   31.5446   ZY=   -0.2328

XZ=   2.6400   YZ=   -0.2919   ZZ=   26.4875

Eigenvalues: 25.6526   30.7794   35.9550

153 H   Isotropic =   30.4714   Anisotropy =   9.6426

XX=   33.3536   YX=   -3.6408   ZX=   3.2104

XY=   -4.9100   YY=   31.4813   ZY=   1.7507

XZ=   2.3688   YZ=   2.0463   ZZ=   26.5792

Eigenvalues: 23.9542   30.5602   36.8998

154 H   Isotropic =   30.3066   Anisotropy =   3.9744

XX=   29.4065   YX=   2.4309   ZX=   -1.7210

XY=   2.3083   YY=   29.1711   ZY=   1.1152

XZ=   -1.2173   YZ=   1.0759   ZZ=   32.3422

Eigenvalues: 26.3684   31.5951   32.9562

155 H   Isotropic =   30.9532   Anisotropy =   9.4335

XX=   31.9196   YX=   -4.5694   ZX=   3.4509

XY=   -4.4199   YY=   33.0591   ZY=   1.8425

XZ=   3.5320   YZ=   0.7433   ZZ=   27.8808

Eigenvalues: 24.3730   31.2443   37.2422

156 H   Isotropic =   31.1662   Anisotropy =   7.9418

XX=   28.8003   YX=   -0.0164   ZX=   -0.3611

XY=   -1.0968   YY=   29.3929   ZY=   3.3416

XZ=   -0.0477   YZ=   2.3139   ZZ=   35.3055

Eigenvalues: 28.0076   29.0303   36.4607

157 H   Isotropic =   30.0614   Anisotropy =   11.4658

XX=   25.5981   YX=   -1.5169   ZX=   0.0990

XY=   -0.6451   YY=   26.9203   ZY=   0.6715

XZ=   -1.0287   YZ=   0.2089   ZZ=   37.6656

Eigenvalues: 24.9892   27.4897   37.7052

158 N   Isotropic =   138.8420   Anisotropy =   89.0087

XX=   194.0571   YX=   3.9137   ZX=   -11.5267

XY=   -11.5208   YY=   150.5740   ZY=   -33.8445

XZ=   -26.2064   YZ=   -84.9302   ZZ=   71.8949

Eigenvalues: 37.8243   180.5205   198.1811

159 C   Isotropic =   144.7051   Anisotropy =   25.8673

XX=   144.4195   YX=   -10.1251   ZX=   10.8718

XY=   -10.0876   YY=   156.1236   ZY=   6.8393

XZ=   11.6740   YZ=   6.1798   ZZ=   133.5721

Eigenvalues: 122.8253   149.3399   161.9499

160 C   Isotropic =   25.5624   Anisotropy =   125.0804

XX=   15.5884   YX=   63.5791   ZX=   -29.1083

XY=   60.8180   YY=   31.9706   ZY=   -22.4275

XZ=   -46.8401   YZ=   -22.6399   ZZ=   29.1282

Eigenvalues: -42.2090 9.9469 108.9493

161 O Isotropic = -31.4669 Anisotropy = 531.5838

XX= 15.3662 YX= 149.2911 ZX= -163.8924

XY= 141.4479 YY= 26.8552 ZY= -171.8310

XZ= -205.3355 YZ= -216.8037 ZZ= -136.6220

Eigenvalues: -292.9601 -124.3628 322.9223

162 H Isotropic = 28.3659 Anisotropy = 4.7333

XX= 31.3489 YX= -0.3547 ZX= -0.9596

XY= 0.5018 YY= 27.0029 ZY= 1.3857

XZ= -0.7991 YZ= 1.6594 ZZ= 26.7459

Eigenvalues: 25.2653 28.3110 31.5214

163 H Isotropic = 21.2510 Anisotropy = 17.1024

XX= 20.1576 YX= -0.7831 ZX= -8.7903

XY= 0.9623 YY= 17.1569 ZY= 4.6012

XZ= -7.2745 YZ= 3.5831 ZZ= 26.4385

Eigenvalues: 13.0584 18.0420 32.6526

164 H Isotropic = 27.1059 Anisotropy = 8.3449

XX= 29.2860 YX= -0.2337 ZX= 5.2715

XY= -1.9253 YY= 25.5956 ZY= 0.0989

XZ= 3.8469 YZ= 1.2614 ZZ= 26.4360

Eigenvalues: 22.6088 26.0397 32.6692

165 N Isotropic = 137.0631 Anisotropy = 108.5590

XX= 186.5620 YX= -18.0760 ZX= -5.3391

XY= -6.8626 YY= 114.8280 ZY= -73.1484

XZ= 37.1450 YZ= -85.9019 ZZ= 109.7992

Eigenvalues: 32.6990 169.0545 209.4358

166 C Isotropic = 127.4005 Anisotropy = 20.6734

XX= 123.7578 YX= 0.8080 ZX= -6.7830

XY= 1.8396 YY= 124.3191 ZY= -5.9731

XZ= -6.7370 YZ= -10.2455 ZZ= 134.1247

Eigenvalues: 118.3329 122.6859 141.1828

167 C Isotropic = 23.6964 Anisotropy = 122.6707

XX=   69.9822   YX=   -45.1571   ZX=   47.3810

XY=   -51.4851   YY=   -7.0858   ZY=   22.0222

XZ=   39.3659   YZ=   5.0432   ZZ=   8.1929

Eigenvalues: -49.5733   15.1857   105.4769

168 O   Isotropic =   -58.8047   Anisotropy =   563.1620

XX=   212.9785   YX= -153.0629   ZX=   116.0974

XY= -134.6679   YY= -210.7109   ZY= -123.7847

XZ=   151.7577   YZ= -154.4023   ZZ= -178.6817

Eigenvalues: -335.5185 -157.5322   316.6366

169 C   Isotropic =   152.4289   Anisotropy =   16.6704

XX=   146.6952   YX=   5.4474   ZX=   1.8148

XY=   5.2136   YY=   150.4757   ZY=   -1.8625

XZ=   12.7599   YZ=   -6.2836   ZZ=   160.1160

Eigenvalues: 139.5669   154.1774   163.5426

170 C   Isotropic =   162.2797   Anisotropy =   19.2583

XX=   161.8077   YX=   6.0815   ZX=   -2.7384

XY=   7.2104   YY=   155.0008   ZY=   -8.9009

XZ=   -4.0859   YZ=   -4.5181   ZZ=   170.0305

Eigenvalues: 150.0429   161.6776   175.1185

171 C   Isotropic =   173.8074   Anisotropy =   23.7996

XX=   175.5525   YX=   -7.1363   ZX=   -5.5674

XY=   -12.1354   YY=   179.9805   ZY=   5.7172

XZ=   -4.1965   YZ=   4.2216   ZZ=   165.8892

Eigenvalues: 163.7537   167.9947   189.6738

172 C   Isotropic =   178.8578   Anisotropy =   25.5258

XX=   175.8910   YX=   3.1396   ZX=   13.7123

XY=   4.6401   YY=   171.7701   ZY=   -1.7510

XZ=   9.8792   YZ=   -2.7193   ZZ=   188.9122

Eigenvalues: 165.6436   175.0548   195.8749

173 H   Isotropic =   30.6287   Anisotropy =   10.9795

XX=   28.1533   YX=   0.7105   ZX=   -0.1134

XY=   1.0887   YY=   26.7190   ZY=   -3.1333

XZ= -1.2373 YZ= -3.0349 ZZ= 37.0138

Eigenvalues: 25.6706 28.2672 37.9484

174 H Isotropic = 25.7764 Anisotropy = 9.5172

XX= 24.7656 YX= -5.8717 ZX= 3.3385

XY= -4.3380 YY= 27.7122 ZY= -1.3478

XZ= 2.4094 YZ= 0.7220 ZZ= 24.8514

Eigenvalues: 19.9512 25.2567 32.1212

175 H Isotropic = 27.6228 Anisotropy = 3.8807

XX= 27.1855 YX= 1.8812 ZX= 2.7246

XY= 0.7113 YY= 29.2528 ZY= 0.8065

XZ= 2.6285 YZ= -1.5953 ZZ= 26.4302

Eigenvalues: 23.8564 28.8021 30.2100

176 H Isotropic = 30.5245 Anisotropy = 3.8637

XX= 29.2909 YX= 1.1166 ZX= 3.6211

XY= 0.7247 YY= 32.8759 ZY= -1.4800

XZ= 2.5068 YZ= -0.0962 ZZ= 29.4065

Eigenvalues: 26.0694 32.4037 33.1003

177 H Isotropic = 30.3922 Anisotropy = 7.0580

XX= 30.9978 YX= -4.6789 ZX= 0.7150

XY= -4.0955 YY= 27.9391 ZY= -2.3459

XZ= 0.3791 YZ= -1.7218 ZZ= 32.2396

Eigenvalues: 24.5788 31.5003 35.0975

178 H Isotropic = 30.8303 Anisotropy = 6.7357

XX= 33.1862 YX= -1.8850 ZX= -2.6513

XY= -2.1302 YY= 27.2340 ZY= -2.7933

XZ= -2.6063 YZ= -2.7095 ZZ= 32.0707

Eigenvalues: 24.9246 32.2456 35.3208

179 H Isotropic = 30.5210 Anisotropy = 9.8289

XX= 31.5241 YX= -4.8088 ZX= 2.4941

XY= -4.5397 YY= 28.9571 ZY= -1.5098

XZ= 3.6492 YZ= -1.7861 ZZ= 31.0818

Eigenvalues: 25.3262 29.1631 37.0736

180 H   Isotropic =   29.7586   Anisotropy =   13.3098

XX=   26.7438   YX=   7.0855   ZX=   3.0167

XY=   5.8094   YY=   34.6839   ZY=   0.5803

XZ=   2.8869   YZ=   0.4771   ZZ=   27.8482

Eigenvalues: 22.1581   28.4859   38.6318

181 H   Isotropic =   30.4545   Anisotropy =   11.2450

XX=   34.0912   YX=   -3.9705   ZX=   4.0995

XY=   -3.5601   YY=   26.1714   ZY=   -2.2711

XZ=   3.1724   YZ=   -1.1744   ZZ=   31.1009

Eigenvalues: 24.6544   28.7579   37.9511

182 H   Isotropic =   31.1435   Anisotropy =   9.8399

XX=   28.9090   YX=   2.8813   ZX=   4.3973

XY=   1.9265   YY=   31.8853   ZY=   2.0742

XZ=   4.5914   YZ=   3.0316   ZZ=   32.6363

Eigenvalues: 25.8373   29.8898   37.7035

183 H   Isotropic =   30.9603   Anisotropy =   6.8244

XX=   29.6487   YX=   0.2735   ZX=   1.1683

XY=   1.2913   YY=   35.1975   ZY=   0.6188

XZ=   1.2687   YZ=   1.5067   ZZ=   28.0347

Eigenvalues: 27.3368   30.0342   35.5099

184 N   Isotropic =   135.1745   Anisotropy =   103.4866

XX=   171.7548   YX=   32.7532   ZX=   14.0990

XY=   32.5039   YY=   164.8185   ZY=   -72.7614

XZ=   39.8756   YZ=   -35.7142   ZZ=   68.9500

Eigenvalues: 33.8498   167.5081   204.1655

185 C   Isotropic =   136.9478   Anisotropy =   23.0477

XX=   141.2004   YX=   -6.8640   ZX=   4.7711

XY=   -4.2129   YY=   119.9939   ZY=   -1.4472

XZ=   4.8330   YZ=   -2.1097   ZZ=   149.6490

Eigenvalues: 118.6232   139.9072   152.3129

186 C   Isotropic =   25.0466   Anisotropy =   117.0127

XX=   -24.3787   YX=   25.5219   ZX=   -34.6917

XY=   30.3104   YY=   90.5588   ZY=   -16.6616

XZ=   -23.2172   YZ=   -17.9148   ZZ=   8.9596

Eigenvalues: -43.1006   15.1853   103.0550

187 O   Isotropic =   -29.9770   Anisotropy =   516.7408

XX= -129.4180   YX=   117.3109   ZX=   5.6501

XY=   129.9304   YY=   247.3149   ZY= -147.4623

XZ=   38.3338   YZ= -126.2097   ZZ= -207.8279

Eigenvalues: -270.6923 -133.7556   314.5169

188 C   Isotropic =   174.5847   Anisotropy =   28.2457

XX=   167.8787   YX=   8.7329   ZX=   1.2293

XY=   13.0233   YY=   188.0176   ZY=   2.6131

XZ=   1.6624   YZ=   4.9770   ZZ=   167.8580

Eigenvalues: 163.1158   167.2231   193.4152

189 H   Isotropic =   30.6836   Anisotropy =   10.8728

XX=   34.4278   YX=   2.5640   ZX=   4.3391

XY=   2.6972   YY=   28.2280   ZY=   2.1801

XZ=   4.3073   YZ=   1.2817   ZZ=   29.3950

Eigenvalues: 26.8247   27.2940   37.9321

190 H   Isotropic =   26.5167   Anisotropy =   4.8315

XX=   25.3389   YX=   -0.1279   ZX=   -1.7076

XY=   0.4727   YY=   28.9041   ZY=   -1.2638

XZ=   -3.1375   YZ=   -1.7464   ZZ=   25.3071

Eigenvalues: 22.7480   27.0644   29.7377

191 H   Isotropic =   27.8528   Anisotropy =   6.1176

XX=   29.4156   YX=   -2.2129   ZX=   -3.3368

XY=   -2.3352   YY=   27.0981   ZY=   1.0973

XZ=   -0.5727   YZ=   1.4851   ZZ=   27.0447

Eigenvalues: 25.6829   25.9443   31.9312

192 H   Isotropic =   30.0859   Anisotropy =   8.6044

XX=   31.1306   YX=   3.1925   ZX=   -3.0774

XY=   3.7614   YY=   28.9803   ZY=   -2.1266

XZ=   -2.9334   YZ=   -1.4173   ZZ=   30.1467

Eigenvalues: 26.3565 28.0790 35.8221

193 H Isotropic = 29.9766 Anisotropy = 8.5047

XX= 32.7143 YX= -2.1050 ZX= -1.1866

XY= -3.5564 YY= 31.7444 ZY= 2.7429

XZ= -1.3356 YZ= 1.5340 ZZ= 25.4710

Eigenvalues: 24.7908 29.4925 35.6464

194 N Isotropic = 137.3343 Anisotropy = 90.0707

XX= 186.9727 YX= -0.2304 ZX= 21.8285

XY= -23.8217 YY= 178.6491 ZY= -31.5106

XZ= -24.3912 YZ= -25.4763 ZZ= 46.3810

Eigenvalues: 40.4109 174.2106 197.3814

195 C Isotropic = 135.6724 Anisotropy = 15.6885

XX= 124.3962 YX= 3.4495 ZX= 10.3722

XY= 3.9164 YY= 143.1970 ZY= -0.1084

XZ= 8.0184 YZ= 2.0947 ZZ= 139.4238

Eigenvalues: 119.6733 141.2124 146.1313

196 C Isotropic = 26.5505 Anisotropy = 118.5189

XX= 103.6969 YX= -15.0017 ZX= 0.9841

XY= -17.4606 YY= -37.0719 ZY= -4.7011

XZ= 2.1304 YZ= 8.7098 ZZ= 13.0263

Eigenvalues: -39.0094 13.0978 105.5631

197 O Isotropic = -37.8435 Anisotropy = 518.6148

XX= 302.1521 YX= -45.1697 ZX= 40.0852

XY= -52.6553 YY= -129.5910 ZY= 4.9515

XZ= -11.2385 YZ= 22.8064 ZZ= -286.0914

Eigenvalues: -287.9170 -133.5131 307.8997

198 C Isotropic = 159.8756 Anisotropy = 19.0532

XX= 168.1321 YX= -8.7771 ZX= 1.6110

XY= -7.8635 YY= 154.8639 ZY= -0.4255

XZ= 3.4497 YZ= -1.2515 ZZ= 156.6309

Eigenvalues: 150.8351 156.2140 172.5778

199 C Isotropic = 60.6703 Anisotropy = 118.0062

XX= 18.9141 YX= 48.5636 ZX= -31.6350

XY= 46.8570 YY= 118.6356 ZY= -4.3976

XZ= -10.6339 YZ= -4.5042 ZZ= 44.4611

Eigenvalues: -6.6665 49.3362 139.3411

200 C Isotropic = 81.3017 Anisotropy = 109.4552

XX= 32.5944 YX= 52.2670 ZX= 3.3789

XY= 40.8619 YY= 135.5688 ZY= -11.2607

XZ= 4.4551 YZ= -8.3823 ZZ= 75.7420

Eigenvalues: 13.8217 75.8116 154.2718

201 N Isotropic = 8.1497 Anisotropy = 312.6673

XX= -79.3302 YX= 127.4959 ZX= -31.0860

XY= 132.2078 YY= 156.3006 ZY= -19.2962

XZ= -46.1050 YZ= -6.0071 ZZ= -52.5214

Eigenvalues: -146.7229 -45.4227 216.5946

202 C Isotropic = 69.4293 Anisotropy = 105.9669

XX= 33.1680 YX= 47.6226 ZX= 18.9892

XY= 54.0923 YY= 115.6536 ZY= -13.0041

XZ= 0.9012 YZ= -4.9505 ZZ= 59.4662

Eigenvalues: 5.8510 62.3629 140.0739

203 N Isotropic = 107.7062 Anisotropy = 126.9836

XX= 113.9627 YX= 33.7101 ZX= -29.9083

XY= 34.4692 YY= 177.1055 ZY= 4.4998

XZ= -34.7527 YZ= 7.6517 ZZ= 32.0503

Eigenvalues: 18.9139 111.8428 192.3619

204 H Isotropic = 24.0398 Anisotropy = 12.1198

XX= 21.6291 YX= -2.1981 ZX= -0.4933

XY= 0.3454 YY= 19.0015 ZY= -4.9879

XZ= -0.2413 YZ= -0.7540 ZZ= 31.4887

Eigenvalues: 18.0989 21.9007 32.1196

205 H Isotropic = 22.2675 Anisotropy = 10.5900

XX= 21.9788 YX= -3.9844 ZX= -0.8301

XY= -2.4174 YY= 18.8874 ZY= 7.5664

XZ= 1.0433 YZ= 3.6012 ZZ= 25.9363

Eigenvalues: 14.6708 22.8042 29.3275

206 H Isotropic = 27.4275 Anisotropy = 4.7559

XX= 26.5287 YX= -0.7690 ZX= 0.3804

XY= -1.2432 YY= 26.2419 ZY= -3.4945

XZ= 0.7277 YZ= -0.3067 ZZ= 29.5119

Eigenvalues: 25.0230 26.6614 30.5981

207 H Isotropic = 28.5454 Anisotropy = 6.5431

XX= 28.3104 YX= 0.0850 ZX= -2.2023

XY= -1.3022 YY= 27.8333 ZY= -4.7916

XZ= -0.6765 YZ= -3.2841 ZZ= 29.4927

Eigenvalues: 24.0827 28.6461 32.9075

208 H Isotropic = 25.0587 Anisotropy = 7.5217

XX= 24.1178 YX= -4.3584 ZX= 2.3844

XY= -3.8544 YY= 22.6780 ZY= -2.0447

XZ= 0.9363 YZ= -0.4040 ZZ= 28.3804

Eigenvalues: 19.2269 25.8761 30.0732

209 H Isotropic = 26.7250 Anisotropy = 10.8377

XX= 32.3577 YX= -4.0189 ZX= -1.2897

XY= -3.9970 YY= 22.8791 ZY= 2.1027

XZ= 0.5606 YZ= 1.9903 ZZ= 24.9382

Eigenvalues: 20.6451 25.5797 33.9502

210 H Isotropic = 28.2822 Anisotropy = 6.3155

XX= 27.3708 YX= -2.1946 ZX= 2.7801

XY= -2.1500 YY= 29.2773 ZY= 4.1966

XZ= 2.0485 YZ= 3.2326 ZZ= 28.1985

Eigenvalues: 22.7180 29.6360 32.4925

211 N Isotropic = 138.4855 Anisotropy = 94.0869

XX= 176.9048 YX= 9.9437 ZX= -15.5436

XY= -3.8981 YY= 174.3660 ZY= -35.9743

XZ= -14.6863 YZ= -76.5068 ZZ= 64.1856

Eigenvalues: 39.3605 174.8859 201.2100

212 C Isotropic = 133.7369 Anisotropy = 20.8691

XX= 141.4583 YX= -4.6692 ZX= -5.4135

XY= -2.9843 YY= 131.8816 ZY= 11.5420

XZ= -6.8241 YZ= 8.0267 ZZ= 127.8708

Eigenvalues: 119.6125 133.9486 147.6496

213 C Isotropic = 26.5016 Anisotropy = 115.4566

XX= -44.8919 YX= 9.3359 ZX= 11.7371

XY= 1.7682 YY= 87.0659 ZY= -31.1267

XZ= -0.3770 YZ= -34.6562 ZZ= 37.3308

Eigenvalues: -45.7877 21.8198 103.4727

214 O Isotropic = -58.3482 Anisotropy = 575.3210

XX= -168.2101 YX= -6.8432 ZX= -46.6196

XY= -20.7259 YY= 193.6766 ZY= -238.7719

XZ= -54.5695 YZ= -286.5872 ZZ= -200.5110

Eigenvalues: -346.6472 -153.5964 325.1991

215 C Isotropic = 151.6550 Anisotropy = 29.8200

XX= 141.8098 YX= 8.2417 ZX= -5.8010

XY= 13.0018 YY= 167.7121 ZY= 4.6444

XZ= -6.0603 YZ= 1.2464 ZZ= 145.4431

Eigenvalues: 134.1697 149.2603 171.5350

216 C Isotropic = 164.9741 Anisotropy = 3.4907

XX= 162.1368 YX= 2.3791 ZX= -0.9149

XY= 2.2870 YY= 165.5196 ZY= -1.2629

XZ= 1.0227 YZ= 1.5326 ZZ= 167.2658

Eigenvalues: 160.9465 166.6745 167.3012

217 C Isotropic = 168.6149 Anisotropy = 31.1664

XX= 167.3804 YX= -1.4219 ZX= 18.8827

XY= 2.6752 YY= 161.7370 ZY= -0.6932

XZ= 14.4846 YZ= 1.2347 ZZ= 176.7272

Eigenvalues: 154.7119 161.7403 189.3924

218 C Isotropic = 158.9486 Anisotropy = 32.7425

XX= 148.8844 YX= 1.8648 ZX= -5.6372

XY= -1.5949 YY= 150.1661 ZY= -7.9855

XZ= -1.8315 YZ= -9.6330 ZZ= 177.7954

Eigenvalues: 147.0241 149.0449 180.7770

219 H Isotropic = 30.7158 Anisotropy = 7.5784

XX= 32.5067 YX= -0.2694 ZX= -2.9965

XY= 1.0449 YY= 27.0075 ZY= -0.9570

XZ= -3.3087 YZ= -0.0389 ZZ= 32.6333

Eigenvalues: 26.9606 29.4189 35.7681

220 H Isotropic = 24.9863 Anisotropy = 4.7536

XX= 21.0972 YX= 0.3764 ZX= 0.7188

XY= 1.0708 YY= 26.2626 ZY= -0.5424

XZ= 2.1384 YZ= 1.6435 ZZ= 27.5991

Eigenvalues: 20.7323 26.0711 28.1554

221 H Isotropic = 28.5635 Anisotropy = 3.4057

XX= 28.7623 YX= 2.1776 ZX= 2.7737

XY= -0.9099 YY= 29.1653 ZY= -0.1573

XZ= 2.2508 YZ= -0.9993 ZZ= 27.7628

Eigenvalues: 25.5040 29.3525 30.8339

222 H Isotropic = 30.0418 Anisotropy = 5.7585

XX= 28.9515 YX= 3.3071 ZX= 2.6148

XY= 3.3289 YY= 29.2367 ZY= -1.0402

XZ= 2.8615 YZ= -0.6278 ZZ= 31.9373

Eigenvalues: 24.8263 31.4184 33.8808

223 H Isotropic = 30.1860 Anisotropy = 7.2712

XX= 33.4377 YX= -2.4731 ZX= -2.5287

XY= -1.9396 YY= 29.8636 ZY= -0.8389

XZ= -2.6581 YZ= -0.8377 ZZ= 27.2567

Eigenvalues: 25.6881 29.8365 35.0335

224 H Isotropic = 31.2614 Anisotropy = 7.7963

XX= 28.9736 YX= 2.0430 ZX= 1.0641

XY= 1.5006 YY= 33.7686 ZY= 2.8562

XZ= 2.5430 YZ= 3.0175 ZZ= 31.0420

Eigenvalues: 27.9275   29.3977   36.4589

225 H   Isotropic =   30.5147   Anisotropy =   6.7603

XX=   27.9340   YX=   0.9484   ZX=   0.1132

XY=   1.3276   YY=   34.8135   ZY=   -0.1336

XZ=   -0.0441   YZ=   -0.6713   ZZ=   28.7965

Eigenvalues: 27.7413   28.7811   35.0215

226 H   Isotropic =   31.1347   Anisotropy =   4.2323

XX=   32.1573   YX=   0.2949   ZX=   -1.9760

XY=   -0.2915   YY=   30.9407   ZY=   -1.2033

XZ=   -2.9188   YZ=   -0.7585   ZZ=   30.3060

Eigenvalues: 28.3579   31.0899   33.9562

227 H   Isotropic =   32.1246   Anisotropy =   10.3475

XX=   37.5596   YX=   -4.6213   ZX=   0.3104

XY=   -2.1056   YY=   29.9390   ZY=   -0.5475

XZ=   2.0070   YZ=   -1.1933   ZZ=   28.8752

Eigenvalues: 28.2749   29.0760   39.0229

228 H   Isotropic =   30.3690   Anisotropy =   9.0824

XX=   27.7662   YX=   -1.2990   ZX=   1.9251

XY=   -1.8639   YY=   28.4476   ZY=   -2.7552

XZ=   2.6784   YZ=   -1.7190   ZZ=   34.8932

Eigenvalues: 26.4612   28.2218   36.4239

229 H   Isotropic =   29.4798   Anisotropy =   8.2351

XX=   27.3642   YX=   2.0972   ZX=   -0.6667

XY=   3.7586   YY=   27.5402   ZY=   -1.6899

XZ=   -0.4629   YZ=   -3.8012   ZZ=   33.5350

Eigenvalues: 24.2519   29.2177   34.9699

230 N   Isotropic =   139.6656   Anisotropy =   97.3013

XX=   194.5672   YX=   9.7986   ZX=   12.4810

XY=   18.6335   YY=   141.5206   ZY=   -58.5995

XZ=   57.0199   YZ=   -49.3349   ZZ=   82.9089

Eigenvalues: 41.7660   172.6976   204.5331

231 C   Isotropic =   135.8579   Anisotropy =   21.4167

XX=   125.5309   YX=   -7.0557   ZX=   -11.0726

XY=   -5.3233   YY=   138.4275   ZY=   -6.3072

XZ=   -10.4671   YZ=   -7.6752   ZZ=   143.6155

Eigenvalues: 117.0144   140.4237   150.1357

232 C   Isotropic =   26.5954   Anisotropy =   114.3444

XX=   97.7554   YX=   23.1555   ZX=   -1.7823

XY=   23.3517   YY=   -20.1796   ZY=   45.0594

XZ=   3.2950   YZ=   29.0558   ZZ=   2.2102

Eigenvalues: -49.9836   26.9447   102.8250

233 O   Isotropic =   -63.0502   Anisotropy =   576.6180

XX=   277.9512   YX=   130.2121   ZX=   63.9405

XY=   142.0343   YY= -205.5772   ZY=   -54.5878

XZ=   108.4457   YZ=   -79.9375   ZZ= -261.5245

Eigenvalues: -342.7064 -167.8059   321.3619

234 C   Isotropic =   174.5543   Anisotropy =   24.6104

XX=   184.9186   YX=   1.4220   ZX=   8.4389

XY=   5.0562   YY=   164.5184   ZY=   3.3276

XZ=   9.8596   YZ=   3.7406   ZZ=   174.2257

Eigenvalues: 163.3636   169.3380   190.9612

235 H   Isotropic =   30.7380   Anisotropy =   8.0543

XX=   30.9603   YX=   -2.7437   ZX=   2.0508

XY=   -2.7660   YY=   27.7670   ZY=   -2.2336

XZ=   1.9305   YZ=   -2.7654   ZZ=   33.4867

Eigenvalues: 25.9577   30.1488   36.1076

236 H   Isotropic =   24.8259   Anisotropy =   4.7176

XX=   27.4527   YX=   -0.3873   ZX=   0.2493

XY=   -0.2284   YY=   21.5858   ZY=   2.9610

XZ=   -0.7006   YZ=   4.5410   ZZ=   25.4391

Eigenvalues: 19.2930   27.2137   27.9709

237 H   Isotropic =   27.6523   Anisotropy =   6.9990

XX=   28.3280   YX=   -1.9557   ZX=   -2.4330

XY=   -2.5824   YY=   29.7413   ZY=   3.4940

XZ= -0.4544 YZ= 0.8269 ZZ= 24.8876

Eigenvalues: 23.9762 26.6625 32.3183

238 H Isotropic = 30.4210 Anisotropy = 12.2346

XX= 37.4038 YX= -3.3472 ZX= -2.1941

XY= -2.7925 YY= 25.9139 ZY= 1.0624

XZ= -1.5501 YZ= 1.0458 ZZ= 27.9451

Eigenvalues: 25.0211 27.6645 38.5773

239 H Isotropic = 29.9053 Anisotropy = 5.6109

XX= 30.2865 YX= -0.4782 ZX= 1.0113

XY= -2.3060 YY= 32.2641 ZY= 2.4450

XZ= 0.7417 YZ= 2.7679 ZZ= 27.1653

Eigenvalues: 25.6656 30.4045 33.6459

240 N Isotropic = 141.8402 Anisotropy = 94.8043

XX= 170.7307 YX= 6.7596 ZX= 29.8243

XY= 3.9990 YY= 196.8839 ZY= -56.5165

XZ= 21.8345 YZ= -12.7113 ZZ= 57.9061

Eigenvalues: 44.2618 176.2158 205.0431

241 C Isotropic = 136.9480 Anisotropy = 25.1810

XX= 145.0966 YX= 4.7309 ZX= 6.3779

XY= 8.1301 YY= 116.9275 ZY= 0.0583

XZ= 6.1659 YZ= -2.5874 ZZ= 148.8200

Eigenvalues: 115.3253 141.7834 153.7354

242 C Isotropic = 26.5446 Anisotropy = 117.5802

XX= -23.6769 YX= -51.4268 ZX= -15.1230

XY= -45.7332 YY= 82.8230 ZY= -23.8837

XZ= -3.0240 YZ= -18.4637 ZZ= 20.4877

Eigenvalues: -46.4921 21.1946 104.9314

243 O Isotropic = -25.0263 Anisotropy = 540.8228

XX= -84.4557 YX= -153.2795 ZX= 65.4003

XY= -139.3929 YY= 263.3941 ZY= -117.9756

XZ= 77.3131 YZ= -53.2594 ZZ= -254.0174

Eigenvalues: -282.2260 -128.3752 335.5222

244 C Isotropic = 172.2570 Anisotropy = 21.7280

XX= 164.9474 YX= -6.6968 ZX= 0.2374

XY= -3.3792 YY= 185.5576 ZY= -0.4943

XZ= -0.7544 YZ= 1.6579 ZZ= 166.2659

Eigenvalues: 163.7759 166.2527 186.7423

245 H Isotropic = 30.4169 Anisotropy = 6.4945

XX= 31.2104 YX= 3.1563 ZX= 1.0167

XY= 2.9044 YY= 30.3163 ZY= 1.6114

XZ= 2.1066 YZ= 1.3489 ZZ= 29.7238

Eigenvalues: 27.6924 28.8116 34.7465

246 H Isotropic = 23.5708 Anisotropy = 8.0108

XX= 19.6979 YX= -2.3038 ZX= -1.5685

XY= -2.8443 YY= 23.0429 ZY= 2.3699

XZ= -2.1689 YZ= -0.0159 ZZ= 27.9717

Eigenvalues: 18.1738 23.6274 28.9113

247 H Isotropic = 27.6588 Anisotropy = 8.1327

XX= 30.1956 YX= -1.1544 ZX= -5.2640

XY= -2.0329 YY= 24.7406 ZY= -1.8170

XZ= -2.2262 YZ= -0.0108 ZZ= 28.0400

Eigenvalues: 23.2443 26.6514 33.0806

248 H Isotropic = 29.9888 Anisotropy = 7.0685

XX= 28.0922 YX= -0.1494 ZX= -1.1041

XY= -0.6967 YY= 30.9158 ZY= -4.1519

XZ= -0.0295 YZ= -3.3729 ZZ= 30.9582

Eigenvalues: 26.7964 28.4688 34.7011

249 H Isotropic = 30.2815 Anisotropy = 7.0056

XX= 32.4031 YX= -1.5354 ZX= -3.7694

XY= -4.2794 YY= 29.0482 ZY= -0.0650

XZ= -1.4707 YZ= -0.4673 ZZ= 29.3931

Eigenvalues: 26.3858 29.5067 34.9518

250 N Isotropic = 148.1708 Anisotropy = 102.3360

XX= 208.9569 YX= 5.9725 ZX= -12.5549

XY=   -18.2418   YY=   172.8663   ZY=   -16.2316

XZ=   -54.9692   YZ=   -31.1056   ZZ=   62.6891

Eigenvalues: 50.3972   177.7203   216.3948

251 C   Isotropic =   146.8285   Anisotropy =   36.6189

XX=   166.9173   YX=   6.7362   ZX=   18.7030

XY=   0.8865   YY=   136.5388   ZY=   2.4258

XZ=   4.6658   YZ=   -4.8430   ZZ=   137.0294

Eigenvalues: 131.7482   137.4961   171.2411

252 C   Isotropic =   164.4389   Anisotropy =   17.7174

XX=   175.0900   YX=   4.4647   ZX=   5.0610

XY=   5.5553   YY=   150.4805   ZY=   4.7339

XZ=   -4.5448   YZ=   4.8657   ZZ=   167.7461

Eigenvalues: 148.3754   168.6908   176.2504

253 C   Isotropic =   78.4108   Anisotropy =   124.1563

XX=   70.5210   YX=   -15.0429   ZX=   -57.0432

XY=   -22.2182   YY=   38.9035   ZY=   -12.2787

XZ=   -56.1222   YZ=   -16.2913   ZZ=   125.8080

Eigenvalues: 13.5887   60.4621   161.1817

254 C   Isotropic =   80.3427   Anisotropy =   92.6440

XX=   85.3784   YX=   -30.7953   ZX=   -31.5028

XY=   -24.6129   YY=   29.4765   ZY=   -12.5734

XZ=   -28.4244   YZ=   -23.1540   ZZ=   126.1732

Eigenvalues: 10.9900   87.9328   142.1054

255 C   Isotropic =   70.1196   Anisotropy =   158.3978

XX=   50.7128   YX=   -6.8836   ZX=   -75.5161

XY=   -6.3484   YY=   25.2805   ZY=   0.2228

XZ=   -68.2694   YZ=   -5.9586   ZZ=   134.3656

Eigenvalues: 6.6139   28.0268   175.7182

256 C   Isotropic =   62.1015   Anisotropy =   139.9748

XX=   63.3187   YX=   15.8322   ZX=   -55.4329

XY=   18.6154   YY=   -1.3880   ZY=   13.0035

XZ=   -51.2488   YZ=   16.5775   ZZ=   124.3738

Eigenvalues: -12.5712 43.4576 155.4180

257 C Isotropic = 78.1016 Anisotropy = 161.7533

XX= 45.2565 YX= 23.9158 ZX= -86.3114

XY= 21.4830 YY= 53.5257 ZY= 14.7594

XZ= -82.1169 YZ= 11.3447 ZZ= 135.5228

Eigenvalues: -15.1384 63.5062 185.9372

258 N Isotropic = 134.6795 Anisotropy = 77.5456

XX= 119.0904 YX= 14.5618 ZX= -33.4656

XY= 23.8023 YY= 120.7960 ZY= 13.6829

XZ= -43.4912 YZ= 15.5338 ZZ= 164.1520

Eigenvalues: 82.1908 135.4711 186.3766

259 C Isotropic = 87.1334 Anisotropy = 142.1841

XX= 54.4099 YX= 1.8896 ZX= -77.0889

XY= 3.0637 YY= 73.6466 ZY= 4.8012

XZ= -80.2596 YZ= 2.3038 ZZ= 133.3437

Eigenvalues: 5.6260 73.8513 181.9228

260 C Isotropic = 74.4865 Anisotropy = 163.2679

XX= 88.1637 YX= 29.3500 ZX= -58.0155

XY= 25.6968 YY= -9.2109 ZY= 17.7841

XZ= -63.5210 YZ= 21.4297 ZZ= 144.5067

Eigenvalues: -24.6223 64.7500 183.3318

261 C Isotropic = 72.1865 Anisotropy = 172.1920

XX= 58.9396 YX= -36.4746 ZX= -75.7972

XY= -33.4203 YY= 15.8307 ZY= -15.6304

XZ= -76.2458 YZ= -19.8483 ZZ= 141.7893

Eigenvalues: -24.3250 53.9033 186.9812

262 H Isotropic = 23.9273 Anisotropy = 2.3495

XX= 24.0825 YX= -0.4206 ZX= 0.6493

XY= -0.6250 YY= 25.2335 ZY= -0.2283

XZ= 0.0610 YZ= -0.3926 ZZ= 22.4659

Eigenvalues: 22.3769 23.9114 25.4937

263 H Isotropic = 24.5019 Anisotropy = 7.4093

XX= 26.3295 YX= 1.3825 ZX= -2.2907

XY= 1.7231 YY= 18.0002 ZY= -0.1194

XZ= 0.9060 YZ= -1.3711 ZZ= 29.1758

Eigenvalues: 17.6875 26.3767 29.4414

264 H Isotropic = 28.4343 Anisotropy = 5.8507

XX= 29.7656 YX= 0.9523 ZX= 2.5179

XY= -0.7930 YY= 28.1766 ZY= -2.2333

XZ= 0.1170 YZ= -6.3013 ZZ= 27.3607

Eigenvalues: 23.3159 29.6523 32.3347

265 H Isotropic = 28.0955 Anisotropy = 5.7802

XX= 29.3790 YX= 0.4553 ZX= 0.3644

XY= -0.3710 YY= 30.1243 ZY= 4.4445

XZ= -2.4613 YZ= 2.6012 ZZ= 24.7832

Eigenvalues: 22.8912 29.4463 31.9489

266 H Isotropic = 28.7829 Anisotropy = 6.7185

XX= 28.2891 YX= 0.9762 ZX= 0.6550

XY= 0.8776 YY= 28.0294 ZY= -2.5216

XZ= 3.7127 YZ= -5.0745 ZZ= 30.0302

Eigenvalues: 24.0552 29.0316 33.2619

267 H Isotropic = 24.5361 Anisotropy = 6.4720

XX= 25.6197 YX= -1.1758 ZX= 1.8313

XY= 1.9880 YY= 28.0054 ZY= 0.3495

XZ= 0.1993 YZ= -5.8163 ZZ= 19.9832

Eigenvalues: 18.9620 25.7956 28.8508

268 H Isotropic = 23.8881 Anisotropy = 8.1867

XX= 22.4513 YX= 2.0328 ZX= 1.1797

XY= 1.7596 YY= 28.1465 ZY= 2.3121

XZ= 1.3457 YZ= 1.6651 ZZ= 21.0667

Eigenvalues: 20.2195 22.0990 29.3459

269 H Isotropic = 21.9994 Anisotropy = 12.8162

XX= 24.7942 YX= -4.6511 ZX= 5.0664

XY= -4.0200 YY= 26.9438 ZY= 0.9877

XZ= 2.3642 YZ= 0.3333 ZZ= 14.2600

Eigenvalues: 12.7914 22.6632 30.5435

270 H Isotropic = 24.0522 Anisotropy = 6.1655

XX= 22.7630 YX= 1.1949 ZX= 0.8941

XY= 0.4040 YY= 27.8680 ZY= 0.9793

XZ= 0.0724 YZ= 1.0335 ZZ= 21.5257

Eigenvalues: 21.2780 22.7161 28.1626

271 H Isotropic = 24.3803 Anisotropy = 5.3865

XX= 25.7883 YX= 1.6702 ZX= 0.9339

XY= 1.8133 YY= 25.0307 ZY= 1.9840

XZ= 1.4061 YZ= 1.7308 ZZ= 22.3219

Eigenvalues: 21.3591 23.8105 27.9713

272 H Isotropic = 29.7879 Anisotropy = 4.9285

XX= 30.1956 YX= -0.0338 ZX= 0.9880

XY= -1.7195 YY= 29.7031 ZY= 3.7328

XZ= 2.3193 YZ= 3.0192 ZZ= 29.4649

Eigenvalues: 25.5101 30.7800 33.0735

273 O Isotropic = 293.5104 Anisotropy = 42.8484

XX= 294.8874 YX= -0.6200 ZX= 16.8751

XY= 1.7119 YY= 282.8779 ZY= 20.4326

XZ= 16.9059 YZ= 16.0654 ZZ= 302.7658

Eigenvalues: 268.8873 289.5679 322.0760

274 H Isotropic = 28.5402 Anisotropy = 21.8723

XX= 42.6794 YX= 0.0043 ZX= 3.9733

XY= 0.1737 YY= 22.0681 ZY= 0.5219

XZ= 2.2896 YZ= 0.8397 ZZ= 20.8732

Eigenvalues: 20.1980 22.3009 43.1217

275 H Isotropic = 23.9741 Anisotropy = 27.4706

XX= 16.6578 YX= 3.8628 ZX= 6.8951

XY= 4.3061 YY= 17.1977 ZY= 5.7165

XZ= 8.1403 YZ= 5.9256 ZZ= 38.0668

Eigenvalues: 12.6805 16.9540 42.2878

276 C Isotropic = 144.7320 Anisotropy = 42.3467

XX= 159.3647 YX= -17.3879 ZX= -10.1333

XY= -7.8820 YY= 146.4470 ZY= 4.8049

XZ= -16.2426 YZ= 14.0341 ZZ= 128.3842

Eigenvalues: 122.4495 138.7833 172.9631

277 C Isotropic = 22.2443 Anisotropy = 133.9539

XX= 31.0566 YX= 80.3839 ZX= 10.5540

XY= 73.5211 YY= 34.4863 ZY= 12.4746

XZ= -1.3872 YZ= 17.9673 ZZ= 1.1900

Eigenvalues: -45.3974 0.5835 111.5469

278 O Isotropic = -49.2575 Anisotropy = 540.9649

XX= 77.0656 YX= 229.2449 ZX= 44.8170

XY= 223.0496 YY= 67.8456 ZY= 89.2837

XZ= 33.4851 YZ= 80.9582 ZZ= -292.6836

Eigenvalues: -312.2638 -146.8944 311.3858

279 C Isotropic = 158.2389 Anisotropy = 16.4966

XX= 165.4060 YX= -5.2414 ZX= -5.8771

XY= -5.3483 YY= 158.0972 ZY= -1.6031

XZ= -6.6121 YZ= -4.7110 ZZ= 151.2136

Eigenvalues: 146.6856 158.7944 169.2366

280 C Isotropic = 159.5588 Anisotropy = 26.7251

XX= 168.8090 YX= -7.9180 ZX= -10.1030

XY= -9.4004 YY= 161.4430 ZY= 3.2192

XZ= -9.9491 YZ= -1.2521 ZZ= 148.4245

Eigenvalues: 143.9468 157.3541 177.3755

281 C Isotropic = 172.0842 Anisotropy = 15.8976

XX= 169.5159 YX= 1.4373 ZX= 2.4508

XY= 1.1037 YY= 167.5393 ZY= 4.8386

XZ= 5.2283 YZ= 6.3424 ZZ= 179.1973

Eigenvalues: 165.2719 168.2980 182.6826

282 C Isotropic = 172.1185 Anisotropy = 22.9928

XX= 172.5703 YX= 6.1656 ZX= -5.0063

XY=   11.2808   YY=   174.1608   ZY=   -8.5378

XZ=   -5.5245   YZ=   -8.1263   ZZ=   169.6243

Eigenvalues: 162.9321   165.9763   187.4470

283 H   Isotropic =   30.2550   Anisotropy =   10.9407

XX=   33.4204   YX=   -4.1873   ZX=   -3.3083

XY=   -3.7499   YY=   31.2077   ZY=   1.7591

XZ=   -3.0648   YZ=   1.6254   ZZ=   26.1369

Eigenvalues: 24.9314   28.2847   37.5488

284 H   Isotropic =   29.4000   Anisotropy =   5.8200

XX=   30.9343   YX=   0.8174   ZX=   0.5079

XY=   0.7615   YY=   28.2134   ZY=   4.8554

XZ=   1.6010   YZ=   2.9251   ZZ=   29.0522

Eigenvalues: 24.7177   30.2023   33.2800

285 H   Isotropic =   29.8427   Anisotropy =   4.4121

XX=   29.9832   YX=   0.1887   ZX=   -2.7030

XY=   0.6398   YY=   31.5689   ZY=   -1.7161

XZ=   -3.9041   YZ=   0.6419   ZZ=   27.9758

Eigenvalues: 25.5212   31.2228   32.7841

286 H   Isotropic =   30.1807   Anisotropy =   4.0119

XX=   29.6456   YX=   -0.0148   ZX=   -0.6701

XY=   0.0142   YY=   28.4615   ZY=   -1.4482

XZ=   -0.0778   YZ=   -1.1251   ZZ=   32.4350

Eigenvalues: 28.0739   29.6129   32.8553

287 H   Isotropic =   30.4603   Anisotropy =   9.4092

XX=   30.4084   YX=   0.4685   ZX=   -1.9633

XY=   0.8498   YY=   32.6656   ZY=   6.1835

XZ=   -2.1262   YZ=   5.3873   ZZ=   28.3069

Eigenvalues: 23.6732   30.9746   36.7331

288 H   Isotropic =   31.1227   Anisotropy =   9.1785

XX=   30.9416   YX=   -2.1103   ZX=   -0.1939

XY=   -2.1374   YY=   36.0533   ZY=   2.1302

XZ=   -0.0309   YZ=   2.3253   ZZ=   26.3732

Eigenvalues: 25.8597 30.2668 37.2417

289 H Isotropic = 30.4092 Anisotropy = 7.8356

XX= 30.9907 YX= 4.1713 ZX= -1.0498

XY= 3.0080 YY= 32.0393 ZY= 3.6997

XZ= -1.5966 YZ= 3.1519 ZZ= 28.1975

Eigenvalues: 24.6588 30.9359 35.6329

290 H Isotropic = 30.5850 Anisotropy = 4.0509

XX= 29.3991 YX= -1.9538 ZX= -1.5162

XY= -1.8630 YY= 31.3078 ZY= -2.2709

XZ= -1.3740 YZ= -1.8087 ZZ= 31.0480

Eigenvalues: 26.8910 31.5783 33.2856

291 H Isotropic = 30.7010 Anisotropy = 7.5988

XX= 33.2737 YX= 3.2972 ZX= -1.7233

XY= 4.0222 YY= 29.9502 ZY= 3.6718

XZ= -0.9903 YZ= 3.6194 ZZ= 28.8793

Eigenvalues: 24.3565 31.9797 35.7669

292 H Isotropic = 31.1111 Anisotropy = 8.3708

XX= 28.6620 YX= 2.6639 ZX= -3.6147

XY= 1.5789 YY= 31.0547 ZY= -2.1210

XZ= -3.0343 YZ= -1.9953 ZZ= 33.6166

Eigenvalues: 26.7173 29.9243 36.6916

293 H Isotropic = 29.6006 Anisotropy = 11.5016

XX= 27.0864 YX= 0.9803 ZX= 0.0321

XY= 1.8404 YY= 24.6751 ZY= -1.1694

XZ= 0.4659 YZ= -2.2173 ZZ= 37.0403

Eigenvalues: 23.8204 27.7131 37.2684

294 N Isotropic = 144.2837 Anisotropy = 102.3791

XX= 167.7654 YX= 1.1034 ZX= 25.9293

XY= -16.5975 YY= 204.8944 ZY= -10.7619

XZ= 78.7536 YZ= -19.0575 ZZ= 60.1913

Eigenvalues: 38.2101 182.1045 212.5365

295 C Isotropic = 143.2174 Anisotropy = 32.0009

XX=   156.5449   YX=   10.6498   ZX=   -11.0457

XY=   13.8325   YY=   142.6087   ZY=   8.9219

XZ=   -10.4905   YZ=   8.1115   ZZ=   130.4985

Eigenvalues: 119.6329   145.4679   164.5513

296 C   Isotropic =   24.7542   Anisotropy =   124.3054

XX=   28.2643   YX=   -62.2016   ZX=   29.8928

XY=   -66.2389   YY=   18.1025   ZY=   -26.5560

XZ=   18.7520   YZ=   -38.2743   ZZ=   27.8958

Eigenvalues: -42.1596   8.7978   107.6245

297 O   Isotropic =   -36.0284   Anisotropy =   535.0552

XX=   47.8395   YX= -161.8031   ZX=   155.2380

XY= -171.6000   YY=   24.8266   ZY= -154.2428

XZ=   185.6037   YZ= -191.1225   ZZ= -180.7513

Eigenvalues: -298.4328 -130.3275   320.6751

298 H   Isotropic =   27.6184   Anisotropy =   7.8295

XX=   27.3213   YX=   -2.5048   ZX=   0.6901

XY=   -1.7826   YY=   31.3687   ZY=   -2.2692

XZ=   0.4867   YZ=   -1.9551   ZZ=   24.1653

Eigenvalues: 23.5917   26.4255   32.8381

299 H   Isotropic =   25.5167   Anisotropy =   3.1083

XX=   26.3032   YX=   -2.4805   ZX=   -2.5257

XY=   -0.1526   YY=   24.7615   ZY=   -2.0757

XZ=   -0.6407   YZ=   -0.2127   ZZ=   25.4852

Eigenvalues: 22.7707   26.1904   27.5888

300 H   Isotropic =   27.3103   Anisotropy =   5.4627

XX=   27.2704   YX=   2.2523   ZX=   -0.1306

XY=   0.8698   YY=   28.1342   ZY=   4.3138

XZ=   -0.9924   YZ=   2.2804   ZZ=   26.5263

Eigenvalues: 23.4101   27.5687   30.9521

301 N   Isotropic =   137.2249   Anisotropy =   105.1512

XX=   130.5577   YX=   10.5907   ZX=   58.1594

XY=   18.0430   YY=   186.9563   ZY=   -3.1605

XZ= 81.0607 YZ= 36.7207 ZZ= 94.1607

Eigenvalues: 40.2665 164.0825 207.3257

302 C Isotropic = 126.9747 Anisotropy = 19.2198

XX= 123.2785 YX= -1.5120 ZX= 5.0390

XY= -1.4961 YY= 123.9259 ZY= -5.8866

XZ= 10.6002 YZ= -4.8838 ZZ= 133.7197

Eigenvalues: 118.6967 122.4395 139.7879

303 C Isotropic = 25.2022 Anisotropy = 122.8842

XX= -4.0973 YX= 54.4577 ZX= -20.9735

XY= 48.7846 YY= 72.9638 ZY= 42.7034

XZ= -4.1674 YZ= 32.5161 ZZ= 6.7400

Eigenvalues: -46.4835 14.9651 107.1250

304 O Isotropic = -62.6286 Anisotropy = 564.9165

XX= -190.2615 YX= 167.5451 ZX= 130.0666

XY= 177.2196 YY= 208.8315 ZY= 87.5163

XZ= 145.9941 YZ= 116.8270 ZZ= -206.4558

Eigenvalues: -341.2874 -160.5807 313.9824

305 C Isotropic = 149.7660 Anisotropy = 16.0471

XX= 151.9628 YX= -1.0108 ZX= 3.2939

XY= -4.4642 YY= 141.2875 ZY= 3.3268

XZ= 4.8072 YZ= 12.7709 ZZ= 156.0478

Eigenvalues: 136.6109 152.2230 160.4641

306 C Isotropic = 162.1712 Anisotropy = 22.0563

XX= 157.9101 YX= -8.8777 ZX= 9.7621

XY= -8.3143 YY= 158.7923 ZY= -1.9704

XZ= 7.4663 YZ= -3.5943 ZZ= 169.8111

Eigenvalues: 148.6833 160.9548 176.8754

307 C Isotropic = 175.1487 Anisotropy = 23.4762

XX= 177.9517 YX= 11.1031 ZX= -1.7627

XY= 7.1319 YY= 179.3936 ZY= -9.5971

XZ= -2.4362 YZ= -8.4318 ZZ= 168.1010

Eigenvalues: 162.5422 172.1045 190.7996

308 C Isotropic = 178.6231 Anisotropy = 26.1653

XX= 174.8592 YX= -6.6248 ZX= -0.3470

XY= -4.7517 YY= 170.5589 ZY= 13.6162

XZ= 1.6428 YZ= 9.8997 ZZ= 190.4513

Eigenvalues: 162.6646 177.1382 196.0666

309 H Isotropic = 30.5614 Anisotropy = 11.1190

XX= 27.9659 YX= -1.6773 ZX= 4.0505

XY= -1.1629 YY= 27.4660 ZY= 0.8314

XZ= 4.2283 YZ= -0.2895 ZZ= 36.2523

Eigenvalues: 25.3169 28.3933 37.9741

310 H Isotropic = 25.9693 Anisotropy = 9.6951

XX= 28.0600 YX= 4.3802 ZX= 0.2104

XY= 5.8099 YY= 25.5337 ZY= 3.6388

XZ= -0.9525 YZ= 2.8052 ZZ= 24.3144

Eigenvalues: 19.7717 25.7036 32.4327

311 H Isotropic = 27.8579 Anisotropy = 4.7620

XX= 29.2281 YX= -1.7861 ZX= -1.4292

XY= -2.0671 YY= 27.7615 ZY= 2.5693

XZ= 1.5405 YZ= 2.1967 ZZ= 26.5841

Eigenvalues: 24.3761 28.1651 31.0326

312 H Isotropic = 30.7397 Anisotropy = 3.4617

XX= 32.9532 YX= 0.1153 ZX= 0.7747

XY= -0.1864 YY= 29.2220 ZY= 3.3004

XZ= -0.1111 YZ= 2.2412 ZZ= 30.0438

Eigenvalues: 26.8221 32.3494 33.0475

313 H Isotropic = 30.3096 Anisotropy = 6.6276

XX= 26.4110 YX= 1.2604 ZX= 3.1053

XY= 2.1482 YY= 33.1110 ZY= 1.1502

XZ= 2.4374 YZ= 1.2009 ZZ= 31.4068

Eigenvalues: 25.0262 31.1746 34.7280

314 H Isotropic = 30.7737 Anisotropy = 6.9550

XX= 27.7942 YX= 0.8582 ZX= 3.8664

XY= 0.2643 YY= 33.9544 ZY= -2.1635

XZ= 4.1670 YZ= -2.3512 ZZ= 30.5724

Eigenvalues: 24.5903 32.3203 35.4103

315 H Isotropic = 30.5027 Anisotropy = 10.6327

XX= 26.4555 YX= 3.0881 ZX= 1.2658

XY= 3.4494 YY= 34.1087 ZY= 3.0596

XZ= 1.1781 YZ= 4.3304 ZZ= 30.9439

Eigenvalues: 25.2453 28.6717 37.5912

316 H Isotropic = 30.1850 Anisotropy = 10.0604

XX= 35.0521 YX= -3.5999 ZX= 0.0472

XY= -4.5622 YY= 26.7427 ZY= 2.6629

XZ= -0.3637 YZ= 2.6016 ZZ= 28.7601

Eigenvalues: 23.8893 29.7737 36.8919

317 H Isotropic = 30.4978 Anisotropy = 11.7199

XX= 26.2079 YX= 3.9478 ZX= 2.4726

XY= 3.8922 YY= 34.7567 ZY= 3.9365

XZ= 1.4552 YZ= 3.0503 ZZ= 30.5288

Eigenvalues: 24.6130 28.5692 38.3110

318 H Isotropic = 31.1154 Anisotropy = 8.8457

XX= 32.9947 YX= 0.2563 ZX= -2.7160

XY= -0.8981 YY= 27.3792 ZY= 3.3352

XZ= -3.8837 YZ= 3.2870 ZZ= 32.9724

Eigenvalues: 25.6483 30.6854 37.0125

319 H Isotropic = 30.9006 Anisotropy = 6.1390

XX= 33.8549 YX= 0.7221 ZX= -2.2821

XY= 2.0446 YY= 29.3917 ZY= 1.5161

XZ= -2.4467 YZ= 1.0953 ZZ= 29.4550

Eigenvalues: 27.0671 30.6413 34.9932

320 N Isotropic = 139.3283 Anisotropy = 102.0173

XX= 177.5760 YX= -26.1763 ZX= 68.4327

XY= -26.9414 YY= 172.6539 ZY= 10.8020

XZ= 31.2712 YZ= 28.5176 ZZ= 67.7551

Eigenvalues: 42.4256 168.2195 207.3399

321 C Isotropic = 137.6606 Anisotropy = 20.6279

XX= 121.4084 YX= 4.6763 ZX= 1.4373

XY= 7.5824 YY= 143.7948 ZY= 3.6260

XZ= 1.8511 YZ= 5.2014 ZZ= 147.7786

Eigenvalues: 119.8309 141.7384 151.4125

322 C Isotropic = 26.1469 Anisotropy = 116.2195

XX= 88.5678 YX= -33.9003 ZX= 15.9412

XY= -29.6850 YY= -23.6126 ZY= -33.4187

XZ= 19.1609 YZ= -21.9218 ZZ= 13.4855

Eigenvalues: -41.7215 16.5357 103.6266

323 O Isotropic = -36.0768 Anisotropy = 521.6459

XX= 226.4326 YX= -140.3161 ZX= 163.4571

XY= -132.1887 YY= -120.4938 ZY= -1.7205

XZ= 143.7411 YZ= 30.4325 ZZ= -214.1692

Eigenvalues: -284.3622 -135.5553 311.6871

324 C Isotropic = 174.6903 Anisotropy = 27.6866

XX= 188.7643 YX= -11.4259 ZX= -2.1744

XY= -7.8071 YY= 167.9454 ZY= 2.1452

XZ= -5.3118 YZ= 0.6967 ZZ= 167.3611

Eigenvalues: 164.1827 166.7401 193.1480

325 H Isotropic = 30.7314 Anisotropy = 10.9815

XX= 28.5577 YX= -2.8384 ZX= -2.2188

XY= -2.6114 YY= 34.6666 ZY= 4.3098

XZ= -1.2283 YZ= 4.2579 ZZ= 28.9699

Eigenvalues: 26.6167 27.5251 38.0524

326 H Isotropic = 26.8967 Anisotropy = 5.3553

XX= 29.5811 YX= -1.2273 ZX= 1.2214

XY= -0.5669 YY= 25.0494 ZY= -0.6934

XZ= 1.7852 YZ= -2.0470 ZZ= 26.0596

Eigenvalues: 24.0908 26.1325 30.4669

327 H Isotropic = 27.9487 Anisotropy = 6.0842

XX=   26.7327   YX=   1.8548   ZX=   -1.1731

XY=   2.2133   YY=   29.6347   ZY=   -3.5128

XZ=   -1.3413   YZ=   -0.6942   ZZ=   27.4788

Eigenvalues: 25.6480   26.1933   32.0049

328 H   Isotropic =   30.1769   Anisotropy =   8.4714

XX=   29.6350   YX=   -3.7291   ZX=   2.6962

XY=   -3.1945   YY=   30.6443   ZY=   -2.6183

XZ=   2.0694   YZ=   -2.4890   ZZ=   30.2514

Eigenvalues: 26.6320   28.0742   35.8245

329 H   Isotropic =   29.9544   Anisotropy =   8.7106

XX=   31.0118   YX=   3.6321   ZX=   -2.9771

XY=   2.0143   YY=   33.0599   ZY=   -1.4450

XZ=   -1.7839   YZ=   -1.7191   ZZ=   25.7914

Eigenvalues: 24.8383   29.2633   35.7614

330 N   Isotropic =   140.7865   Anisotropy =   88.4825

XX= 178.3710   YX=   23.4334   ZX=   33.0567

XY=   3.6822   YY= 190.8896   ZY=   11.8380

XZ=   23.1106   YZ= -35.9885   ZZ=   53.0988

Eigenvalues: 45.6065 176.9781 199.7748

331 C   Isotropic =   136.3618   Anisotropy =   13.1306

XX= 144.0606   YX=   -5.1993   ZX=   2.9085

XY=   -2.7973   YY= 125.6274   ZY=   9.7783

XZ=   -0.8369   YZ=   7.1012   ZZ= 139.3975

Eigenvalues: 120.9091 143.0608 145.1156

332 C   Isotropic =   25.7109   Anisotropy = 120.1631

XX= -38.8418   YX=   19.3613   ZX=   7.0475

XY=   18.0753   YY= 103.3419   ZY=   -3.9340

XZ=   -6.9375   YZ=   -0.6367   ZZ=   12.6326

Eigenvalues: -41.2670   12.5801 105.8196

333 O   Isotropic =   -38.6120   Anisotropy = 524.3954

XX= -132.6345   YX=   60.6824   ZX=   -4.8813

XY=   57.8521   YY= 302.9818   ZY=   19.4248

XZ= -30.7937 YZ= -28.9018 ZZ= -286.1833

Eigenvalues: -288.2355 -138.5853 310.9849

334 C Isotropic = 159.8189 Anisotropy = 17.9113

XX= 157.7015 YX= 6.0769 ZX= 0.6879

XY= 8.4547 YY= 167.7964 ZY= -0.0879

XZ= 2.3753 YZ= 2.3362 ZZ= 153.9589

Eigenvalues: 153.0143 154.6827 171.7597

335 C Isotropic = 61.0425 Anisotropy = 116.9629

XX= 116.5811 YX= -51.1500 ZX= -3.1616

XY= -50.3021 YY= 23.2328 ZY= -27.6565

XZ= -3.5370 YZ= -7.9588 ZZ= 43.3135

Eigenvalues: -5.4329 49.5425 139.0177

336 C Isotropic = 82.1177 Anisotropy = 110.2749

XX= 136.4472 YX= -44.5391 ZX= 7.0193

XY= -51.5292 YY= 35.0235 ZY= 8.8306

XZ= 4.1413 YZ= 8.4427 ZZ= 74.8825

Eigenvalues: 14.2075 76.5114 155.6343

337 N Isotropic = 11.1906 Anisotropy = 310.5849

XX= 158.6133 YX= -134.3648 ZX= -4.1989

XY= -128.7310 YY= -72.0515 ZY= -19.3634

XZ= -15.2936 YZ= -34.7087 ZZ= -52.9901

Eigenvalues: -140.9936 -43.6818 218.2471

338 C Isotropic = 70.0901 Anisotropy = 105.2796

XX= 117.0626 YX= -52.1346 ZX= 8.3737

XY= -47.6462 YY= 32.9174 ZY= 21.2423

XZ= 0.5582 YZ= 4.5264 ZZ= 60.2902

Eigenvalues: 6.3170 63.6767 140.2765

339 N Isotropic = 105.0046 Anisotropy = 131.3113

XX= 170.9040 YX= -45.0031 ZX= -15.6036

XY= -38.6795 YY= 111.4759 ZY= -22.5510

XZ= -16.0574 YZ= -28.4991 ZZ= 32.6338

Eigenvalues: 19.8054 102.6629 192.5455

340 H Isotropic = 23.6799 Anisotropy = 9.3618

XX= 19.2034 YX= -1.3901 ZX= 3.2465

XY= 1.8558 YY= 22.3759 ZY= 0.1851

XZ= 1.1764 YZ= 0.0792 ZZ= 29.4605

Eigenvalues: 18.7357 22.3829 29.9211

341 H Isotropic = 22.9440 Anisotropy = 9.8517

XX= 19.8671 YX= 2.1038 ZX= -7.0610

XY= 3.7713 YY= 22.4886 ZY= -1.0618

XZ= -3.1948 YZ= 1.4327 ZZ= 26.4762

Eigenvalues: 15.9611 23.3591 29.5118

342 H Isotropic = 27.7370 Anisotropy = 5.0313

XX= 27.4826 YX= 0.5745 ZX= 3.8049

XY= 0.7079 YY= 26.1988 ZY= 0.8498

XZ= 0.3973 YZ= 0.9459 ZZ= 29.5297

Eigenvalues: 25.9325 26.1874 31.0912

343 H Isotropic = 28.6139 Anisotropy = 7.0629

XX= 29.7890 YX= 0.9695 ZX= 4.7556

XY= -0.3168 YY= 27.5878 ZY= -1.5736

XZ= 3.4786 YZ= -0.3623 ZZ= 28.4649

Eigenvalues: 24.6491 27.8700 33.3225

344 H Isotropic = 24.9903 Anisotropy = 7.6620

XX= 23.2988 YX= 3.3047 ZX= 1.7467

XY= 5.0648 YY= 23.9226 ZY= 3.0461

XZ= 0.8602 YZ= 0.8473 ZZ= 27.7495

Eigenvalues: 19.3967 25.4759 30.0983

345 H Isotropic = 27.5705 Anisotropy = 10.5041

XX= 23.9583 YX= 3.8326 ZX= -1.5157

XY= 4.0209 YY= 33.0948 ZY= 0.0607

XZ= -1.8199 YZ= 0.2269 ZZ= 25.6584

Eigenvalues: 21.8212 26.3171 34.5733

346 H Isotropic = 28.3605 Anisotropy = 6.1295

XX= 28.8451 YX= 1.8548 ZX= -4.0548

XY= 2.0870 YY= 27.3723 ZY= 2.7319

XZ= -3.1052 YZ= 1.8098 ZZ= 28.8641

Eigenvalues: 23.1445 29.4901 32.4468

347 N Isotropic = 136.8763 Anisotropy = 96.8778

XX= 180.3188 YX= 3.5108 ZX= 29.9497

XY= -5.2582 YY= 175.4723 ZY= -15.4149

XZ= 75.4717 YZ= -19.9668 ZZ= 54.8378

Eigenvalues: 33.7507 175.4168 201.4615

348 C Isotropic = 133.4168 Anisotropy = 22.9325

XX= 130.3523 YX= 4.0457 ZX= -11.0180

XY= 6.2366 YY= 141.7825 ZY= -5.4041

XZ= -7.1152 YZ= -8.3126 ZZ= 128.1157

Eigenvalues: 119.9322 131.6131 148.7051

349 C Isotropic = 26.0495 Anisotropy = 116.8460

XX= 91.0544 YX= -7.5473 ZX= 29.6580

XY= -12.5737 YY= -45.6130 ZY= 14.9125

XZ= 30.2162 YZ= 0.5513 ZZ= 32.7072

Eigenvalues: -47.6694 21.8711 103.9469

350 O Isotropic = -58.2675 Anisotropy = 568.2626

XX= 227.1463 YX= 11.0839 ZX= 201.3859

XY= -8.0902 YY= -178.5582 ZY= -57.1456

XZ= 246.6489 YZ= -66.7677 ZZ= -223.3907

Eigenvalues: -337.0312 -158.3455 320.5742

351 C Isotropic = 153.8568 Anisotropy = 27.1411

XX= 168.6420 YX= -12.1057 ZX= -5.2705

XY= -5.4521 YY= 143.3367 ZY= 1.8378

XZ= -0.5807 YZ= 3.1555 ZZ= 149.5917

Eigenvalues: 140.3323 149.2872 171.9508

352 C Isotropic = 164.2708 Anisotropy = 8.5263

XX= 166.0707 YX= -4.5336 ZX= 2.5386

XY= -6.0568 YY= 162.6460 ZY= -1.6882

XZ= -2.2907 YZ= 0.5694 ZZ= 164.0958

Eigenvalues: 158.7660 164.0914 169.9551

353 C Isotropic = 168.9778 Anisotropy = 29.9574

XX= 157.3104 YX= -0.6318 ZX= -1.7169

XY= 5.2204 YY= 174.6725 ZY= 16.7989

XZ= -1.0924 YZ= 11.4512 ZZ= 174.9506

Eigenvalues: 155.8738 162.1102 188.9494

354 C Isotropic = 163.8229 Anisotropy = 32.1907

XX= 164.3196 YX= -1.7017 ZX= 14.4861

XY= -4.1738 YY= 152.8695 ZY= -1.0933

XZ= 15.8147 YZ= 2.6936 ZZ= 174.2796

Eigenvalues: 150.2336 155.9517 185.2833

355 H Isotropic = 30.7419 Anisotropy = 8.0401

XX= 27.8032 YX= -1.7416 ZX= 1.9957

XY= -0.3469 YY= 32.8283 ZY= -3.1404

XZ= 1.5181 YZ= -3.6273 ZZ= 31.5942

Eigenvalues: 27.0934 29.0304 36.1020

356 H Isotropic = 24.8262 Anisotropy = 4.5618

XX= 26.2827 YX= -1.4030 ZX= 0.7447

XY= -1.0072 YY= 20.8642 ZY= 0.7543

XZ= -1.4260 YZ= 2.0831 ZZ= 27.3317

Eigenvalues: 20.3573 26.2539 27.8674

357 H Isotropic = 28.7890 Anisotropy = 4.1163

XX= 29.1014 YX= 1.0862 ZX= -0.1461

XY= -1.9571 YY= 30.1239 ZY= 3.2177

XZ= 0.3427 YZ= 1.6512 ZZ= 27.1415

Eigenvalues: 25.7511 29.0826 31.5331

358 H Isotropic = 30.3979 Anisotropy = 6.1259

XX= 30.0073 YX= -2.9628 ZX= 0.4650

XY= -3.2567 YY= 29.1909 ZY= 3.2423

XZ= 0.3007 YZ= 2.8320 ZZ= 31.9954

Eigenvalues: 25.4210 31.2908 34.4818

359 H Isotropic = 30.2187 Anisotropy = 7.3554

XX= 29.9568 YX= 0.4633 ZX= 1.8601

XY= 1.3610 YY= 33.3986 ZY= -3.5286

XZ= 1.6242 YZ= -3.8111 ZZ= 27.3008

Eigenvalues: 24.8261 30.7076 35.1223

360 H Isotropic = 30.9741 Anisotropy = 8.3552

XX= 33.3413 YX= -1.2399 ZX= -3.3412

XY= -1.8196 YY= 28.6523 ZY= 1.5430

XZ= -3.5916 YZ= 2.4526 ZZ= 30.9286

Eigenvalues: 27.4299 28.9481 36.5442

361 H Isotropic = 30.5854 Anisotropy = 8.8128

XX= 36.3730 YX= 0.8470 ZX= -0.7842

XY= 0.8946 YY= 27.3461 ZY= -0.0206

XZ= 0.4370 YZ= -0.3647 ZZ= 28.0370

Eigenvalues: 27.2248 28.0707 36.4606

362 H Isotropic = 30.6195 Anisotropy = 5.3771

XX= 30.0035 YX= 0.7059 ZX= 0.7879

XY= 0.4909 YY= 32.4443 ZY= -2.2776

XZ= -0.2904 YZ= -3.4512 ZZ= 29.4107

Eigenvalues: 27.5732 30.0811 34.2042

363 H Isotropic = 32.1142 Anisotropy = 10.6991

XX= 29.6321 YX= 1.5017 ZX= -0.0566

XY= 4.3352 YY= 38.3590 ZY= -0.5076

XZ= -0.0632 YZ= 0.8454 ZZ= 28.3516

Eigenvalues: 28.3229 28.7729 39.2470

364 H Isotropic = 30.8588 Anisotropy = 9.1660

XX= 28.9153 YX= 1.6439 ZX= 3.0428

XY= 1.1807 YY= 29.0364 ZY= 2.5537

XZ= 2.0274 YZ= 3.4530 ZZ= 34.6248

Eigenvalues: 27.5268 28.0802 36.9695

365 H Isotropic = 29.3411 Anisotropy = 7.9540

XX= 27.6095 YX= -3.8950 ZX= 1.8709

XY= -2.0321 YY= 27.1493 ZY= 0.2034

XZ= 3.9695 YZ= 0.2146 ZZ= 33.2645

Eigenvalues: 23.8857 29.4939 34.6438

366 N Isotropic = 139.1211 Anisotropy = 94.1185

XX= 148.3398 YX= -11.7721 ZX= 52.2400

XY= -5.6655 YY= 195.4012 ZY= 5.8072

XZ= 49.5173 YZ= 50.9194 ZZ= 73.6222

Eigenvalues: 42.2682 173.2283 201.8668

367 C Isotropic = 135.9242 Anisotropy = 20.1460

XX= 140.1427 YX= 5.1528 ZX= 4.8349

XY= 6.0356 YY= 123.8688 ZY= -10.4993

XZ= 6.8270 YZ= -9.5494 ZZ= 143.7611

Eigenvalues: 117.2603 141.1574 149.3548

368 C Isotropic = 28.3491 Anisotropy = 113.9611

XX= -24.0625 YX= -17.6344 ZX= -43.8698

XY= -18.0884 YY= 101.7662 ZY= -3.2652

XZ= -28.1665 YZ= -1.7529 ZZ= 7.3436

Eigenvalues: -49.4214 30.1455 104.3232

369 O Isotropic = -66.0596 Anisotropy = 574.0307

XX= -217.7002 YX= -99.6972 ZX= 66.1919

XY= -88.8390 YY= 298.6395 ZY= 21.6865

XZ= 93.6547 YZ= 62.8057 ZZ= -279.1180

Eigenvalues: -346.3916 -168.4147 316.6276

370 C Isotropic = 174.4475 Anisotropy = 24.4562

XX= 164.9698 YX= -4.1211 ZX= -2.6788

XY= -1.0877 YY= 185.0371 ZY= 8.6733

XZ= -3.5219 YZ= 9.9784 ZZ= 173.3357

Eigenvalues: 163.9293 168.6616 190.7517

371 H Isotropic = 30.7137 Anisotropy = 8.1059

XX= 27.9933 YX= 2.9496 ZX= 2.4239

XY= 2.9647 YY= 31.1042 ZY= 2.0326

XZ= 2.9721 YZ= 1.8600 ZZ= 33.0437

Eigenvalues: 25.9139 30.1096 36.1177

372 H Isotropic = 24.9597 Anisotropy = 4.6013

XX= 21.5862 YX= 0.1084 ZX= -3.0999

XY= 0.2047 YY= 27.6196 ZY= 0.2584

XZ= -4.0870 YZ= -0.8347 ZZ= 25.6734

Eigenvalues: 19.4959 27.3561 28.0273

373 H Isotropic = 27.7634 Anisotropy = 7.0847

XX= 29.8187 YX= 2.3345 ZX= -3.8790

XY= 1.9330 YY= 28.2720 ZY= -2.5204

XZ= -0.8700 YZ= -0.8851 ZZ= 25.1994

Eigenvalues: 24.0276 26.7761 32.4865

374 H Isotropic = 30.4509 Anisotropy = 12.3405

XX= 25.9233 YX= 2.8745 ZX= -0.9713

XY= 3.3116 YY= 37.3307 ZY= -2.5834

XZ= -0.8934 YZ= -1.9746 ZZ= 28.0985

Eigenvalues: 25.0924 27.5823 38.6779

375 H Isotropic = 29.8220 Anisotropy = 5.4434

XX= 31.8990 YX= 2.2847 ZX= -2.5595

XY= 0.5761 YY= 30.2386 ZY= 0.8432

XZ= -2.7691 YZ= 0.6425 ZZ= 27.3284

Eigenvalues: 25.7333 30.2817 33.4509

376 N Isotropic = 143.9556 Anisotropy = 93.6734

XX= 200.6743 YX= -1.1543 ZX= 52.1278

XY= -1.9410 YY= 173.5391 ZY= 14.5543

XZ= 5.9012 YZ= 16.5635 ZZ= 57.6533

Eigenvalues: 50.0291 175.4332 206.4045

377 C Isotropic = 137.0646 Anisotropy = 21.7806

XX= 116.8750 YX= -6.0467 ZX= 1.6041

XY= -2.0386 YY= 147.7251 ZY= 2.7732

XZ= 2.4178 YZ= 5.8245 ZZ= 146.5937

Eigenvalues: 116.1380 143.4708 151.5850

378 C Isotropic = 25.8378 Anisotropy = 119.9291

XX= 90.1666 YX= 35.3944 ZX= 23.4026

XY=   39.2285   YY=   -36.3047   ZY=   -20.5695

XZ=   26.3787   YZ=   -3.4155   ZZ=   23.6515

Eigenvalues: -51.0643   22.7872   105.7905

379 O   Isotropic =   -43.0398   Anisotropy =   559.5202

XX=   268.8258   YX=   84.6971   ZX=   169.6587

XY=   94.6285   YY= -144.4283   ZY=   65.9201

XZ=   118.0095   YZ=   92.1668   ZZ= -253.5170

Eigenvalues: -310.4258 -148.6674   329.9736

380 C   Isotropic =   172.9729   Anisotropy =   21.4546

XX=   186.4842   YX=   2.4797   ZX=   -0.0738

XY=   4.8792   YY=   165.8241   ZY=   0.8371

XZ=   -3.6036   YZ=   -0.6410   ZZ=   166.6103

Eigenvalues: 165.0681   166.5745   187.2759

381 H   Isotropic =   30.7702   Anisotropy =   6.5175

XX=   30.3149   YX=   -2.7112   ZX=   -1.2591

XY=   -3.1899   YY=   31.8382   ZY=   1.2262

XZ=   -0.9753   YZ=   2.6444   ZZ=   30.1577

Eigenvalues: 27.9640   29.2315   35.1152

382 H   Isotropic =   23.8727   Anisotropy =   7.8215

XX=   23.1627   YX=   2.2190   ZX=   -1.4832

XY=   2.2657   YY=   19.8912   ZY=   -1.6459

XZ=   0.3469   YZ=   -1.8782   ZZ=   28.5643

Eigenvalues: 18.5734   23.9577   29.0871

383 H   Isotropic =   27.8390   Anisotropy =   8.1558

XX=   24.9327   YX=   1.5682   ZX=   1.7086

XY=   1.0687   YY=   30.3694   ZY=   -5.6302

XZ=   0.4939   YZ=   -2.0022   ZZ=   28.2148

Eigenvalues: 23.4358   26.8049   33.2762

384 H   Isotropic =   30.3736   Anisotropy =   10.7494

XX=   32.0907   YX=   2.5218   ZX=   5.5778

XY=   0.4651   YY=   27.6687   ZY=   -0.3957

XZ=   5.4055   YZ=   2.3224   ZZ=   31.3614

Eigenvalues: 26.1420 27.4390 37.5399

385 H Isotropic = 30.9283 Anisotropy = 6.2087

XX= 29.6982 YX= 3.5343 ZX= 0.6050

XY= 0.9688 YY= 32.4206 ZY= -3.9741

XZ= 1.2979 YZ= -2.1930 ZZ= 30.6661

Eigenvalues: 26.7284 30.9890 35.0674

386 N Isotropic = 151.9192 Anisotropy = 103.4974

XX= 173.3697 YX= 13.5364 ZX= 25.8631

XY= -7.9067 YY= 215.8703 ZY= -5.7179

XZ= 33.1735 YZ= -49.3179 ZZ= 66.5176

Eigenvalues: 54.2729 180.5673 220.9175

387 C Isotropic = 144.9286 Anisotropy = 39.5807

XX= 133.6062 YX= -2.8103 ZX= 1.3413

XY= -11.7039 YY= 166.0362 ZY= 22.0708

XZ= 6.8708 YZ= 3.0654 ZZ= 135.1434

Eigenvalues: 125.4097 138.0604 171.3158

388 C Isotropic = 165.6367 Anisotropy = 19.0507

XX= 152.5994 YX= -7.4289 ZX= -7.3322

XY= -5.1779 YY= 176.7925 ZY= 5.7602

XZ= -5.7637 YZ= -8.7825 ZZ= 167.5183

Eigenvalues: 148.6611 169.9118 178.3372

389 C Isotropic = 77.1758 Anisotropy = 124.7207

XX= 30.6910 YX= 28.7538 ZX= 4.1301

XY= 27.2878 YY= 82.7608 ZY= -61.1246

XZ= 1.5340 YZ= -49.9448 ZZ= 118.0756

Eigenvalues: 10.6100 60.5945 160.3230

390 C Isotropic = 81.4664 Anisotropy = 90.5726

XX= 26.4670 YX= 27.9952 ZX= 2.3724

XY= 43.9483 YY= 98.9618 ZY= -27.4210

XZ= -0.1405 YZ= -27.0980 ZZ= 118.9704

Eigenvalues: 10.3780 92.1731 141.8481

391 C Isotropic = 69.9867 Anisotropy = 161.0657

XX=   21.9715   YX=   17.0422   ZX=   -10.9143

XY=   19.6733   YY=   69.5915   ZY=   -83.5332

XZ=   -6.5613   YZ=   -71.5673   ZZ=   118.3970

Eigenvalues: 6.0706   26.5256   177.3638

392 C   Isotropic =   61.9141   Anisotropy =   136.7706

XX=   9.2714   YX=   -7.4124   ZX=   -32.9581

XY=   -13.5753   YY=   82.3561   ZY=   -57.2488

XZ=   -25.2160   YZ=   -68.9032   ZZ=   94.1147

Eigenvalues: -12.1781   44.8258   153.0945

393 C   Isotropic =   77.8853   Anisotropy =   163.1068

XX=   59.4710   YX=   -5.7784   ZX=   -24.5651

XY=   -6.4772   YY=   63.3384   ZY=   -95.8258

XZ=   -19.9512   YZ=   -94.3675   ZZ=   110.8466

Eigenvalues: -15.5603   62.5931   186.6232

394 N   Isotropic =   142.0720   Anisotropy =   68.7165

XX=   145.6043   YX=   -13.1455   ZX=   -19.9008

XY=   -8.6923   YY=   138.6484   ZY=   -51.2971

XZ=   -18.5140   YZ=   -41.7223   ZZ=   141.9633

Eigenvalues: 86.1602   152.1728   187.8830

395 C   Isotropic =   87.3487   Anisotropy =   143.7459

XX=   74.2794   YX=   10.0180   ZX=   -9.5171

XY=   13.9678   YY=   72.7328   ZY=   -85.4264

XZ=   -4.1388   YZ=   -84.9964   ZZ=   115.0340

Eigenvalues: 5.6806   73.1863   183.1793

396 C   Isotropic =   74.9716   Anisotropy =   164.0379

XX=   0.3236   YX=   -14.5618   ZX=   -39.3099

XY=   -19.8637   YY=   102.5593   ZY=   -67.0230

XZ=   -42.8186   YZ=   -71.1857   ZZ=   122.0318

Eigenvalues: -25.1228   65.7074   184.3302

397 C   Isotropic =   71.6115   Anisotropy =   173.7486

XX=   6.7271   YX=   42.5593   ZX=   2.6394

XY=   45.5852   YY=   79.5339   ZY=   -75.6614

XZ= 7.0974 YZ= -77.8054 ZZ= 128.5735

Eigenvalues: -25.6738 53.0644 187.4439

398 H Isotropic = 23.9326 Anisotropy = 2.4422

XX= 24.9337 YX= 0.2147 ZX= 1.0465

XY= -0.2931 YY= 23.6936 ZY= 0.8074

XZ= 1.3306 YZ= 0.3533 ZZ= 23.1705

Eigenvalues: 22.3551 23.8820 25.5607

399 H Isotropic = 24.8069 Anisotropy = 7.5863

XX= 18.1516 YX= -1.5595 ZX= 0.7496

XY= -1.1797 YY= 26.6862 ZY= -1.9453

XZ= 1.3715 YZ= 0.6968 ZZ= 29.5829

Eigenvalues: 17.8595 26.6967 29.8644

400 H Isotropic = 28.6282 Anisotropy = 6.4511

XX= 28.5901 YX= 0.0407 ZX= 2.4248

XY= -0.8335 YY= 29.6072 ZY= 2.3740

XZ= 6.9559 YZ= -0.0168 ZZ= 27.6872

Eigenvalues: 23.2226 29.7331 32.9289

401 H Isotropic = 27.9890 Anisotropy = 3.9968

XX= 29.2227 YX= 0.4573 ZX= -4.1960

XY= -1.0789 YY= 29.9927 ZY= 0.9199

XZ= -1.4746 YZ= -2.9718 ZZ= 24.7515

Eigenvalues: 23.2110 30.1024 30.6535

402 H Isotropic = 28.6241 Anisotropy = 7.0603

XX= 27.6885 YX= -1.5231 ZX= 2.4120

XY= -1.0661 YY= 27.7309 ZY= 1.1469

XZ= 5.1137 YZ= 3.3764 ZZ= 30.4528

Eigenvalues: 23.6548 28.8865 33.3309

403 H Isotropic = 24.0361 Anisotropy = 3.9509

XX= 26.3136 YX= -1.3067 ZX= -1.1121

XY= 1.6787 YY= 25.7976 ZY= 2.4385

XZ= 2.3003 YZ= 1.1397 ZZ= 19.9971

Eigenvalues: 19.4501 25.9882 26.6701

404 H Isotropic = 23.5860 Anisotropy = 7.0880

XX= 27.5611 YX= -1.8879 ZX= -1.2281

XY= -1.8192 YY= 21.3906 ZY= 1.4659

XZ= -0.5343 YZ= 1.3379 ZZ= 21.8064

Eigenvalues: 20.0723 22.3744 28.3114

405 H Isotropic = 24.0227 Anisotropy = 6.3337

XX= 24.8596 YX= 0.4836 ZX= 0.4850

XY= -1.2268 YY= 22.5427 ZY= 4.4015

XZ= 1.7154 YZ= 4.3819 ZZ= 24.6658

Eigenvalues: 18.9255 24.8975 28.2452

406 H Isotropic = 24.0356 Anisotropy = 5.8629

XX= 27.7127 YX= -0.5176 ZX= -0.4796

XY= -1.4666 YY= 21.7704 ZY= 0.7554

XZ= -0.6775 YZ= -0.2588 ZZ= 22.6236

Eigenvalues: 21.5854 22.5771 27.9442

407 H Isotropic = 24.2843 Anisotropy = 6.4914

XX= 25.3207 YX= -2.6683 ZX= -1.8063

XY= -2.5973 YY= 25.0215 ZY= 1.1827

XZ= -1.6925 YZ= 1.5598 ZZ= 22.5108

Eigenvalues: 21.6651 22.5759 28.6119

408 H Isotropic = 29.5809 Anisotropy = 5.3643

XX= 31.0783 YX= 1.3383 ZX= -3.4237

XY= 1.4174 YY= 28.8688 ZY= 0.6264

XZ= -2.4671 YZ= 1.6836 ZZ= 28.7955

Eigenvalues: 25.7930 29.7925 33.1571

409 O Isotropic = 305.8944 Anisotropy = 38.1480

XX= 291.0803 YX= -17.7087 ZX= 6.6157

XY= -20.0083 YY= 315.3040 ZY= -4.7261

XZ= 12.5738 YZ= -8.8630 ZZ= 311.2988

Eigenvalues: 279.8599 306.4969 331.3264

410 O Isotropic = 308.6928 Anisotropy = 33.3437

XX= 289.7758 YX= 9.7457 ZX= 6.9224

XY=   13.8072   YY=   323.8277   ZY=   6.1449

XZ=   8.5799   YZ=   5.3246   ZZ=   312.4749

Eigenvalues: 284.8635   310.2930   330.9219

411 H   Isotropic =   28.9001   Anisotropy =   24.5512

XX=   23.5019   YX=   7.4225   ZX=   -1.3676

XY=   8.4741   YY=   41.1496   ZY=   -4.3538

XZ=   -1.7278   YZ=   -5.1153   ZZ=   22.0489

Eigenvalues: 20.3531   21.0797   45.2676

412 H   Isotropic =   28.0608   Anisotropy =   23.9982

XX=   26.1505   YX=   -10.8398   ZX=   -1.4582

XY=   -9.9506   YY=   36.7278   ZY=   4.3098

XZ=   -1.7305   YZ=   4.6750   ZZ=   21.3041

Eigenvalues: 19.0507   21.0721   44.0596

413 H   Isotropic =   27.9994   Anisotropy =   18.3513

XX=   19.9865   YX=   -0.3310   ZX=   3.0003

XY=   0.4179   YY=   24.8775   ZY=   2.8791

XZ=   3.5537   YZ=   3.0192   ZZ=   39.1340

Eigenvalues: 19.4026   24.3619   40.2335

414 H   Isotropic =   29.4166   Anisotropy =   16.7229

XX=   22.8802   YX=   -0.7838   ZX=   3.2391

XY=   -0.3061   YY=   25.6421   ZY=   -0.7273

XZ=   3.9786   YZ=   -1.5092   ZZ=   39.7274

Eigenvalues: 22.1120   25.5725   40.5652

415 O   Isotropic =   283.8926   Anisotropy =   38.6199

XX=   260.1564   YX=   -0.5482   ZX=   -12.4409

XY=   -3.8640   YY=   291.4131   ZY=   11.0545

XZ=   -7.2893   YZ=   11.4856   ZZ=   300.1083

Eigenvalues: 257.8477   284.1910   309.6392

416 H   Isotropic =   28.6213   Anisotropy =   23.0376

XX=   23.2948   YX=   -7.7391   ZX=   -1.3462

XY=   -7.2835   YY=   40.5663   ZY=   4.1528

XZ=   -1.0981   YZ=   2.7119   ZZ=   22.0030

Eigenvalues: 20.4816 21.4027 43.9798

417 H Isotropic = 21.9521 Anisotropy = 29.9694

XX= 12.2782 YX= -3.0829 ZX= 0.6516

XY= -3.4222 YY= 14.4067 ZY= 8.7598

XZ= 0.3085 YZ= 8.6408 ZZ= 39.1714

Eigenvalues: 8.7024 15.2222 41.9317

418 O Isotropic = 303.6475 Anisotropy = 40.6467

XX= 301.2612 YX= 6.4758 ZX= -3.9209

XY= 4.8348 YY= 282.6672 ZY= 13.6199

XZ= -6.8610 YZ= 11.1659 ZZ= 327.0140

Eigenvalues: 277.4557 302.7413 330.7453

419 H Isotropic = 23.5872 Anisotropy = 33.0813

XX= 14.9916 YX= 0.1599 ZX= 0.4869

XY= -0.0135 YY= 17.7927 ZY= 15.1924

XZ= -0.1949 YZ= 14.0245 ZZ= 37.9771

Eigenvalues: 10.1294 14.9907 45.6414

420 H Isotropic = 30.7526 Anisotropy = 15.7720

XX= 35.1723 YX= 4.6871 ZX= -5.7794

XY= 5.4930 YY= 26.0579 ZY= -2.8926

XZ= -5.5311 YZ= -2.4550 ZZ= 31.0276

Eigenvalues: 23.7790 27.2116 41.2673

421 O Isotropic = 298.2319 Anisotropy = 29.9478

XX= 317.2830 YX= -8.8080 ZX= -2.8667

XY= -0.4155 YY= 280.0668 ZY= -6.3848

XZ= -3.7325 YZ= -3.8561 ZZ= 297.3458

Eigenvalues: 277.9406 298.5579 318.1971

422 H Isotropic = 30.8743 Anisotropy = 19.7721

XX= 35.6933 YX= -10.4215 ZX= 6.4386

XY= -4.0069 YY= 29.5250 ZY= -5.3839

XZ= 5.5913 YZ= -4.4747 ZZ= 27.4047

Eigenvalues: 23.4168 25.1505 44.0557

423 H Isotropic = 30.3774 Anisotropy = 22.2735

XX=   38.7501   YX=   1.0446   ZX=   -8.3942

XY=   8.9668   YY=   25.1437   ZY=   -5.2498

XZ=   -8.6587   YZ=   -2.5116   ZZ=   27.2385

Eigenvalues: 21.8940   24.0119   45.2264

424 O   Isotropic =   269.1469   Anisotropy =   18.1824

XX=   264.9015   YX=   0.8548   ZX=   -10.1027

XY=   0.9227   YY=   280.7269   ZY=   2.1527

XZ=   -10.0900   YZ=   4.0531   ZZ=   261.8123

Eigenvalues: 252.8337   273.3385   281.2685

425 H   Isotropic =   22.1338   Anisotropy =   33.0011

XX=   14.9381   YX=   6.9806   ZX=   -6.1444

XY=   7.1715   YY=   31.4131   ZY=   -14.4781

XZ=   -6.1380   YZ=   -14.2226   ZZ=   20.0501

Eigenvalues: 9.7894   12.4775   44.1345

426 H   Isotropic =   23.2892   Anisotropy =   31.9030

XX=   17.4745   YX=   -8.6265   ZX=   -5.2458

XY=   -7.8309   YY=   35.2893   ZY=   11.1253

XZ=   -5.3305   YZ=   12.4071   ZZ=   17.1039

Eigenvalues: 10.9557   14.3541   44.5579

427 O   Isotropic =   278.4939   Anisotropy =   41.0905

XX=   299.2653   YX=   -13.0255   ZX=   8.9994

XY=   -10.9249   YY=   274.6851   ZY=   -5.0952

XZ=   7.2590   YZ=   -1.7377   ZZ=   261.5313

Eigenvalues: 259.7775   269.8166   305.8875

428 H   Isotropic =   23.8616   Anisotropy =   35.8726

XX=   46.8948   YX=   1.4446   ZX=   -4.9414

XY=   2.6644   YY=   12.9532   ZY=   0.0231

XZ=   -5.5344   YZ=   0.0593   ZZ=   11.7367

Eigenvalues: 10.9140   12.8941   47.7766

429 H   Isotropic =   24.6994   Anisotropy =   28.9046

XX=   23.5005   YX=   -9.4622   ZX=   9.4550

XY=   -8.8291   YY=   26.4073   ZY=   -9.9604

XZ=   9.6583   YZ=   -10.2328   ZZ=   24.1905

Eigenvalues: 14.2109   15.9182   43.9691

430 C   Isotropic =   143.2572   Anisotropy =   41.7308

XX=   144.7143   YX=   7.3662   ZX=   -6.3002

XY=   16.6625   YY=   157.7863   ZY=   -11.0224

XZ=   -13.0787   YZ=   -15.6184   ZZ=   127.2709

Eigenvalues: 121.0062   137.6875   171.0777

431 C   Isotropic =   26.1051   Anisotropy =   128.3153

XX=   32.7893   YX=   -72.1670   ZX=   -12.7678

XY=   -78.5577   YY=   34.3144   ZY=   13.2824

XZ=   -17.9925   YZ=   2.8298   ZZ=   11.2115

Eigenvalues: -42.3495   9.0162   111.6486

432 O   Isotropic =   -40.7392   Anisotropy =   540.1510

XX=   66.4411   YX= -219.1120   ZX= -102.8744

XY= -224.2534   YY=   87.5208   ZY=   59.6014

XZ=   -98.1280   YZ=   53.0547   ZZ= -276.1795

Eigenvalues: -303.5382 -138.0409   319.3615

433 C   Isotropic =   156.8094   Anisotropy =   14.3648

XX=   156.5019   YX=   3.4093   ZX=   -1.7983

XY=   2.3344   YY=   163.7726   ZY=   -4.0497

XZ=   1.2062   YZ=   -6.5225   ZZ=   150.1537

Eigenvalues: 148.2881   155.7541   166.3859

434 C   Isotropic =   157.2835   Anisotropy =   29.4212

XX=   161.0854   YX=   7.4368   ZX=   -5.4635

XY=   7.5550   YY=   165.9196   ZY=   -13.4575

XZ=   -1.1232   YZ=   -13.9409   ZZ=   144.8454

Eigenvalues: 138.0935   156.8592   176.8976

435 C   Isotropic =   172.3695   Anisotropy =   16.2545

XX=   169.3582   YX=   2.4493   ZX=   -2.7637

XY=   -2.2329   YY=   166.4671   ZY=   1.8811

XZ=   -5.5254   YZ=   4.9418   ZZ=   181.2832

Eigenvalues: 165.3900   168.5126   183.2058

436 C Isotropic = 172.0255 Anisotropy = 23.0223

XX= 176.0573 YX= -12.3680 ZX= 4.7667

XY= -7.9020 YY= 172.8716 ZY= -4.8985

XZ= 6.6107 YZ= -4.3247 ZZ= 167.1476

Eigenvalues: 164.1187 164.5841 187.3737

437 H Isotropic = 30.3098 Anisotropy = 10.9344

XX= 31.4467 YX= 3.7516 ZX= -2.2576

XY= 4.0030 YY= 33.3411 ZY= -3.1514

XZ= -1.9878 YZ= -2.9973 ZZ= 26.1417

Eigenvalues: 24.9144 28.4156 37.5994

438 H Isotropic = 29.5049 Anisotropy = 5.4827

XX= 27.8030 YX= -0.0689 ZX= -4.7295

XY= -0.5197 YY= 31.1292 ZY= 0.4226

XZ= -2.9007 YZ= 1.7916 ZZ= 29.5824

Eigenvalues: 24.7417 30.6129 33.1600

439 H Isotropic = 29.9448 Anisotropy = 4.6153

XX= 31.4560 YX= -0.9595 ZX= 1.5605

XY= -0.5384 YY= 30.0542 ZY= -2.7207

XZ= -1.0726 YZ= -4.0193 ZZ= 28.3242

Eigenvalues: 25.6975 31.1153 33.0216

440 H Isotropic = 30.0166 Anisotropy = 3.9129

XX= 28.3629 YX= -0.2010 ZX= 2.1860

XY= -0.1616 YY= 29.9821 ZY= -0.8755

XZ= 1.5392 YZ= -0.0254 ZZ= 31.7048

Eigenvalues: 27.5315 29.8932 32.6252

441 H Isotropic = 30.3514 Anisotropy = 9.5132

XX= 30.6416 YX= -0.7780 ZX= -6.6124

XY= -0.1660 YY= 30.1747 ZY= -1.5714

XZ= -5.7066 YZ= -1.5895 ZZ= 30.2379

Eigenvalues: 23.9312 30.4295 36.6935

442 H Isotropic = 30.8169 Anisotropy = 8.2052

XX= 33.0274 YX= 1.9097 ZX= -3.9734

XY=   1.6361   YY=   31.9604   ZY=   -1.5345

XZ=   -4.2093   YZ=   -1.0587   ZZ=   27.4629

Eigenvalues: 25.2813   30.8824   36.2870

443 H   Isotropic =   30.3083   Anisotropy =   7.3100

XX=   30.8587   YX=   -2.3032   ZX=   -4.4754

XY=   -3.4763   YY=   31.3128   ZY=   -0.3058

XZ=   -3.8508   YZ=   -0.9307   ZZ=   28.7533

Eigenvalues: 24.6908   31.0524   35.1816

444 H   Isotropic =   30.6464   Anisotropy =   4.4485

XX=   32.6414   YX=   1.5490   ZX=   2.4058

XY=   1.3196   YY=   29.8941   ZY=   -1.9480

XZ=   1.4142   YZ=   -2.0005   ZZ=   29.4038

Eigenvalues: 26.7036   31.6236   33.6120

445 H   Isotropic =   30.6267   Anisotropy =   8.9508

XX=   30.6255   YX=   -4.6249   ZX=   -3.7585

XY=   -4.6177   YY=   32.9779   ZY=   -2.8270

XZ=   -3.3977   YZ=   -1.7480   ZZ=   28.2769

Eigenvalues: 23.3757   31.9106   36.5939

446 H   Isotropic =   30.9421   Anisotropy =   8.7467

XX=   32.1128   YX=   -2.2838   ZX=   2.1292

XY=   -3.2945   YY=   28.4727   ZY=   -3.7137

XZ=   2.2560   YZ=   -3.2981   ZZ=   32.2408

Eigenvalues: 26.0379   30.0152   36.7732

447 H   Isotropic =   29.8767   Anisotropy =   14.6552

XX=   25.0290   YX=   -0.5559   ZX=   2.9287

XY=   0.1661   YY=   25.7364   ZY=   0.2383

XZ=   3.7582   YZ=   0.8378   ZZ=   38.8645

Eigenvalues: 24.2002   25.7829   39.6468

448 N   Isotropic =   147.6758   Anisotropy =   102.6278

XX=   206.6738   YX=   20.6071   ZX=   12.9826

XY=   0.8382   YY=   170.2051   ZY=   22.6358

XZ=   17.2911   YZ=   73.0072   ZZ=   66.1485

Eigenvalues: 46.8505 180.0825 216.0943

449 C Isotropic = 143.3610 Anisotropy = 28.8540

XX= 143.6027 YX= -11.8368 ZX= -10.9329

XY= -10.1799 YY= 155.7894 ZY= -10.1959

XZ= -9.1158 YZ= -8.1710 ZZ= 130.6909

Eigenvalues: 120.1042 147.3817 162.5970

450 C Isotropic = 23.4374 Anisotropy = 127.4417

XX= 24.4500 YX= 68.8543 ZX= 27.7644

XY= 63.7748 YY= 28.8010 ZY= 22.8653

XZ= 40.2094 YZ= 15.3331 ZZ= 17.0614

Eigenvalues: -42.2580 4.1718 108.3986

451 O Isotropic = -28.5555 Anisotropy = 524.6692

XX= 55.2992 YX= 182.7194 ZX= 138.9703

XY= 173.5183 YY= 50.1887 ZY= 132.7807

XZ= 176.0503 YZ= 160.5463 ZZ= -191.1545

Eigenvalues: -281.6889 -125.2016 321.2239

452 H Isotropic = 28.1395 Anisotropy = 6.7466

XX= 31.4289 YX= 1.9763 ZX= 0.2633

XY= 2.6415 YY= 28.2244 ZY= -0.2943

XZ= -0.0415 YZ= 0.0159 ZZ= 24.7651

Eigenvalues: 24.7515 27.0298 32.6372

453 H Isotropic = 25.7746 Anisotropy = 4.1033

XX= 24.1913 YX= 0.9392 ZX= 2.0669

XY= 3.0488 YY= 26.3281 ZY= -2.8231

XZ= 0.3866 YZ= -0.9266 ZZ= 26.8045

Eigenvalues: 22.1003 26.7135 28.5102

454 H Isotropic = 26.5285 Anisotropy = 8.8039

XX= 28.5163 YX= -1.0012 ZX= -5.7307

XY= -2.4910 YY= 25.9645 ZY= 0.4967

XZ= -4.1497 YZ= -0.1762 ZZ= 25.1047

Eigenvalues: 21.4047 25.7831 32.3977

455 N Isotropic = 136.3530 Anisotropy = 107.9218

XX= 184.0385 YX= -14.1255 ZX= -6.0484

XY= -10.4299 YY= 133.0483 ZY= 55.6998

XZ= -39.5411 YZ= 83.1280 ZZ= 91.9721

Eigenvalues: 39.2766 161.4815 208.3008

456 C Isotropic = 128.4786 Anisotropy = 21.5729

XX= 127.9840 YX= 4.4394 ZX= 6.2793

XY= 7.1074 YY= 125.5007 ZY= 4.7521

XZ= 7.2838 YZ= 11.7285 ZZ= 131.9512

Eigenvalues: 119.6509 122.9245 142.8606

457 C Isotropic = 24.5439 Anisotropy = 123.5347

XX= 67.9167 YX= -48.5740 ZX= -45.6013

XY= -54.8085 YY= -6.2425 ZY= -16.0606

XZ= -37.5686 YZ= 0.7764 ZZ= 11.9575

Eigenvalues: -46.0701 12.8014 106.9004

458 O Isotropic = -51.8582 Anisotropy = 550.3945

XX= 199.4396 YX= -161.0701 ZX= -114.8648

XY= -146.0270 YY= -175.3834 ZY= 130.4239

XZ= -145.6884 YZ= 155.0929 ZZ= -179.6308

Eigenvalues: -320.8714 -149.7746 315.0715

459 C Isotropic = 149.9695 Anisotropy = 17.7366

XX= 141.9705 YX= 3.7044 ZX= -3.1777

XY= 1.8913 YY= 149.7574 ZY= 2.0989

XZ= -12.8227 YZ= 4.3913 ZZ= 158.1806

Eigenvalues: 137.4827 150.6320 161.7939

460 C Isotropic = 163.3663 Anisotropy = 19.6561

XX= 163.2523 YX= 9.4987 ZX= 1.0990

XY= 10.2164 YY= 159.2965 ZY= 8.8767

XZ= 2.6266 YZ= 6.7883 ZZ= 167.5501

Eigenvalues: 149.7261 163.9025 176.4703

461 C Isotropic = 176.9246 Anisotropy = 17.9931

XX= 181.9842 YX= -5.8829 ZX= 7.4932

XY= -6.5946 YY= 179.3057 ZY= -0.5811

XZ= 6.0878 YZ= -1.5119 ZZ= 169.4840

Eigenvalues: 166.2873 175.5665 188.9201

462 C Isotropic = 179.4130 Anisotropy = 25.6639

XX= 170.5548 YX= 4.3816 ZX= -12.2817

XY= 6.4387 YY= 175.4397 ZY= 0.2065

XZ= -8.7476 YZ= 2.9068 ZZ= 192.2446

Eigenvalues: 163.6364 178.0804 196.5223

463 H Isotropic = 30.6252 Anisotropy = 10.6029

XX= 33.3858 YX= -3.5973 ZX= -3.1584

XY= -3.2232 YY= 27.0466 ZY= 1.7586

XZ= -4.4191 YZ= 1.8754 ZZ= 31.4431

Eigenvalues: 25.5547 28.6271 37.6938

464 H Isotropic = 26.1024 Anisotropy = 8.8972

XX= 24.9489 YX= -5.1977 ZX= -3.7698

XY= -3.8942 YY= 28.1837 ZY= 0.6532

XZ= -2.6788 YZ= -0.3045 ZZ= 25.1746

Eigenvalues: 20.3140 25.9594 32.0338

465 H Isotropic = 27.8760 Anisotropy = 5.1715

XX= 27.5426 YX= 2.4233 ZX= -2.8369

XY= 1.8065 YY= 29.3946 ZY= -1.2641

XZ= -2.4924 YZ= 1.3777 ZZ= 26.6907

Eigenvalues: 24.0137 28.2905 31.3236

466 H Isotropic = 30.7397 Anisotropy = 3.4935

XX= 28.9543 YX= 0.2621 ZX= -3.3350

XY= -0.0091 YY= 33.0618 ZY= 0.5019

XZ= -2.2599 YZ= -0.4371 ZZ= 30.2031

Eigenvalues: 26.7102 32.4403 33.0687

467 H Isotropic = 30.4058 Anisotropy = 6.9442

XX= 32.8648 YX= -2.2462 ZX= -1.2804

XY= -1.3807 YY= 26.8886 ZY= 3.3321

XZ= -1.4395 YZ= 2.8882 ZZ= 31.4639

Eigenvalues: 25.1746 31.0075 35.0352

468 H Isotropic = 30.8867 Anisotropy = 6.8778

XX= 34.3791 YX= 0.3835 ZX= 1.8095

XY= -0.3647 YY= 28.2909 ZY= 3.9979

XZ= 1.8893 YZ= 4.1897 ZZ= 29.9902

Eigenvalues: 24.8151 32.3731 35.4719

469 H Isotropic = 31.1212 Anisotropy = 9.2604

XX= 26.9360 YX= 0.8872 ZX= -3.0996

XY= -0.1077 YY= 32.5312 ZY= -2.7256

XZ= -3.0496 YZ= -3.9147 ZZ= 33.8964

Eigenvalues: 25.6581 30.4106 37.2947

470 H Isotropic = 29.8155 Anisotropy = 13.6381

XX= 24.9815 YX= 5.9060 ZX= -2.9171

XY= 4.9290 YY= 36.1852 ZY= -1.2423

XZ= -2.8061 YZ= -1.5005 ZZ= 28.2799

Eigenvalues: 22.0185 28.5205 38.9076

471 H Isotropic = 30.5138 Anisotropy = 11.8067

XX= 34.2263 YX= -3.8239 ZX= -4.1129

XY= -3.6412 YY= 26.3226 ZY= 3.2049

XZ= -3.2688 YZ= 2.1473 ZZ= 30.9926

Eigenvalues: 24.5686 28.5880 38.3850

472 H Isotropic = 30.5986 Anisotropy = 10.8491

XX= 27.6786 YX= 1.6553 ZX= -0.0680

XY= 2.2981 YY= 28.1895 ZY= 3.9469

XZ= 1.0178 YZ= 4.2147 ZZ= 35.9277

Eigenvalues: 25.2745 28.6899 37.8314

473 H Isotropic = 30.8697 Anisotropy = 5.7256

XX= 29.2319 YX= -1.9681 ZX= -1.2479

XY= -0.5287 YY= 33.5253 ZY= -2.1816

XZ= -1.1552 YZ= -2.3696 ZZ= 29.8518

Eigenvalues: 27.3368 30.5854 34.6867

474 N Isotropic = 138.1527 Anisotropy = 107.6737

XX= 172.8275 YX= 35.1937 ZX= -15.4075

XY= 34.4567 YY= 174.0442 ZY= 66.1752

XZ= -41.8417 YZ= 26.8363 ZZ= 67.5863

Eigenvalues: 38.5003 166.0225 209.9351

475 C Isotropic = 136.2963 Anisotropy = 21.1469

XX= 140.7202 YX= -8.0357 ZX= -3.0115

XY= -4.1596 YY= 120.4046 ZY= 3.5103

XZ= -3.8236 YZ= 4.3802 ZZ= 147.7641

Eigenvalues: 118.4226 140.0721 150.3943

476 C Isotropic = 26.5839 Anisotropy = 114.8965

XX= -16.5265 YX= 32.2946 ZX= 36.5379

XY= 35.0147 YY= 89.7672 ZY= 9.8630

XZ= 25.1538 YZ= 10.2469 ZZ= 6.5110

Eigenvalues: -41.9073 18.4774 103.1816

477 O Isotropic = -28.8256 Anisotropy = 516.1218

XX= -119.0739 YX= 141.1555 ZX= -14.1544

XY= 150.4602 YY= 248.2653 ZY= 112.7335

XZ= -46.8581 YZ= 103.0942 ZZ= -215.6683

Eigenvalues: -271.2879 -130.4445 315.2556

478 C Isotropic = 173.6311 Anisotropy = 29.4103

XX= 166.8191 YX= 8.6798 ZX= -2.1663

XY= 12.8412 YY= 186.9477 ZY= -5.3786

XZ= -2.2988 YZ= -6.8668 ZZ= 167.1266

Eigenvalues: 162.1076 165.5478 193.2380

479 H Isotropic = 30.1076 Anisotropy = 8.5975

XX= 31.5763 YX= 3.4661 ZX= 2.6695

XY= 4.0489 YY= 29.2424 ZY= 2.0164

XZ= 2.4417 YZ= 1.4227 ZZ= 29.5041

Eigenvalues: 26.4654 28.0181 35.8393

480 H Isotropic = 26.7578 Anisotropy = 5.4116

XX= 25.3779 YX= 0.9567 ZX= 1.0298

XY= 1.4020 YY= 29.3425 ZY= 1.1059

XZ= 2.6532 YZ= 1.5307 ZZ= 25.5531

Eigenvalues: 23.6212 26.2866 30.3656

481 H Isotropic = 27.7918 Anisotropy = 5.7819

XX= 29.8112 YX= -2.1414 ZX= 3.2020

XY= -1.9601 YY= 26.6888 ZY= -0.9603

XZ= 0.2684 YZ= -0.9393 ZZ= 26.8754

Eigenvalues: 25.6585 26.0705 31.6464

482 H Isotropic = 30.0333 Anisotropy = 8.3271

XX= 32.6827 YX= -2.1194 ZX= 0.6027

XY= -3.7139 YY= 31.7222 ZY= -2.8347

XZ= 0.8494 YZ= -1.7194 ZZ= 25.6952

Eigenvalues: 24.9229 29.5924 35.5847

483 H Isotropic = 30.6898 Anisotropy = 10.9623

XX= 33.8842 YX= 2.2509 ZX= -4.8114

XY= 2.4268 YY= 28.1649 ZY= -2.0999

XZ= -4.8981 YZ= -1.1253 ZZ= 30.0202

Eigenvalues: 26.7264 27.3449 37.9979

484 N Isotropic = 136.5425 Anisotropy = 91.3012

XX= 187.6508 YX= -0.1129 ZX= -33.5793

XY= -17.5908 YY= 179.5200 ZY= 31.2438

XZ= 10.2811 YZ= 22.2127 ZZ= 42.4566

Eigenvalues: 36.7873 175.4302 197.4099

485 C Isotropic = 136.4067 Anisotropy = 19.3143

XX= 124.8133 YX= 3.6177 ZX= -10.9416

XY= 7.8646 YY= 143.1505 ZY= -1.8887

XZ= -7.4728 YZ= -3.1357 ZZ= 141.2563

Eigenvalues: 119.8989 140.0383 149.2829

486 C Isotropic = 25.9819 Anisotropy = 120.8335

XX= 102.9491 YX= -22.5361 ZX= 0.4313

XY= -23.1926 YY= -39.1750 ZY= 6.4693

XZ= -0.2265 YZ= -8.3571 ZZ= 14.1715

Eigenvalues: -42.7775 14.1856 106.5375

487 O Isotropic = -38.0993 Anisotropy = 529.5776

XX= 301.8872 YX= -71.0676 ZX= -27.0269

XY= -81.1062 YY= -128.4328 ZY= 5.5483

XZ= 19.6479 YZ= -22.4394 ZZ= -287.7523

Eigenvalues: -288.3057 -140.9446 314.9524

488 C Isotropic = 157.9690 Anisotropy = 18.4576

XX= 164.3014 YX= -9.7815 ZX= -2.6202

XY= -7.3853 YY= 156.7099 ZY= 0.5621

XZ= -2.4576 YZ= 1.0907 ZZ= 152.8956

Eigenvalues: 150.8392 152.7937 170.2740

489 C Isotropic = 59.7647 Anisotropy = 120.4634

XX= 17.3845 YX= 45.5725 ZX= 30.1146

XY= 42.5443 YY= 124.2514 ZY= -6.9921

XZ= 12.0567 YZ= -8.5686 ZZ= 37.6580

Eigenvalues: -9.2135 48.4339 140.0736

490 C Isotropic = 78.7463 Anisotropy = 111.9536

XX= 21.3593 YX= 47.0194 ZX= -6.0188

XY= 35.0934 YY= 140.4854 ZY= -1.4553

XZ= -4.4389 YZ= -1.6668 ZZ= 74.3943

Eigenvalues: 8.2697 74.5872 153.3821

491 N Isotropic = -1.4829 Anisotropy = 329.8529

XX= -104.1260 YX= 119.1456 ZX= 30.1569

XY= 130.1795 YY= 170.0093 ZY= -13.2120

XZ= 46.0943 YZ= -32.3637 ZZ= -70.3319

Eigenvalues: -171.3391 -51.5286 218.4190

492 C Isotropic = 70.7753 Anisotropy = 103.5481

XX= 23.5617 YX= 41.3550 ZX= -21.8658

XY= 52.7506 YY= 119.8245 ZY= -0.0890

XZ= -5.5202 YZ= -4.9397 ZZ= 68.9398

Eigenvalues: 2.3053 70.2133 139.8074

493 N Isotropic = 103.4113 Anisotropy = 131.5107

XX= 108.3780 YX= 37.1034 ZX= 10.0146

XY= 41.6236 YY= 170.5527 ZY= -20.7966

XZ=   16.2568   YZ=   -25.3822   ZZ=   31.3033

Eigenvalues: 22.7473   96.4015   191.0851

494 H   Isotropic =   22.0736   Anisotropy =   17.0509

XX=   18.4948   YX=   0.0916   ZX=   1.4949

XY=   3.0434   YY=   16.2829   ZY=   7.4962

XZ=   3.1121   YZ=   2.5881   ZZ=   31.4430

Eigenvalues: 14.5630   18.2168   33.4409

495 H   Isotropic =   23.4433   Anisotropy =   8.1488

XX=   23.3088   YX=   -4.0397   ZX=   0.8328

XY=   -2.8822   YY=   20.1335   ZY=   -5.5656

XZ=   -1.2987   YZ=   -1.9463   ZZ=   26.8875

Eigenvalues: 16.7999   24.6542   28.8758

496 H   Isotropic =   27.6303   Anisotropy =   5.0924

XX=   26.5551   YX=   -0.4158   ZX=   -0.0836

XY=   -1.0512   YY=   26.5072   ZY=   3.8470

XZ=   -0.3410   YZ=   0.6518   ZZ=   29.8287

Eigenvalues: 25.1429   26.7228   31.0253

497 H   Isotropic =   28.4303   Anisotropy =   6.2251

XX=   28.8158   YX=   -0.3838   ZX=   2.6926

XY=   -1.1089   YY=   27.6277   ZY=   4.7286

XZ=   1.6615   YZ=   3.0302   ZZ=   28.8472

Eigenvalues: 23.5423   29.1681   32.5803

498 H   Isotropic =   25.4256   Anisotropy =   8.8517

XX=   23.3922   YX=   -4.0950   ZX=   -2.9307

XY=   -3.3916   YY=   23.5285   ZY=   2.4798

XZ=   -0.5204   YZ=   2.0963   ZZ=   29.3561

Eigenvalues: 19.7003   25.2498   31.3267

499 H   Isotropic =   26.8987   Anisotropy =   10.0472

XX=   32.2838   YX=   -3.1700   ZX=   1.3266

XY=   -3.8667   YY=   23.4521   ZY=   -2.0287

XZ=   -0.8088   YZ=   -1.5477   ZZ=   24.9603

Eigenvalues: 21.4730   25.6263   33.5969

500 H   Isotropic =   28.3510   Anisotropy =   5.7673

XX=   26.5936   YX=   -1.4961   ZX=   -2.1711

XY=   -1.0009   YY=   29.8178   ZY=   -3.4544

XZ=   -1.3865   YZ=   -2.3493   ZZ=   28.6417

Eigenvalues: 24.2563   28.6009   32.1959

501 N   Isotropic =   138.4142   Anisotropy =   94.3356

XX=   176.2049   YX=   9.2199   ZX=   18.6449

XY=   -4.1077   YY=   174.6009   ZY=   34.2124

XZ=   16.2917   YZ=   77.0822   ZZ=   64.4367

Eigenvalues: 39.5373   174.4007   201.3046

502 C   Isotropic =   134.1364   Anisotropy =   25.9294

XX=   142.5776   YX=   -7.4576   ZX=   6.8435

XY=   -5.7983   YY=   133.6616   ZY=   -10.2337

XZ=   9.4165   YZ=   -7.7475   ZZ=   126.1700

Eigenvalues: 119.6438   131.3427   151.4227

503 C   Isotropic =   25.8820   Anisotropy =   117.6447

XX=   -44.9951   YX=   15.4930   ZX=   -9.1583

XY=   8.5409   YY=   85.1859   ZY=   33.7632

XZ=   5.9649   YZ=   36.1710   ZZ=   37.4552

Eigenvalues: -46.4002   19.7344   104.3118

504 O   Isotropic =   -58.9025   Anisotropy =   581.3305

XX= -169.9198   YX=   10.6551   ZX=   57.4378

XY=   -3.9904   YY=   181.6818   ZY=   251.2701

XZ=   66.0563   YZ=   295.1362   ZZ= -188.4694

Eigenvalues: -349.1780 -156.1806   328.6512

505 C   Isotropic =   151.9863   Anisotropy =   30.0600

XX=   142.3035   YX=   3.4239   ZX=   -5.1396

XY=   10.3941   YY=   169.3703   ZY=   -6.3506

XZ=   -6.9527   YZ=   -1.3904   ZZ=   144.2849

Eigenvalues: 136.8962   147.0363   172.0263

506 C   Isotropic =   165.1338   Anisotropy =   12.7924

XX=   160.9405   YX=   9.1004   ZX=   -0.2986

XY=   3.6076   YY=   170.3837   ZY=   1.5049

XZ=   1.4325   YZ=   -0.0700   ZZ=   164.0771

Eigenvalues: 157.7404   163.9988   173.6621

507 C   Isotropic =   169.7511   Anisotropy =   32.1962

XX=   184.2324   YX=   -0.6484   ZX=   -13.3755

XY=   1.5017   YY=   155.0843   ZY=   -3.5555

XZ=   -10.6576   YZ=   -4.2812   ZZ=   169.9367

Eigenvalues: 153.8391   164.1990   191.2153

508 C   Isotropic =   161.8420   Anisotropy =   30.0161

XX=   154.3543   YX=   2.5777   ZX=   -5.9890

XY=   0.9231   YY=   157.0328   ZY=   13.7194

XZ=   -11.4360   YZ=   9.5161   ZZ=   174.1388

Eigenvalues: 146.4678   157.2053   181.8527

509 H   Isotropic =   30.6890   Anisotropy =   7.6857

XX=   31.0113   YX=   0.4202   ZX=   3.0895

XY=   2.2610   YY=   28.0976   ZY=   1.9719

XZ=   2.9293   YZ=   1.6805   ZZ=   32.9580

Eigenvalues: 27.4324   28.8218   35.8127

510 H   Isotropic =   24.9872   Anisotropy =   4.3887

XX=   21.0856   YX=   0.3253   ZX=   -0.3637

XY=   1.4612   YY=   26.2626   ZY=   0.7923

XZ=   -1.7845   YZ=   -1.4068   ZZ=   27.6132

Eigenvalues: 20.7860   26.2625   27.9129

511 H   Isotropic =   28.8100   Anisotropy =   4.1788

XX=   29.9043   YX=   1.5493   ZX=   -3.0348

XY=   -1.3812   YY=   28.6886   ZY=   0.6309

XZ=   -1.8845   YZ=   1.0684   ZZ=   27.8372

Eigenvalues: 25.9897   28.8444   31.5958

512 H   Isotropic =   30.3065   Anisotropy =   6.0498

XX=   28.9087   YX=   3.2225   ZX=   -3.0687

XY=   3.4179   YY=   30.0853   ZY=   0.6721

XZ=   -2.9822   YZ=   0.4804   ZZ=   31.9257

Eigenvalues: 25.0309 31.5489 34.3398

513 H Isotropic = 29.8993 Anisotropy = 8.9421

XX= 31.5626 YX= -0.6018 ZX= 5.0549

XY= 0.9147 YY= 29.7300 ZY= 2.8490

XZ= 5.1755 YZ= 2.5674 ZZ= 28.4052

Eigenvalues: 23.8552 29.9820 35.8607

514 H Isotropic = 30.9803 Anisotropy = 8.2068

XX= 29.0280 YX= 1.4005 ZX= -0.9580

XY= 0.8207 YY= 33.2609 ZY= -3.7288

XZ= -1.9938 YZ= -3.9911 ZZ= 30.6522

Eigenvalues: 27.5961 28.8934 36.4515

515 H Isotropic = 30.4325 Anisotropy = 9.4309

XX= 27.6995 YX= -2.9582 ZX= 0.3546

XY= -3.0836 YY= 35.7069 ZY= -0.3662

XZ= 0.7492 YZ= 0.5432 ZZ= 27.8910

Eigenvalues: 26.4731 28.1046 36.7197

516 H Isotropic = 30.7559 Anisotropy = 4.5201

XX= 32.9364 YX= -0.3442 ZX= 1.4773

XY= -0.8430 YY= 30.0238 ZY= 0.6295

XZ= 2.1822 YZ= -0.4463 ZZ= 29.3075

Eigenvalues: 28.4796 30.0188 33.7694

517 H Isotropic = 32.1653 Anisotropy = 11.2994

XX= 39.4566 YX= -2.0227 ZX= 1.8761

XY= -0.0410 YY= 28.4907 ZY= 0.3795

XZ= 0.7307 YZ= 0.1701 ZZ= 28.5486

Eigenvalues: 28.0067 28.7910 39.6982

518 H Isotropic = 31.0313 Anisotropy = 7.8779

XX= 30.1051 YX= -0.5951 ZX= -2.8241

XY= -1.3093 YY= 28.9079 ZY= 2.1357

XZ= -3.5662 YZ= 0.8668 ZZ= 34.0808

Eigenvalues: 28.2797 28.5309 36.2832

519 H Isotropic = 30.6348 Anisotropy = 7.3653

XX=   29.1245   YX=   -0.7256   ZX=   -1.2566

XY=   1.4793   YY=   29.1742   ZY=   2.2277

XZ=   -2.2623   YZ=   4.0619   ZZ=   33.6057

Eigenvalues: 26.8893   29.4702   35.5450

520 N   Isotropic =   135.8814   Anisotropy =   97.1156

XX=   188.3501   YX=   8.8083   ZX=   -14.5273

XY=   20.0047   YY=   136.8737   ZY=   64.1303

XZ=   -59.6597   YZ=   56.7834   ZZ=   82.4205

Eigenvalues: 33.5045   173.5146   200.6252

521 C   Isotropic =   136.5697   Anisotropy =   23.5484

XX=   127.9649   YX=   -6.3066   ZX=   12.9498

XY=   -4.5855   YY=   138.3974   ZY=   6.9412

XZ=   14.5708   YZ=   6.7911   ZZ=   143.3467

Eigenvalues: 116.7635   140.6769   152.2686

522 C   Isotropic =   27.0450   Anisotropy =   114.2852

XX=   96.4130   YX=   22.2059   ZX=   -3.9433

XY=   23.7307   YY=   -19.4012   ZY=   -45.0137

XZ=   -11.2939   YZ=   -31.8291   ZZ=   4.1233

Eigenvalues: -49.1994   27.0993   103.2351

523 O   Isotropic =   -55.3515   Anisotropy =   577.9322

XX=   257.8663   YX=   143.7752   ZX= -106.4020

XY=   168.1667   YY= -187.8374   ZY=   44.3468

XZ= -163.1251   YZ=   63.1475   ZZ= -236.0833

Eigenvalues: -338.5641 -157.4270   329.9366

524 C   Isotropic =   174.9799   Anisotropy =   25.0185

XX=   184.6703   YX=   0.5858   ZX=   -9.0525

XY=   4.3082   YY=   164.4492   ZY=   -4.1038

XZ=   -10.5606   YZ=   -3.8286   ZZ=   175.8202

Eigenvalues: 163.1765   170.1043   191.6589

525 H   Isotropic =   30.3851   Anisotropy =   12.4883

XX=   37.4479   YX=   -3.9695   ZX=   1.7883

XY=   -3.2105   YY=   25.9597   ZY=   -0.9255

XZ= 1.0738 YZ= -0.6891 ZZ= 27.7478

Eigenvalues: 24.8759 27.5688 38.7107

526 H Isotropic = 24.6099 Anisotropy = 4.9957

XX= 27.1158 YX= -0.8072 ZX= -0.4624

XY= -0.4363 YY= 21.2710 ZY= -2.6297

XZ= 1.1549 YZ= -4.4859 ZZ= 25.4430

Eigenvalues: 19.2156 26.6738 27.9404

527 H Isotropic = 27.7841 Anisotropy = 6.5683

XX= 28.7241 YX= -1.7266 ZX= 2.3880

XY= -2.4054 YY= 29.8586 ZY= -3.1884

XZ= 0.3566 YZ= -0.5583 ZZ= 24.7697

Eigenvalues: 24.0400 27.1494 32.1630

528 H Isotropic = 30.0168 Anisotropy = 5.2556

XX= 30.4338 YX= -0.3492 ZX= -1.1832

XY= -2.0403 YY= 32.3687 ZY= -2.2034

XZ= -0.8138 YZ= -2.6200 ZZ= 27.2479

Eigenvalues: 25.8733 30.6565 33.5205

529 H Isotropic = 30.7658 Anisotropy = 7.8371

XX= 30.8012 YX= -2.6921 ZX= -1.4836

XY= -2.7066 YY= 27.9122 ZY= 2.6774

XZ= -1.2874 YZ= 3.2547 ZZ= 33.5841

Eigenvalues: 25.7925 30.5144 35.9906

530 N Isotropic = 140.4114 Anisotropy = 91.4708

XX= 166.3117 YX= 9.5921 ZX= -38.6183

XY= 8.6740 YY= 193.7303 ZY= 55.2104

XZ= -29.5103 YZ= 10.1091 ZZ= 61.1923

Eigenvalues: 43.4668 176.3755 201.3920

531 C Isotropic = 137.8696 Anisotropy = 25.6466

XX= 143.2871 YX= 4.9744 ZX= -6.5952

XY= 8.6372 YY= 119.0224 ZY= -0.3959

XZ= -6.3176 YZ= 4.3573 ZZ= 151.2993

Eigenvalues: 116.8652 141.7762 154.9674

532 C   Isotropic =   26.7278   Anisotropy =   118.8912

XX=   -22.7843   YX=   -50.4939   ZX=   19.0752

XY=   -43.9055   YY=   86.3507   ZY=   21.3227

XZ=   5.7668   YZ=   16.4958   ZZ=   16.6168

Eigenvalues: -45.7999   19.9946   105.9886

533 O   Isotropic =   -26.4588   Anisotropy =   544.2896

XX=   -89.7739   YX= -152.8262   ZX=   -61.0627

XY= -142.8502   YY=   271.6735   ZY=   91.8936

XZ=   -83.6540   YZ=   35.3247   ZZ= -261.2759

Eigenvalues: -287.9071 -127.8701   336.4010

534 C   Isotropic =   173.3341   Anisotropy =   23.2142

XX=   164.2589   YX=   -6.4215   ZX=   -0.1617

XY=   -3.5551   YY=   187.7488   ZY=   -0.3894

XZ=   -0.3096   YZ=   -1.7040   ZZ=   167.9946

Eigenvalues: 163.2028   167.9892   188.8102

535 H   Isotropic =   30.8557   Anisotropy =   6.2085

XX=   31.4101   YX=   3.0722   ZX=   -0.9832

XY=   2.5434   YY=   30.7526   ZY=   -1.4542

XZ=   -2.7738   YZ=   -1.0311   ZZ=   30.4044

Eigenvalues: 28.1789   29.3935   34.9947

536 H   Isotropic =   24.2187   Anisotropy =   7.4859

XX=   20.2014   YX=   -2.3682   ZX=   1.6149

XY=   -2.8603   YY=   24.1525   ZY=   -1.8317

XZ=   3.1221   YZ=   0.9951   ZZ=   28.3020

Eigenvalues: 18.5066   24.9402   29.2093

537 H   Isotropic =   27.7810   Anisotropy =   7.6618

XX=   30.5445   YX=   -1.3289   ZX=   4.9752

XY=   -2.3522   YY=   25.2090   ZY=   2.0781

XZ=   1.8923   YZ=   0.7177   ZZ=   27.5894

Eigenvalues: 23.0571   27.3970   32.8888

538 H   Isotropic =   30.3243   Anisotropy =   10.9174

XX=   27.3808   YX=   -0.5108   ZX=   0.8210

XY= -2.3009 YY= 31.7536 ZY= 5.5137

XZ= -2.7077 YZ= 5.5594 ZZ= 31.8386

Eigenvalues: 26.1468 27.2235 37.6026

539 H Isotropic = 31.1682 Anisotropy = 6.4105

XX= 32.9116 YX= -1.5045 ZX= 4.0523

XY= -4.0769 YY= 30.0580 ZY= 0.5915

XZ= 1.7206 YZ= 1.9171 ZZ= 30.5349

Eigenvalues: 26.5152 31.5475 35.4418

540 N Isotropic = 147.0969 Anisotropy = 98.9567

XX= 209.5673 YX= 8.9139 ZX= 2.3533

XY= -9.3850 YY= 175.3475 ZY= 11.4051

XZ= 42.9779 YZ= 30.3722 ZZ= 56.3761

Eigenvalues: 49.6797 178.5430 213.0680

541 C Isotropic = 144.9051 Anisotropy = 37.9741

XX= 163.9321 YX= 11.5376 ZX= -20.8094

XY= 2.4699 YY= 135.0477 ZY= -4.2521

XZ= -5.2446 YZ= 4.6057 ZZ= 135.7356

Eigenvalues: 129.1253 135.3689 170.2212

542 C Isotropic = 164.8476 Anisotropy = 21.2507

XX= 176.9077 YX= 5.1510 ZX= -6.0184

XY= 9.0656 YY= 151.3959 ZY= -8.6827

XZ= 6.6621 YZ= -7.0976 ZZ= 166.2392

Eigenvalues: 146.5159 169.0122 179.0147

543 C Isotropic = 76.0418 Anisotropy = 127.7880

XX= 87.0772 YX= -11.0310 ZX= 64.8576

XY= -13.8801 YY= 29.1155 ZY= 25.0782

XZ= 54.3347 YZ= 21.9888 ZZ= 111.9327

Eigenvalues: 8.4776 58.4140 161.2338

544 C Isotropic = 80.0800 Anisotropy = 89.6124

XX= 104.3718 YX= -29.9670 ZX= 32.5987

XY= -16.3666 YY= 23.6824 ZY= 22.6165

XZ= 30.0011 YZ= 18.1721 ZZ= 112.1858

Eigenvalues: 9.5116 90.9069 139.8216

545 C Isotropic = 67.9247 Anisotropy = 161.1806

XX= 71.5298 YX= -1.2722 ZX= 83.5794

XY= 0.4885 YY= 19.2817 ZY= 15.3109

XZ= 75.0521 YZ= 19.1593 ZZ= 112.9626

Eigenvalues: 2.6988 25.6969 175.3785

546 C Isotropic = 61.2922 Anisotropy = 140.6577

XX= 80.4935 YX= 30.9762 ZX= 58.3827

XY= 25.7532 YY= 3.3685 ZY= -8.7859

XZ= 67.0177 YZ= -2.6742 ZZ= 100.0147

Eigenvalues: -13.6199 42.4326 155.0640

547 C Isotropic = 78.1507 Anisotropy = 164.3121

XX= 64.3166 YX= 25.2335 ZX= 97.2248

XY= 22.7800 YY= 58.3189 ZY= -2.6147

XZ= 93.5778 YZ= -2.1049 ZZ= 111.8166

Eigenvalues: -15.8992 62.6592 187.6921

548 N Isotropic = 140.5717 Anisotropy = 65.4121

XX= 134.3082 YX= 11.1724 ZX= 49.2409

XY= 19.4345 YY= 140.5515 ZY= -17.5463

XZ= 36.6688 YZ= -15.9514 ZZ= 146.8554

Eigenvalues: 87.5755 149.9598 184.1798

549 C Isotropic = 86.4433 Anisotropy = 142.5901

XX= 72.4232 YX= 3.8142 ZX= 87.3934

XY= 4.4399 YY= 74.3464 ZY= 6.6012

XZ= 84.5285 YZ= 9.5685 ZZ= 112.5605

Eigenvalues: 4.1732 73.6533 181.5034

550 C Isotropic = 75.1025 Anisotropy = 161.0822

XX= 97.1947 YX= 39.0908 ZX= 64.3803

XY= 38.3665 YY= -1.0218 ZY= -11.4447

XZ= 68.0061 YZ= -12.3466 ZZ= 129.1344

Eigenvalues: -23.0327 65.8495 182.4906

551 C Isotropic = 72.2848 Anisotropy = 169.8904

XX= 89.7815 YX= -21.7377 ZX= 79.2174

XY= -20.7674 YY= 9.5297 ZY= 33.8449

XZ= 80.0903 YZ= 36.1145 ZZ= 117.5432

Eigenvalues: -23.6570 54.9663 185.5450

552 H Isotropic = 23.9823 Anisotropy = 3.1014

XX= 23.4454 YX= -1.4181 ZX= -1.1019

XY= -1.6423 YY= 25.1082 ZY= -1.0092

XZ= -0.6729 YZ= -0.6679 ZZ= 23.3934

Eigenvalues: 21.6926 24.2044 26.0499

553 H Isotropic = 25.1907 Anisotropy = 6.3687

XX= 26.9240 YX= 1.4981 ZX= 1.8535

XY= 2.4175 YY= 19.4328 ZY= 0.0663

XZ= -1.1250 YZ= 1.7566 ZZ= 29.2151

Eigenvalues: 18.8901 27.2455 29.4364

554 H Isotropic = 28.3545 Anisotropy = 4.2881

XX= 29.8943 YX= 1.3557 ZX= -2.2432

XY= 0.8286 YY= 27.8598 ZY= 1.4333

XZ= 0.1486 YZ= 5.7872 ZZ= 27.3095

Eigenvalues: 23.6013 30.2490 31.2133

555 H Isotropic = 28.0856 Anisotropy = 5.7277

XX= 29.7468 YX= 0.1368 ZX= -0.8199

XY= -0.2352 YY= 29.9765 ZY= -5.0452

XZ= 2.3549 YZ= -2.3151 ZZ= 24.5335

Eigenvalues: 22.6161 29.7367 31.9041

556 H Isotropic = 28.4548 Anisotropy = 6.6456

XX= 27.0478 YX= 0.2897 ZX= -0.5107

XY= 0.8337 YY= 27.8226 ZY= 1.8113

XZ= -3.9211 YZ= 4.1878 ZZ= 30.4941

Eigenvalues: 24.5752 27.9040 32.8852

557 H Isotropic = 24.0279 Anisotropy = 5.5259

XX= 25.6565 YX= -2.9296 ZX= -2.3274

XY= -0.2873 YY= 25.8502 ZY= -1.3995

XZ= -1.8800 YZ= 1.5731 ZZ= 20.5771

Eigenvalues: 19.7813 24.5905 27.7119

558 H Isotropic = 23.7226 Anisotropy = 8.5916

XX= 21.6980 YX= 0.4771 ZX= -0.9653

XY= 0.6881 YY= 28.4020 ZY= -3.1262

XZ= -1.2492 YZ= -2.4559 ZZ= 21.0677

Eigenvalues: 19.7307 21.9867 29.4503

559 H Isotropic = 24.0061 Anisotropy = 6.4722

XX= 22.7198 YX= -0.0170 ZX= -4.3948

XY= -2.1883 YY= 25.7751 ZY= -0.0525

XZ= -5.1052 YZ= 0.8593 ZZ= 23.5233

Eigenvalues: 18.3151 25.3822 28.3209

560 H Isotropic = 23.9868 Anisotropy = 5.8962

XX= 21.9793 YX= 0.2049 ZX= -0.6931

XY= -0.5510 YY= 27.3023 ZY= -1.7737

XZ= -0.1837 YZ= -1.8166 ZZ= 22.6788

Eigenvalues: 21.5487 22.4942 27.9176

561 H Isotropic = 24.2763 Anisotropy = 5.0438

XX= 24.4987 YX= 0.3810 ZX= -1.3228

XY= 0.5246 YY= 25.3679 ZY= -2.9759

XZ= -1.5851 YZ= -2.5478 ZZ= 22.9622

Eigenvalues: 20.8772 24.3128 27.6388

562 H Isotropic = 29.6139 Anisotropy = 5.5800

XX= 30.1806 YX= -1.2434 ZX= 0.1545

XY= -2.1286 YY= 29.6789 ZY= -4.3373

XZ= -1.5676 YZ= -3.2144 ZZ= 28.9822

Eigenvalues: 25.0004 30.5074 33.3339

563 O Isotropic = 280.5056 Anisotropy = 51.9100

XX= 278.0713 YX= 2.8879 ZX= -17.2318

XY= 5.5011 YY= 260.3112 ZY= -17.4670

XZ= -15.5133 YZ= -10.9229 ZZ= 303.1343

Eigenvalues: 256.0029 270.4016 315.1123

564 H Isotropic = 27.6394 Anisotropy = 23.1733

XX= 41.4408 YX= 4.9638 ZX= -3.5386

XY= 5.8235 YY= 20.4958 ZY= -1.0955

XZ= -1.6145 YZ= -0.9209 ZZ= 20.9815

Eigenvalues: 19.0996 20.7303 43.0882

565 H Isotropic = 22.0410 Anisotropy = 30.5737

XX= 13.7511 YX= 3.5649 ZX= -8.3287

XY= 3.8013 YY= 12.8642 ZY= -2.5985

XZ= -8.2917 YZ= -2.9485 ZZ= 39.5077

Eigenvalues: 9.0671 14.6325 42.4235

566 O Isotropic = 308.7898 Anisotropy = 41.2750

XX= 317.5422 YX= 8.4861 ZX= 14.1025

XY= 4.5674 YY= 298.9746 ZY= 12.9291

XZ= 15.3202 YZ= 22.5808 ZZ= 309.8524

Eigenvalues: 285.3661 304.6968 336.3065

567 H Isotropic = 24.5101 Anisotropy = 33.7054

XX= 38.5987 YX= 3.5556 ZX= 13.9417

XY= 2.5637 YY= 13.3182 ZY= 2.7763

XZ= 13.9887 YZ= 3.5567 ZZ= 21.6133

Eigenvalues: 11.9333 14.6167 46.9803

568 H Isotropic = 30.8414 Anisotropy = 17.2190

XX= 25.2016 YX= 0.5957 ZX= 1.1552

XY= 1.9176 YY= 37.5344 ZY= 7.5518

XZ= 1.7202 YZ= 7.5030 ZZ= 29.7881

Eigenvalues: 24.5244 25.6790 42.3207

DFT based structure:

Standard orientation:

Center Number	Atomic Number	Atomic Type	Coordinates (Angstroms) X	Y	Z
1	6	0	-3.119568	6.983613	-5.020523

2	6	0	-2.012136	6.174667	-4.350187
3	8	0	-1.730711	6.355436	-3.157780
4	6	0	-4.451653	6.210447	-5.075496
5	6	0	-5.589960	7.146541	-5.483933
6	6	0	-4.752787	5.559618	-3.755616
7	6	0	-6.886059	6.389461	-5.785219
8	1	0	-7.709654	7.083257	-5.983988
9	1	0	-2.820299	7.291319	-6.030536
10	1	0	-3.245116	7.886773	-4.416821
11	1	0	-4.353019	5.437646	-5.856278
12	1	0	-5.302823	7.729649	-6.369176
13	1	0	-3.973252	4.863136	-3.434009
14	1	0	-7.192029	5.749755	-4.956995
15	1	0	-5.755902	7.873573	-4.677621
16	1	0	-5.680567	4.983730	-3.787855
17	1	0	-6.754934	5.781462	-6.682492
18	1	0	-4.860086	6.316222	-3.001982
19	7	0	-1.381394	5.263683	-5.098517
20	6	0	-0.267299	4.500552	-4.548182
21	6	0	0.900930	5.378249	-4.132644
22	8	0	1.546130	5.125408	-3.110714
23	1	0	0.097742	3.771747	-5.278782
24	1	0	-1.696068	5.092388	-6.041896
25	1	0	-0.585420	3.942486	-3.664972
26	7	0	1.187736	6.428426	-4.905567
27	6	0	2.267338	7.339861	-4.514776
28	6	0	1.888568	8.104913	-3.245588
29	8	0	2.703168	8.245752	-2.324177
30	6	0	2.630693	8.281586	-5.677721
31	6	0	3.272972	7.479686	-6.824960
32	6	0	3.562181	9.382739	-5.194015
33	6	0	3.112570	8.126701	-8.209601

34	1	0	3.535190	9.136424	-8.239482
35	1	0	0.679931	6.583967	-5.763994
36	1	0	3.135866	6.742646	-4.229950
37	1	0	1.696075	8.742514	-6.037626
38	1	0	2.840604	6.471531	-6.863732
39	1	0	3.072049	10.066924	-4.496645
40	1	0	2.056526	8.204415	-8.493263
41	1	0	4.324958	7.348078	-6.597329
42	1	0	3.924370	9.979326	-6.036733
43	1	0	3.619216	7.536779	-8.979951
44	1	0	4.432564	8.965884	-4.678409
45	7	0	0.633328	8.562329	-3.149086
46	6	0	0.181274	9.242049	-1.935107
47	6	0	0.212794	8.312598	-0.730767
48	8	0	0.643151	8.705722	0.364063
49	6	0	-1.233522	9.788943	-2.138894
50	1	0	-1.935284	8.978178	-2.355412
51	1	0	-0.054847	8.281872	-3.831238
52	1	0	0.880168	10.049032	-1.706804
53	1	0	-1.255636	10.513646	-2.959074
54	1	0	-1.566756	10.294334	-1.229459
55	7	0	-0.278028	7.081700	-0.907434
56	6	0	-0.255000	6.082099	0.159095
57	6	0	1.143437	5.937462	0.753738
58	8	0	1.313023	5.942956	1.978349
59	6	0	-0.778688	4.745165	-0.383811
60	6	0	-1.185233	3.766443	0.678514
61	6	0	-1.120094	3.832087	2.031435
62	7	0	-1.738929	2.539537	0.382066
63	6	0	-1.981265	1.884713	1.506071
64	7	0	-1.614030	2.647005	2.520630
65	1	0	-1.880007	2.520281	3.499151

66	1	0	-0.645873	6.799300	-1.809248
67	1	0	-0.882977	6.428681	0.985669
68	1	0	-0.026646	4.291550	-1.040631
69	1	0	-0.747640	4.596765	2.696368
70	1	0	-2.472693	0.930753	1.589309
71	1	0	-1.651238	4.940472	-1.017250
72	7	0	2.169124	5.847191	-0.097022
73	6	0	3.528767	5.748207	0.434782
74	6	0	3.924684	7.007156	1.197065
75	8	0	4.607989	6.927911	2.227837
76	6	0	4.545867	5.479374	-0.678241
77	6	0	5.978008	5.250812	-0.147088
78	6	0	6.040408	4.095827	0.855038
79	6	0	6.973662	4.989822	-1.253221
80	1	0	6.780494	4.034480	-1.751394
81	1	0	2.017709	5.798910	-1.099550
82	1	0	3.545413	4.942391	1.169181
83	1	0	4.533809	6.321381	-1.381513
84	1	0	6.286525	6.161324	0.380612
85	1	0	7.080303	3.879044	1.124404
86	1	0	7.986865	4.933888	-0.837430
87	1	0	4.217879	4.591738	-1.237661
88	1	0	5.607437	3.190862	0.431111
89	1	0	6.981471	5.791514	-1.998833
90	1	0	5.515080	4.331732	1.781719
91	7	0	3.532716	8.182556	0.694512
92	6	0	3.860532	9.419501	1.397510
93	6	0	3.238884	9.421601	2.789666
94	8	0	3.893306	9.759279	3.785185
95	6	0	3.409297	10.634373	0.573351
96	1	0	3.690593	11.552539	1.095581
97	1	0	2.984842	8.225484	-0.155100

98	1	0	4.938729	9.456053	1.574944
99	1	0	2.325065	10.625919	0.437444
100	1	0	3.889971	10.632242	-0.410609
101	7	0	1.959765	9.055175	2.876449
102	6	0	1.288808	9.002026	4.169793
103	6	0	1.948420	7.990119	5.095302
104	8	0	2.102585	8.238449	6.299181
105	6	0	-0.180624	8.650854	3.964401
106	1	0	-0.282137	7.662176	3.509133
107	1	0	1.452935	8.762978	2.050486
108	1	0	1.397201	9.964158	4.677708
109	1	0	-0.669286	9.386644	3.316191
110	1	0	-0.696585	8.641408	4.924952
111	7	0	2.304868	6.824126	4.558465
112	6	0	2.966960	5.805034	5.369078
113	6	0	3.164552	4.535909	4.540730
114	6	0	3.629850	3.345972	5.356802
115	6	0	2.845323	2.384978	5.917029
116	6	0	4.978905	3.017441	5.695519
117	6	0	4.939319	1.827858	6.462861
118	6	0	6.219872	3.603154	5.417153
119	7	0	3.624362	1.463513	6.589104
120	6	0	6.091260	1.221984	6.964177
121	6	0	7.369551	2.999229	5.918800
122	6	0	7.293559	1.822662	6.684113
123	1	0	8.205739	1.360695	7.049436
124	1	0	2.255071	6.683169	3.558396
125	1	0	2.347361	5.597365	6.247980
126	1	0	3.926031	6.182003	5.744218
127	1	0	3.876538	4.749101	3.736046
128	1	0	1.768381	2.303914	5.942322
129	1	0	6.282298	4.517695	4.834064

130	1	0	3.257059	0.714805	7.153715
131	1	0	6.048559	0.304031	7.542210
132	1	0	8.339472	3.445385	5.720682
133	1	0	2.207729	4.303943	4.056843
134	8	0	2.305951	1.857900	-2.379869
135	1	0	2.231918	1.960555	-1.403145
136	1	0	1.697508	1.143588	-2.624295
137	8	0	0.509927	0.325036	6.660863
138	1	0	-0.183481	-0.276352	6.957542
139	1	0	0.456867	0.259957	5.682808
140	6	0	-7.307549	-3.027573	-4.863865
141	6	0	-6.441134	-1.968605	-4.190626
142	8	0	-6.553725	-1.727143	-2.980949
143	6	0	-6.571789	-4.377583	-4.978086
144	6	0	-7.585866	-5.516200	-4.937055
145	6	0	-5.502341	-4.507415	-3.904709
146	6	0	-7.106071	-6.759754	-5.628094
147	1	0	-7.865022	-7.549096	-5.615294
148	1	0	-7.652253	-2.689460	-5.847478
149	1	0	-8.182773	-3.144812	-4.219397
150	1	0	-6.075364	-4.399322	-5.960464
151	1	0	-8.515762	-5.187063	-5.421544
152	1	0	-4.670893	-3.812580	-4.049958
153	1	0	-6.213543	-7.172162	-5.146368
154	1	0	-7.851352	-5.725915	-3.892703
155	1	0	-5.076674	-5.516413	-3.904901
156	1	0	-6.857107	-6.562369	-6.677588
157	1	0	-5.909803	-4.328089	-2.913815
158	7	0	-5.544042	-1.344436	-4.940482
159	6	0	-4.779581	-0.239754	-4.376473
160	6	0	-5.647829	0.952572	-3.999755
161	8	0	-5.407697	1.614412	-2.987366

162	1	0	-4.035660	0.065466	-5.117440
163	1	0	-5.261959	-1.675675	-5.872442
164	1	0	-4.243479	-0.550446	-3.479800
165	7	0	-6.685049	1.233014	-4.786167
166	6	0	-7.620303	2.287439	-4.380231
167	6	0	-8.316359	1.888185	-3.084571
168	8	0	-8.393698	2.677915	-2.128575
169	6	0	-8.617470	2.600526	-5.510613
170	6	0	-7.872799	3.157789	-6.732700
171	6	0	-9.671065	3.633452	-5.058419
172	6	0	-8.728148	3.273602	-7.997632
173	1	0	-8.119757	3.601698	-8.846430
174	1	0	-6.868155	0.657221	-5.596042
175	1	0	-7.044618	3.181030	-4.130272
176	1	0	-9.132550	1.667123	-5.786611
177	1	0	-7.010163	2.518839	-6.966423
178	1	0	-10.241865	3.292136	-4.190836
179	1	0	-9.542511	3.994617	-7.886463
180	1	0	-7.460747	4.126039	-6.467262
181	1	0	-10.383083	3.831896	-5.861775
182	1	0	-9.173597	2.309068	-8.267372
183	1	0	-9.195331	4.579092	-4.783481
184	7	0	-8.762711	0.621129	-3.003851
185	6	0	-9.393474	0.139341	-1.781585
186	6	0	-8.433356	0.211663	-0.604355
187	8	0	-8.831505	0.560386	0.514877
188	6	0	-9.896435	-1.299270	-1.976195
189	1	0	-10.631202	-1.344981	-2.786507
190	1	0	-8.559593	-0.035275	-3.741405
191	1	0	-10.223694	0.802231	-1.526246
192	1	0	-10.379573	-1.643038	-1.058890
193	1	0	-9.063392	-1.970724	-2.200461

194	7	0	-7.166461	-0.155624	-0.832120
195	6	0	-6.183063	-0.194222	0.247516
196	6	0	-6.022691	1.178500	0.902494
197	8	0	-6.009921	1.295580	2.131428
198	6	0	-4.845773	-0.712438	-0.303976
199	6	0	-3.832174	-1.050966	0.745361
200	6	0	-3.855151	-0.913925	2.091972
201	7	0	-2.608104	-1.608055	0.437592
202	6	0	-1.920218	-1.802064	1.549311
203	7	0	-2.654450	-1.389513	2.566153
204	1	0	-2.436438	-1.510737	3.550229
205	1	0	-6.903153	-0.562417	-1.724893
206	1	0	-6.545090	-0.849734	1.045689
207	1	0	-4.422648	0.028749	-0.991876
208	1	0	-4.609086	-0.525846	2.759415
209	1	0	-0.942906	-2.250325	1.627136
210	1	0	-5.034570	-1.606045	-0.908057
211	7	0	-5.916592	2.235497	0.104777
212	6	0	-5.796227	3.565743	0.710838
213	6	0	-7.072982	3.967016	1.456238
214	8	0	-7.003117	4.545138	2.547944
215	6	0	-5.434409	4.607370	-0.346541
216	6	0	-5.259580	6.048562	0.171974
217	6	0	-4.310761	6.080433	1.368992
218	6	0	-4.738741	6.929947	-0.924028
219	1	0	-3.833721	6.533049	-1.387229
220	1	0	-6.009088	2.139668	-0.900960
221	1	0	-5.022417	3.509473	1.475704
222	1	0	-6.197646	4.584694	-1.136443
223	1	0	-6.234865	6.425584	0.504241
224	1	0	-4.081139	7.113562	1.652666
225	1	0	-4.476184	7.914021	-0.513493

226	1	0	-4.490678	4.289020	-0.811538
227	1	0	-3.361803	5.582153	1.139202
228	1	0	-5.503925	7.122329	-1.677773
229	1	0	-4.755978	5.596737	2.240279
230	7	0	-8.246843	3.666350	0.896428
231	6	0	-9.491556	3.940142	1.617938
232	6	0	-9.482372	3.268133	2.985633
233	8	0	-9.784587	3.896966	4.005819
234	6	0	-10.704379	3.494162	0.791779
235	1	0	-10.730097	4.011954	-0.172930
236	1	0	-8.290584	3.154785	0.022912
237	1	0	-9.549069	5.009576	1.837393
238	1	0	-11.620937	3.732446	1.337405
239	1	0	-10.673974	2.416181	0.611940
240	7	0	-9.147519	1.966582	3.027537
241	6	0	-9.152510	1.254569	4.299060
242	6	0	-8.099592	1.823506	5.245888
243	8	0	-8.330287	1.948986	6.456054
244	6	0	-8.912944	-0.236808	4.056412
245	1	0	-9.671022	-0.646512	3.380668
246	1	0	-8.911355	1.464134	2.180417
247	1	0	-10.107896	1.418857	4.804979
248	1	0	-8.969172	-0.775623	5.004610
249	1	0	-7.925538	-0.405021	3.617874
250	7	0	-6.931758	2.160574	4.711887
251	6	0	-5.849968	2.659663	5.548358
252	6	0	-4.570998	2.731422	4.719560
253	6	0	-3.319000	2.975257	5.494845
254	6	0	-2.472077	2.034458	6.016919
255	6	0	-2.754073	4.248975	5.814042
256	6	0	-1.568614	4.010937	6.539685
257	6	0	-3.137002	5.568853	5.559234

258	7	0	-1.419683	2.653100	6.651361
259	6	0	-0.762492	5.046531	7.010423
260	6	0	-2.334751	6.595448	6.032213
261	6	0	-1.166009	6.328380	6.750434
262	1	0	-0.540819	7.151598	7.085209
263	1	0	-6.795112	2.139493	3.710953
264	1	0	-5.722709	1.982324	6.397959
265	1	0	-6.106428	3.642028	5.964115
266	1	0	-4.697860	3.507687	3.956207
267	1	0	-2.553730	0.956506	6.003794
268	1	0	-4.053853	5.785264	5.017870
269	1	0	-0.608452	2.154199	6.995870
270	1	0	0.149342	4.845918	7.565613
271	1	0	-2.626814	7.625438	5.852659
272	1	0	-4.482043	1.784746	4.173768
273	8	0	-2.452648	1.802497	-2.313827
274	1	0	-3.420985	1.865571	-2.353753
275	1	0	-2.193907	1.988321	-1.373233
276	6	0	2.871060	-7.181387	-5.030833
277	6	0	1.847381	-6.326257	-4.301992
278	8	0	1.736307	-6.381420	-3.068397
279	6	0	4.200791	-6.435260	-5.232517
280	6	0	5.333925	-7.447504	-5.415055
281	6	0	4.458645	-5.490047	-4.066663
282	6	0	6.621937	-6.825287	-5.912874
283	1	0	7.399994	-7.585112	-6.042946
284	1	0	2.476514	-7.538396	-5.989356
285	1	0	3.042293	-8.051893	-4.391337
286	1	0	4.125869	-5.843866	-6.157340
287	1	0	5.014883	-8.224358	-6.123915
288	1	0	3.878685	-4.567713	-4.166855
289	1	0	7.015195	-6.086683	-5.211703

290	1	0	5.506154	-7.960795	-4.459427
291	1	0	5.508268	-5.192973	-4.014993
292	1	0	6.470747	-6.347472	-6.885248
293	1	0	4.195337	-5.938965	-3.120733
294	7	0	1.099895	-5.503079	-5.046657
295	6	0	0.059169	-4.696124	-4.421901
296	6	0	-1.101519	-5.544327	-3.926756
297	8	0	-1.648050	-5.300943	-2.847603
298	1	0	-0.300362	-3.952843	-5.139487
299	1	0	1.444563	-5.213130	-5.951603
300	1	0	0.449795	-4.153774	-3.559573
301	7	0	-1.492659	-6.547987	-4.707937
302	6	0	-2.469733	-7.530446	-4.224228
303	6	0	-1.992296	-8.145534	-2.915746
304	8	0	-2.717462	-8.164965	-1.911416
305	6	0	-2.726499	-8.600319	-5.298460
306	6	0	-3.468578	-7.963910	-6.481503
307	6	0	-3.506886	-9.789158	-4.697948
308	6	0	-3.339890	-8.719755	-7.792560
309	1	0	-3.904864	-8.225992	-8.589330
310	1	0	-0.958362	-6.749114	-5.542096
311	1	0	-3.399538	-7.013448	-3.975750
312	1	0	-1.748804	-8.971271	-5.647756
313	1	0	-3.105405	-6.938771	-6.633091
314	1	0	-2.917719	-10.335019	-3.957628
315	1	0	-3.717525	-9.744647	-7.716380
316	1	0	-4.525569	-7.864805	-6.213210
317	1	0	-3.788841	-10.496490	-5.482499
318	1	0	-2.295113	-8.781091	-8.119958
319	1	0	-4.422608	-9.443861	-4.208099
320	7	0	-0.754832	-8.652184	-2.906503
321	6	0	-0.177742	-9.193086	-1.683037

322	6	0	-0.229871	-8.184939	-0.556320
323	8	0	-0.621168	-8.511394	0.567432
324	6	0	1.274715	-9.608094	-1.924540
325	1	0	1.336050	-10.384931	-2.694710
326	1	0	-0.158915	-8.543188	-3.713144
327	1	0	-0.774001	-10.045943	-1.349041
328	1	0	1.695748	-10.018139	-1.003626
329	1	0	1.880325	-8.750241	-2.226768
330	7	0	0.201905	-6.944615	-0.836656
331	6	0	0.279798	-5.928772	0.201607
332	6	0	-1.046458	-5.768047	0.923843
333	8	0	-1.095199	-5.722806	2.160252
334	6	0	0.724096	-4.586294	-0.404578
335	6	0	1.162456	-3.585831	0.613312
336	6	0	1.143269	-3.625245	1.967513
337	7	0	1.700025	-2.361852	0.271953
338	6	0	1.993678	-1.691662	1.370874
339	7	0	1.663118	-2.432895	2.412975
340	1	0	1.813151	-2.220306	3.392884
341	1	0	0.611044	-6.730066	-1.739729
342	1	0	0.990280	-6.247241	0.970364
343	1	0	-0.087971	-4.173374	-1.012757
344	1	0	0.791123	-4.376686	2.657055
345	1	0	2.465650	-0.723501	1.413854
346	1	0	1.553735	-4.766614	-1.097263
347	7	0	-2.137782	-5.638846	0.168906
348	6	0	-3.433183	-5.445350	0.817833
349	6	0	-3.828113	-6.665823	1.653064
350	8	0	-4.317390	-6.530750	2.787617
351	6	0	-4.505495	-5.141514	-0.226650
352	6	0	-5.900394	-5.117749	0.396855
353	6	0	-5.936347	-4.083636	1.514786

354	6	0	-6.949868	-4.825747	-0.654794
355	1	0	-6.810902	-3.835162	-1.103140
356	1	0	-2.086245	-5.761564	-0.836243
357	1	0	-3.332388	-4.625138	1.528064
358	1	0	-4.456682	-5.910762	-1.009770
359	1	0	-6.113139	-6.102495	0.828046
360	1	0	-6.961789	-3.929126	1.871854
361	1	0	-7.954133	-4.841235	-0.214939
362	1	0	-4.280825	-4.180440	-0.705893
363	1	0	-5.559883	-3.116128	1.168992
364	1	0	-6.936764	-5.573187	-1.455689
365	1	0	-5.345765	-4.405664	2.372360
366	7	0	-3.623617	-7.866418	1.111536
367	6	0	-3.896223	-9.084570	1.883008
368	6	0	-3.172617	-9.032737	3.219071
369	8	0	-3.775104	-9.196798	4.283492
370	6	0	-3.484932	-10.330257	1.081980
371	1	0	-4.039121	-10.387761	0.138914
372	1	0	-3.162753	-7.955579	0.213822
373	1	0	-4.958916	-9.120744	2.137328
374	1	0	-3.707181	-11.226185	1.667541
375	1	0	-2.415603	-10.311585	0.859147
376	7	0	-1.861420	-8.785777	3.180530
377	6	0	-1.075933	-8.823169	4.407238
378	6	0	-1.574458	-7.786806	5.393657
379	8	0	-1.830055	-8.091109	6.566358
380	6	0	0.397817	-8.602233	4.078066
381	1	0	0.774042	-9.393504	3.420229
382	1	0	-1.390765	-8.615973	2.300879
383	1	0	-1.226010	-9.783409	4.909843
384	1	0	0.992254	-8.600020	4.993640
385	1	0	0.541680	-7.639809	3.579195

386	7	0	-1.728890	-6.544100	4.920538
387	6	0	-2.193972	-5.458633	5.769583
388	6	0	-2.287592	-4.168716	4.955253
389	6	0	-2.710343	-3.030295	5.782539
390	6	0	-1.909519	-2.230150	6.543458
391	6	0	-4.046509	-2.553480	5.962505
392	6	0	-3.980852	-1.454037	6.843561
393	6	0	-5.292397	-2.947940	5.464979
394	7	0	-2.664521	-1.279501	7.183811
395	6	0	-5.109307	-0.744633	7.232458
396	6	0	-6.411635	-2.241610	5.849997
397	6	0	-6.315538	-1.153251	6.726452
398	1	0	-7.208448	-0.594736	6.992729
399	1	0	-1.679020	-6.378726	3.922961
400	1	0	-1.500385	-5.329400	6.608511
401	1	0	-3.169338	-5.709611	6.204447
402	1	0	-2.989120	-4.335530	4.130629
403	1	0	-0.838584	-2.295035	6.679475
404	1	0	-5.374384	-3.806914	4.804791
405	1	0	-2.360112	-0.703058	7.952654
406	1	0	-5.044111	0.099448	7.912814
407	1	0	-7.386599	-2.539058	5.478030
408	1	0	-1.308202	-3.978932	4.497525
409	8	0	2.209130	-2.542574	-5.261693
410	8	0	2.019540	2.305030	-5.106759
411	1	0	1.633573	1.426250	-4.940277
412	1	0	1.667087	-1.736781	-5.154518
413	1	0	2.209929	2.580065	-4.187888
414	1	0	2.388276	-2.738875	-4.325419
415	8	0	-2.495820	-2.498075	-2.131732
416	1	0	-2.166740	-3.403509	-2.248315
417	1	0	-2.432746	-2.260168	-1.160780

418	8	0	-1.707964	-1.719737	-5.898300
419	1	0	-1.748436	-1.209745	-5.044808
420	1	0	-0.998589	-1.290666	-6.393097
421	8	0	0.358693	0.139302	3.869806
422	1	0	-0.316982	0.665082	3.418917
423	1	0	1.182811	0.391336	3.427568
424	8	0	-1.568074	-0.468547	-3.526020
425	1	0	-1.835261	-1.234380	-2.940386
426	1	0	-1.888352	0.364971	-3.086060
427	8	0	1.072165	-0.276354	-3.984413
428	1	0	0.108649	-0.343732	-3.760717
429	1	0	1.538747	-0.837368	-3.314408
430	6	0	6.973203	2.970171	-5.361691
431	6	0	6.115875	1.942383	-4.620017
432	8	0	6.203498	1.805292	-3.396546
433	6	0	6.255156	4.335563	-5.510866
434	6	0	7.262949	5.434327	-5.827449
435	6	0	5.480844	4.682545	-4.267714
436	6	0	6.628250	6.820230	-6.137172
437	1	0	7.416261	7.560745	-6.307996
438	1	0	7.278668	2.592458	-6.345021
439	1	0	7.873980	3.103386	-4.755986
440	1	0	5.555854	4.253296	-6.358319
441	1	0	7.885480	5.141157	-6.683871
442	1	0	4.682735	3.966385	-4.052391
443	1	0	6.011501	7.185032	-5.315883
444	1	0	7.946906	5.537978	-4.974206
445	1	0	5.000965	5.658543	-4.361277
446	1	0	6.032466	6.765042	-7.050537
447	1	0	6.133841	4.718039	-3.406358
448	7	0	5.262947	1.223158	-5.341554
449	6	0	4.466858	0.182364	-4.704701

450	6	0	5.314650	-0.987753	-4.241644
451	8	0	5.125571	-1.513003	-3.135537
452	1	0	3.720144	-0.186585	-5.411628
453	1	0	5.008538	1.535684	-6.267236
454	1	0	3.938625	0.583528	-3.836793
455	7	0	6.272133	-1.403212	-5.066653
456	6	0	7.206199	-2.434602	-4.626830
457	6	0	7.935987	-1.963926	-3.373306
458	8	0	8.022721	-2.685551	-2.377456
459	6	0	8.171015	-2.790854	-5.771847
460	6	0	7.409293	-3.555144	-6.858713
461	6	0	9.377928	-3.549975	-5.251617
462	6	0	8.055766	-3.545843	-8.226318
463	1	0	9.060326	-3.982005	-8.213469
464	1	0	6.414885	-0.924749	-5.944502
465	1	0	6.643646	-3.317558	-4.315470
466	1	0	8.530628	-1.838081	-6.197951
467	1	0	6.394484	-3.144675	-6.948085
468	1	0	9.995903	-2.939333	-4.588517
469	1	0	8.149288	-2.527971	-8.624003
470	1	0	7.283796	-4.579550	-6.519348
471	1	0	10.015421	-3.878363	-6.077530
472	1	0	7.464269	-4.122382	-8.944855
473	1	0	9.076680	-4.440360	-4.689713
474	7	0	8.414819	-0.708524	-3.383040
475	6	0	9.128813	-0.170243	-2.228037
476	6	0	8.251845	-0.158226	-0.982847
477	8	0	8.714286	-0.476297	0.116725
478	6	0	9.619730	1.258480	-2.543040
479	1	0	10.158242	1.657090	-1.680545
480	1	0	8.260609	-0.113420	-4.182344
481	1	0	9.972841	-0.821803	-1.988648

482	1	0	8.772445	1.915439	-2.756362
483	1	0	10.303457	1.251921	-3.398317
484	7	0	6.992397	0.257848	-1.126320
485	6	0	6.091666	0.362227	0.019943
486	6	0	5.878804	-0.998679	0.682575
487	8	0	5.834505	-1.103258	1.913383
488	6	0	4.747305	0.951929	-0.435055
489	6	0	3.831072	1.331233	0.688849
490	6	0	4.009432	1.334820	2.031786
491	7	0	2.547393	1.789435	0.472662
492	6	0	1.973502	2.050610	1.633457
493	7	0	2.836680	1.780398	2.596726
494	1	0	2.783750	2.049249	3.582540
495	1	0	6.666109	0.620195	-2.017710
496	1	0	6.553568	0.993741	0.783940
497	1	0	4.237253	0.243368	-1.095315
498	1	0	4.838365	1.031892	2.651416
499	1	0	0.997423	2.488887	1.772188
500	1	0	4.934968	1.832455	-1.058350
501	7	0	5.737903	-2.044232	-0.115711
502	6	0	5.573227	-3.383521	0.458002
503	6	0	6.843605	-3.841766	1.171316
504	8	0	6.778435	-4.447628	2.251269
505	6	0	5.186816	-4.371382	-0.633621
506	6	0	5.197046	-5.802134	-0.080170
507	6	0	3.985033	-5.991004	0.825614
508	6	0	5.269699	-6.825842	-1.186357
509	1	0	4.303824	-6.960315	-1.681124
510	1	0	5.828648	-1.941824	-1.120289
511	1	0	4.809948	-3.325041	1.234115
512	1	0	5.916699	-4.278821	-1.448257
513	1	0	6.092879	-5.928764	0.532017

514	1	0	3.948325	-7.007914	1.234292
515	1	0	5.535114	-7.808160	-0.774549
516	1	0	4.204880	-4.106992	-1.045104
517	1	0	3.062923	-5.828628	0.262279
518	1	0	6.027166	-6.574087	-1.933256
519	1	0	4.001862	-5.309532	1.677285
520	7	0	8.010381	-3.566371	0.580355
521	6	0	9.274350	-3.914058	1.228246
522	6	0	9.384240	-3.218423	2.581470
523	8	0	9.806502	-3.816963	3.576980
524	6	0	10.454675	-3.548623	0.310979
525	1	0	11.391890	-3.826671	0.799450
526	1	0	8.036495	-3.063205	-0.298591
527	1	0	9.282484	-4.982412	1.459713
528	1	0	10.466996	-2.473040	0.114114
529	1	0	10.385092	-4.082530	-0.642325
530	7	0	8.991352	-1.943179	2.643391
531	6	0	9.080694	-1.205696	3.895912
532	6	0	8.088675	-1.731921	4.918896
533	8	0	8.381492	-1.775560	6.119767
534	6	0	8.834418	0.284709	3.649636
535	1	0	9.577414	0.693395	2.956670
536	1	0	8.710908	-1.447344	1.805866
537	1	0	10.064885	-1.368875	4.343217
538	1	0	8.894320	0.829767	4.592046
539	1	0	7.838621	0.444125	3.226360
540	7	0	6.891623	-2.095042	4.466218
541	6	0	5.904171	-2.661376	5.376455
542	6	0	4.581692	-2.856243	4.642317
543	6	0	3.486176	-3.239852	5.557058
544	6	0	2.611973	-2.397971	6.175157
545	6	0	3.141958	-4.564218	5.976708

546	6	0	2.039787	-4.451112	6.847688
547	6	0	3.657321	-5.837876	5.699922
548	7	0	1.734010	-3.117625	6.952343
549	6	0	1.446667	-5.558765	7.447874
550	6	0	3.067817	-6.935430	6.298695
551	6	0	1.973795	-6.789502	7.162517
552	1	0	1.520654	-7.671659	7.604956
553	1	0	6.704410	-2.124248	3.473564
554	1	0	5.774920	-1.979737	6.223557
555	1	0	6.265959	-3.610142	5.791935
556	1	0	4.726105	-3.609648	3.858808
557	1	0	2.541870	-1.320143	6.134751
558	1	0	4.510104	-5.955565	5.036877
559	1	0	1.144221	-2.721699	7.665345
560	1	0	0.595969	-5.455775	8.115368
561	1	0	3.460595	-7.928820	6.102389
562	1	0	4.343100	-1.917441	4.127005
563	8	0	2.406938	-2.033151	-2.384872
564	1	0	3.355448	-1.812910	-2.414317
565	1	0	2.146278	-2.089330	-1.417243
566	8	0	-4.081677	-1.938889	-7.250500
567	1	0	-3.205990	-1.871750	-6.794735
568	1	0	-4.097580	-2.826598	-7.627745

References

1. Penzel, S.; Smith, A. A.; Ernst, M.; Meier, B. H., Setting the magic angle for fast magic-angle spinning probes. *J. Magn. Reson.* **2018,** 293, 115-122. https://doi.org/10.1016/j.jmr.2018.06.002
